# Experimental Characterization of the Hepatitis B Virus Capsid Dynamics by Solid-State NMR

**DOI:** 10.3389/fmolb.2021.807577

**Published:** 2022-01-03

**Authors:** Alexander A. Malär, Morgane Callon, Albert A. Smith, Shishan Wang, Lauriane Lecoq, Carolina Pérez-Segura, Jodi A. Hadden-Perilla, Anja Böckmann, Beat H. Meier

**Affiliations:** ^1^ Physical Chemistry, ETH Zürich, Zürich, Switzerland; ^2^ Institute of Medical Physics and Biophysics, Universität Leipzig, Leipzig, Germany; ^3^ Molecular Microbiology and Structural Biochemistry (MMSB), UMR 5086 CNRS-Université de Lyon, Labex Ecofect, Lyon, France; ^4^ Department of Chemistry and Biochemistry, University of Delaware, Newark, DE, United States

**Keywords:** solid-state NMR, virus, dynamics, relaxation, molecular dynamics

## Abstract

Protein plasticity and dynamics are important aspects of their function. Here we use solid-state NMR to experimentally characterize the dynamics of the 3.5 MDa hepatitis B virus (HBV) capsid, assembled from  240 copies of the Cp149 core protein. We measure both *T*_*1*_ and *T*_*1ρ*_ relaxation times, which we use to establish detectors on the nanosecond and microsecond timescale. We compare our results to those from a 1 microsecond all-atom Molecular Dynamics (MD) simulation trajectory for the capsid. We show that, for the constituent residues, nanosecond dynamics are faithfully captured by the MD simulation. The calculated values can be used in good approximation for the NMR-non-detected residues, as well as to extrapolate into the range between the nanosecond and microsecond dynamics, where NMR has a blind spot at the current state of technology. Slower motions on the microsecond timescale are difficult to characterize by all-atom MD simulations owing to computational expense, but are readily accessed by NMR. The two methods are, thus, complementary, and a combination thereof can reliably characterize motions covering correlation times up to a few microseconds.

## Introduction

Characterizing the dynamics of proteins and their assemblies is often key to understanding their function, and NMR has proven to be particularly suitable to study dynamical behavior at the atomic level. For large systems with a limited monomer size, such as oligomeric proteins and multimeric assemblies like fibrils and sub-viral particles, solid-state NMR is the method of choice, since it allows evaluation of dynamical properties on a per-residue basis. The latter is achieved by measuring NMR relaxation parameters, in particular the ^15^N spin-lattice relaxation *T*_*1*_ that addresses fast motions (correlation times around 1 ns), as well as the rotating frame relaxation, *T*_*1ρ,*_ which probes hundreds-of-nanosecond to millisecond motions (Krushelnitsky et al., 2010; Schanda et al., 2010; Quinn and McDermott, 2012; Tollinger et al., 2012; Lamley et al., 2015; Schanda and Ernst, 2016; Rovó et al., 2019; Shi et al., 2019). The advent of proton detection under fast magic-angle spinning (Agarwal et al., 2014; Andreas et al., 2015, 2016; Penzel et al., 2019; Schledorn et al., 2020) allowed for the sensitive determination of the ^15^N relaxation parameters in a series of two-dimensional (hNH) or, for larger proteins, three-dimensional (e.g. hCANH) experiments (Vasa et al., 2018; Schubeis et al., 2020) to characterize the protein dynamics for each spectrally resolved residue. Important timescales of motion in the context of protein function are slow components, spanning the hundreds-of-nanosecond to low-millisecond time range. While they can be easily accessed in NMR by rotating-frame relaxation measurements, they are difficult to measure by most other techniques.

In the following study, we use solid-state NMR spectroscopy for which, when compared to solution-state NMR, the interpretation of relaxation data is simplified by the absence of overall molecular tumbling. However, the internal protein motions can be rather complex, and the description in terms of an extended model-free approach (Clore et al., 1990; Chevelkov et al., 2009) with several correlation times τ_c_ can be ambiguous (Smith et al., 2017). To mitigate this, the detector analysis approach has been introduced to analyze such data with minimal bias (Smith et al., 2018), as well as to facilitate comparison with molecular dynamics (MD) simulations (Smith et al., 2019; Zumpfe and Smith, 2021).

In contrast to experimental observations of dynamical effects, which are only sensitive to certain windows of correlation times τ_c_, and motions of certain atoms (often the amide proton/nitrogen pairs), MD simulations do not have these restrictions. All-atom MD simulations can provide dynamical details at fully atomic resolution, however, owing to computational expense, sampling timescales are often limited, especially for large protein oligomers or assemblies. With increasing computational power, longer all-atom MD simulations of large systems, including complete virus capsids, have become accessible, revealing insights into functional motions observable over 1 microsecond of sampling (Perilla et al., 2015; Pérez-Segura et al., 2021). However, as simulations must extend over a time significantly longer than the longest correlation time of interest to accurately characterize protein behavior, examination of the most interesting and biologically-relevant motions that occur over multi-microsecond and millisecond timescales remains a challenge.

An all-atom  MD simulation describing the Cp149 HBV capsid (with a number of mutations, vide infra) for 1 microsecond was recently reported (Hadden et al., 2018). While the timescale of the simulation characterizes, with good statistics, motions with correlation times of a few hundred nanoseconds, increasing the sampling time by an order of magnitude to study slower motions represents a significant computational expense. We herein examine HBV capsid dynamics by NMR in order to experimentally test the simulation predictions for the NMR-observable spins and time windows. We show that results of the two methods coincide, demonstrating the validity of using MD simulation data to extrapolate correlation functions for atoms and dynamic timescales which are not observable by NMR.

To shortly describe the objects investigated: HBV particles enclose their genetic material inside mainly T = 4-icosahedral capsids formed by 240 copies of the 183-amino-acid residues core protein (Cp) (Böttcher et al., 1997; Wynne et al., 1999; Yu et al., 2013). The first 149 residues constitute the assembly domain, Cp149, and they are sufficient for *in-vitro* capsid formation (Gallina et al., 1989; Birnbaum and Nassal, 1990). Cp149 forms dimers in solution, and self-assembles, at sufficiently high concentration, into capsids. The asymmetric unit of the icosahedral capsid contains four sequentially but not conformationally identical protein monomers denoted as A, B, C, and D, which are organized as AB and CD dimers (Wynne et al., 1999). The C-terminal 34 residues in Cp183 are disordered and not observed in NMR or cryo-EM (Zlotnick et al., 1997; Wang et al., 2019). The capsid geometry of Cp149 is virtually indistinguishable from the ones formed by the full-length core protein Cp183 (Wang et al., 2019). High-resolution solid-state NMR spectra of Cp149 have previously been described and the ^1^H, ^13^C and ^15^N NMR resonances and have been sequentially assigned (Lecoq et al., 2018, 2019). Indeed, some resonances split into four, representing the four conformationally different core protein monomers, whose structural flexibility is essential to form the capsid.

While the structure of the HBV capsid is well characterized, this is less true for its dynamic behavior; still, it becomes more and more clear that viral capsids are not rigid “tin cans” (Sherman et al., 2020) but plastic and dynamic entities. While plasticity and dynamics are sometimes used interchangeably, we here use plasticity to indicate the deformability of the protein under external forces, as well as its structural flexibility, while we use dynamics to indicate reversible motional processes like backbone- and side-chain motions and conformational exchange between structures (Henzler-Wildman and Kern, 2007). For HBV, the plasticity of the capsid was demonstrated *e.g.* by (Böttcher et al., 2006) by showing that induced stress (by the biochemical introduction of foreign amino-acid sequences at the spike tips) onto the capsid structure could be accommodated by conformational changes still preserving the capsid form. MD simulations of the capsid by Hadden et al. revealed its ability to distort asymmetrically (Hadden et al., 2018) and alter its morphology to further accommodate the binding of small molecules (Hadden et al., 2018). Another indication for structural plasticity comes from the four different conformations of the monomers in the asymmetric unit discussed above and from the recent observation of a conformational switch of the capsid conformation triggered by a pocket factor (Lecoq et al., 2021).

The importance of dynamics for the functional characterization of viruses has recently been demonstrated for the HIV-1 capsid by measuring the dynamical averaging of chemical-shift tensors (Zhang et al., 2016). Plasticity and dynamics are intimately linked to function (Sherman et al., 2020) e.g. in HBV for the transmission of the “maturation signal” (Summers and Mason, 1982; Böttcher et al., 2006) which is believed to trigger interaction with the envelope, and thus virion production once the internal genome is mature. This process thus transmits information on the capsid’s internal genome status to the capsid surface. The mechanism is yet unknown, but must harness conformational plasticity to modulate the envelopment-proficiency of the capsid surface.

In the following, we present an experimental approach to characterize the HBV Cp149 capsid dynamics using the amide nitrogen-proton vector as the probe of dynamics and using amide proton-detected solid-state NMR spectroscopy, allowing the use of fast MAS experiments for characterizing slow motions (Lakomek et al., 2017). We measure data which offer three different observation windows into capsid dynamics: one in the nanosecond range (measuring ^15^N T_1_), and two in the upper nanoseconds-to-microsecond range (measuring ^15^N T_1*ρ*_). We find, using the detectors approach, overall good agreement between MD simulations and NMR for the localized fast motions, yet still with some significant differences. Importantly, we then obtain for the first time a detailed picture also on the microsecond motions of the HBV capsid, which cannot be faithfully predicted from currently available simulation data.

## Materials and Methods

### Sample Preparation

Uniformly ^2^H-^13^C-^15^N labeled Cp149 empty capsids (not containing nucleic acids) were produced as described in (Lecoq et al., 2019) and purified as described in (Lecoq et al., 2018). In brief, Cp149 protein was expressed in *E. coli* BL21 (DE3)*CP strain, grown at 37°C in M9 minimal medium containing 1 g/L of ^15^NH_4_Cl and 2 g/L of deuterated ^13^C-glucose in D_2_O and supplemented with 100 μg/ml ampicillin and 34 μg/ml chloramphenicol. When the OD_600_ reached 0.7, the expression was induced with 1 mM IPTG and the culture was further incubated for 6 h at 25°C.

Harvested cells were resuspended in 15 ml of lysis buffer (50  mM Tris pH 7.5, 300  mM NaCl, 5 mM DTT) and incubated on ice for 45 min with 1 mg/ml of chicken lysozyme, 1x of protease inhibitor cocktail solution and 0.5% of Triton-X-100, then mixed with 4 µL of benzonase nuclease for 30 min at room temperature. Cells were broken by sonication and centrifuged for 1 h at 8,000×*g* to remove cell debris. Cp149 capsids in the supernatant were purified on a stepwise sucrose gradient from 10 to 60% (m/v) sucrose (buffered in 50 mM Tris pH 7.5, 300 mM NaCl) which was centrifuged at 28,800×*g* for 3 h at 4°C. The fractions containing Cp149 were further purified by (NH_4_)_2_SO_4_ precipitation (up to 35% saturation) and finally dialyzed in the solid-state-NMR buffer (50 mM TRIS pH 7.5, 5 mM DTT) overnight at 4°C. 20 µL of saturated 4,4-dimethyl-4-silapentane-1-sulfonic acid (DSS) solution were added to the protein for chemical-shift referencing prior to the sedimentation step. Sub-milligram amounts of capsids were filled into 0.5 and 0.7 mm rotors using home-made filling tools (Böckmann et al., 2009) by centrifugation (200,000 *g*, 17  h, 4°C).

### NMR Spectroscopy, Data Processing and Analysis

Solid-state NMR experiments were recorded using a static magnetic-field strength of 20 T (wide-bore 850 MHz Bruker Avance III Spectrometer) on uniformly (^2^H, ^13^C, ^15^N)-labeled Cp149 capsids sedimented (Bertini et al., 2011, 2012; Gardiennet et al., 2012) in H_2_O to re-protonate amide protons. The 2D hNH spectra for the R_1ρ_(^15^N) determination were measured in a 0.5 mm triple-resonance probe head constructed by Ago Samoson and coworkers (Darklands OÜ, Tallinn, Estonia) (Schledorn et al., 2020), at 80 kHz and 160 kHz MAS frequency, respectively, and at a sample temperature of ∼28°C as determined from the supernatant water resonance frequency (Gottlieb et al., 1997; Böckmann et al., 2009). The 3D hCANH spectra for the R_1ρ_(^15^N) determination were recorded at 80 and 110 kHz MAS in a 0.7 mm triple-resonance Bruker probe, at ∼21°C sample temperature. Both 2D and 3D R_1ρ_ relaxation sequences contain a ^15^N spin lock with an rf-field strength of 13 kHz and a temperature-compensation block before the actual experiment to ensure that the temperature change caused by rf heating is the same for all spin-lock lengths (Lakomek et al., 2017). The variable spin-lock times range from 1 μs to 371 ms for the 2D experiments, and from 1 μs to 251 ms for the 3D experiments. Further details on the hCANH pulse sequence can be found in Supplementary Figure S1. In the case of both 2D- and 3D-based relaxation experiments, one series of measurements was composed of eight experiments with different spin-lock times. The inter-scan delay for the 3D measurements has been set to the optimal value of 2.19 s, based on the bulk protein T_1_(^1^H_N_) time of 1.73 s. All 2D R_1ρ_ measurements were performed at 28°C, and all 3D measurements at 21°C.

The site-specific R_1_(^15^N) relaxation-rate constants were recorded at 108 kHz MAS (0.7 mm Bruker probe head) at ∼28°C using the 2D hNH-based sequence described in ref. (Lakomek et al., 2017). Detailed information about all acquisition parameters can be found in Table S1.

Spectroscopic data has been processed using Topspin 3.5pl6 (Bruker Biospin) with zero filling to the double amount of data points and a shifted sine-bell apodization function in direct and indirect dimensions with SSB = 2.5. Resonance assignments were transferred from earlier work (Lecoq et al., 2019), BMRB accession number 27845, using CcpNmr Analysis 2.4.2 (Vranken et al., 2005; Stevens et al., 2011). Peak positions and peak intensities were then exported to MATLAB (MATLAB R2019a, 9.6.0.) for the relaxation analysis.

### Relaxation Analysis

Site-specific relaxation-rate constants were obtained within MATLAB using the spectral fitting package INFOS(Smith, 2017), error bars were obtained using bootstrapping methods and are reported throughout the work as twice the standard deviation, 2σ . Relaxation curves were fitted with a mono-exponential fit with two degrees of freedom ( A⋅exp(−Rt) ) and are shown for all studied resonances in 2D and 3D experiments in Supplementary Figure S2–5.

### Detector Approach for Comparison of Amplitudes and Correlation Times of Motion From NMR Measurements and MD Trajectories

The detector approach (Smith et al., 2017, 2018, 2019) was used to extract, from the experimental NMR data as well as from the MD trajectory, site-specific amplitudes of motion over ranges of timescales quantified by detector responses $\rho_{i}^{\left(\theta ,\;S\right)}$ . The general procedure is discussed in detail in (Smith et al., 2018), and is briefly introduced in the results section as well as illustrated in Figure 4A. For the computation of the detector responses the DIFRATE software (Smith et al., 2018) was employed. Construction of optimized detector sensitivities $\rho_{i}$ was performed using the SVD_auto() command, which is based on a singular-value decomposition and ensures, first, the best reproduction of the experimental rate constants and therefore maximum extraction of information from the experimental data. Second, SVD_auto () requires non-negativity of sensitivities, and finally maximizes the possible spread between the maxima of the sensitivities. Finally the experimental data is inserted together with the optimized detector sensitivities into the fit_data () function, which outputs the detector responses $\rho_{i}^{\left(\theta ,\;S\right)}$ . The error on the experimental detector response is related to the experimental error and is calculated as well by the fit_data () function.

**FIGURE 1:**
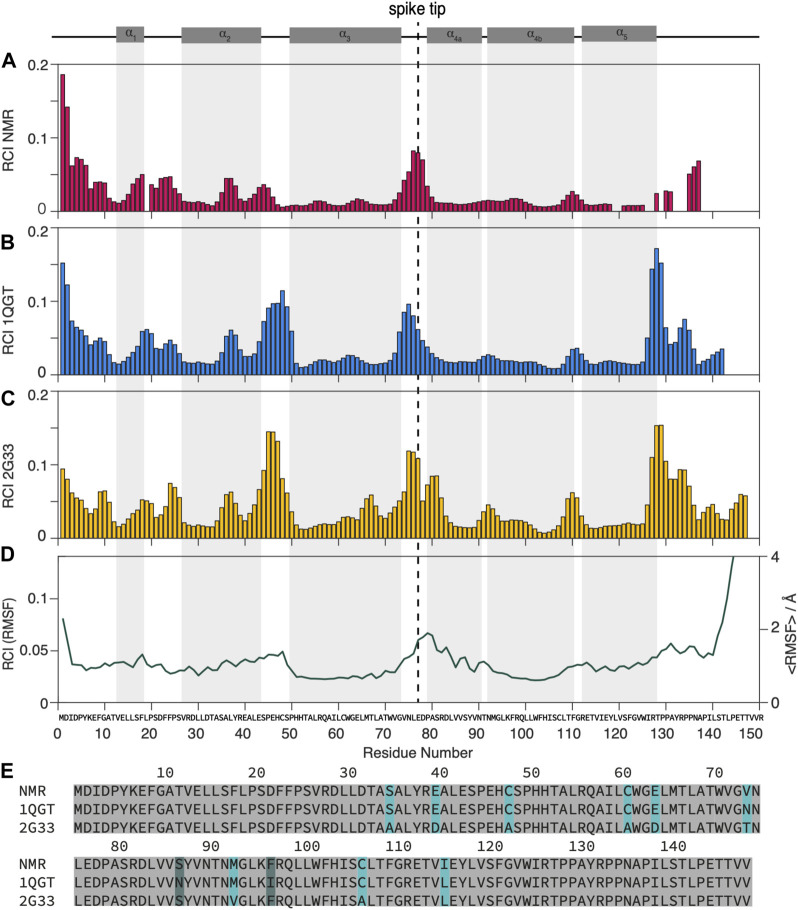
Dynamic and static regions as distinguished by the random  coil index.**(A)** RCI from experimental chemical-shift data (Lecoq et al., 2018, 2019), **(B)** from the X-ray structures used for NMR (PDB 1QGT) and **(C)** for MD (PDB 2G33). **(D)** RMSF from the MD trajectory (Hadden et al., 2018) (left scale) and RCI calculated from the RMSF (right scale) by multiplying it by an empirical factor of 28.3 A according to (Berjanskii and Wishart, 2008). **(E)** Alignment of the different strains used: strain D for NMR, strain CW for the 1QGT crystal structure, strain ADYW for the 2G33 crystal structure and the MD calculations. The mutations between strains D and ADYW (NMR and MD) are highlighted in cyan.

An all-atom MD simulation provides the coordinates of each atom as function of time, and allows the calculation of residue-specific relaxation-rate constants from the autocorrelation function C(t)  of the dipolar interaction between the two spins in the backbone amide bond. The trajectory, taken from (Hadden et al., 2018)*,* describes the Cp149 HBV capsid over 1 microsecond of sampling time, with coordinate frames saved every 20 ps. The capsid was studied under conditions that mimic its physiological environment: temperature of 37°C, pH of 7.0, and explicit solvent containing 150 mM NaCl. The capsid simulation was based on the crystal structure PDB 2G33 (Bourne et al., 2006) and, in addition to being another genotype of HBV contains three mutations: C48A, C61A, and C107A. In total, nine amino-acids are substituted (see Figure 1 and Supplementary Figure S15). The principal vectors of chemical-shift anisotropy (CSA) of the ^15^N are not exactly co-linear with the ones of the N-H dipole coupling, so that its motion may not be described with exactly the same autocorrelation function *C*(*t*), although the two are sufficiently similar, so that we take them to be equal. C(t) can contain contributions from motions at different timescales. With an inverse Laplace transform it is, in principle, possible to find the underlying distribution of motion (amplitudes of motion at different timescales). It is well known that the inverse Laplace transform is ill-posed and that regularization methods are often indispensable. It has however been shown in (Smith et al., 2019) that this poses no severe problems for the subsequent detector analysis, *i.e.* the obtained detector responses are to a large degree well-behaved (see SI Section S1 for further discussion). For the inverse Laplace transform, we still explored the effect of using a regularization function, ensuring the smoothness of the output distribution of motions (minimizing its second derivative). The influence of the regularization weight λ at different timescales is subject to a detailed discussion in the SI Section S1.1. All site-specific MD simulation correlation functions, as well as corresponding distributions of motion, were collected in Supplementary Figure S6, S7. This approach allowed us to determine the overlap between the distribution of motion observed with MD simulation and the experimental detector sensitivities, as well as to obtain the MD simulation detector responses, which can be directly compared to the detector responses from the experimental NMR data.

## Results

### A Rough Overview: Random Coil Index

A first overview over dynamic and rigid regions of the capsids is provided by the random coil index (RCI) (Berjanskii and Wishart, 2005, 2008; Fowler et al., 2020), an empirical quantity calculated from the NMR chemical shifts. While the RCI does not provide detailed information about the timescale of the dynamics, it specifies the amplitude of the motion and distinguishes dynamic and rigid regions, allowing for an overview of the molecule’s dynamic behavior. The RCI values from the experimental NMR data were calculated using H_N_, ^15^N, ^13^C_α_, ^13^C_β_, and C′ chemical shifts from (Lecoq et al., 2018, 2019) and are shown in Figure 1A. The RCIs from the X-ray structures are calculated by converting the X-ray data to chemical shifts using the software SHIFTX2 (Han et al., 2011) RCI values from the X-ray structures of the genotype used for NMR (PDB 1GQT) and MD (PDB 2G33) are shown in Figures 1B, C respectively and compared to the root mean square fluctuation (RMSF) from MD (from (Hadden et al., 2018) in Figure 1D.

Although some significant differences are observed (mainly in the loop from residues 44 to 49 and towards the C-terminal around 130), overall the RCIs calculated from the experimental NMR data match well those calculated from the X-ray structures and they highlight similar dynamic regions than the MD RMSF. The RCI method is an experimentally rather straightforward approach and requires only resonance assignment. For a detailed dynamic characterization, particularly disentangling the timescales of the underlying motions, NMR relaxation-rate constants need to be evaluated.

### NMR Relaxation Measurements

**FIGURE 2:**
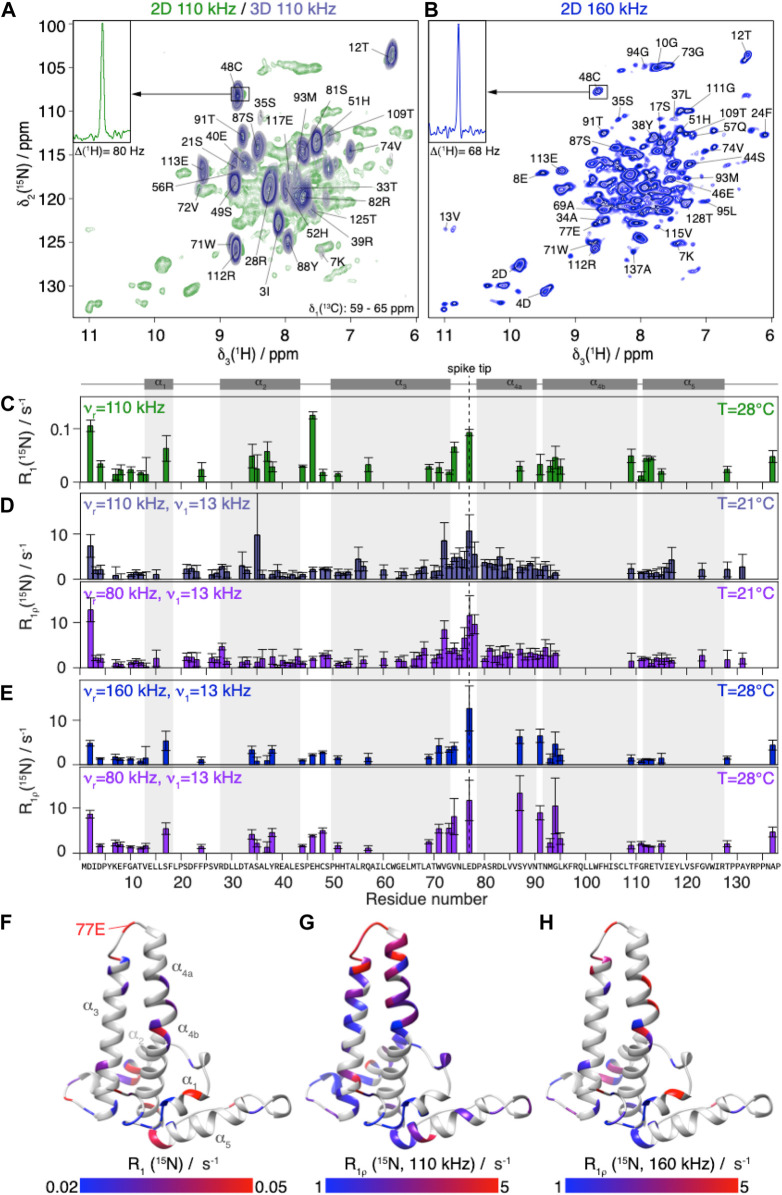
NMR ^15^N relaxation-rate constants of Cp149 capsid.**(A)** Overlay of Cp149 2D hNH spectrum (green) and slices of 3D hCANH (δ(13C) from 58.95 to 65.51 ppm, steel blue) recorded at 110 kHz MAS. The proton line of 48C in the 2D spectrum is shown in the insert.**(B)** 2D hNH spectrum of Cp149 capsids recorded at 160 kHz MAS. The proton line of 48C is shown in the insert. The assignment is transferred from Lecoq et al. (Lecoq et al., 2019). Experimental site-specific values of (**C)** R_1_(^15^N, 86 MHz) measured at 110 kHz,**(D)** R_1ρ_(^15^N) measured at 110 kHz (steel blue) and 80 kHz (purple) MAS using *ν*_1_ = 13 kHz spin-lock with the 3D hCANH in the 0.7 mm rotor and **(E)** R_1ρ_(^15^N) measured with 2D hNH at 160 kHz (blue) and 80 kHz (purple) MAS in the 0.5 mm rotor using ν _1_ = 13 kHz spin-lock. The error bars depicted are calculated using a bootstrapping method and are given by 2σ . The Cp149 capsid secondary structure is displayed in grey on top of the plots and the amino-acid sequence at the bottom. **(F)** Site-specific experimental R_1_(^15^N, 86 MHz) values measured at 110 kHz plotted from 0.05 to 0.02 s^−1^ in a red to blue gradient. **(G)** R_1ρ_(^15^N, 110 kHz) and **(H)** R_1ρ_(^15^N, 160 kHz) values measured using *ν*_1_ = 13 kHz spin-lock, plotted from 5 to 1 ^s−1^ in a red to blue gradient on a single monomer of Cp149 capsid (PDB 1QGT).

The solid-state NMR relaxation measurements were performed on assembled HBV capsids formed from the N-terminal assembly domain (Cp149). Figure 2A shows, in green, a 2D hNH spectrum recorded at 110 kHz MAS in a 0.7 mm rotor at 20.0 T magnetic field. The spectrum shows the expected good spectral resolution, with proton linewidths in the order of 110 ± 50 Hz as reported previously (Lecoq et al., 2019). To characterize a higher number of residues by reducing spectral overlap, 3D hCANH-type relaxation experiments were recorded. We obtained two sets of eight R_1ρ_(^15^N) 3D spectra, at 80 kHz and 110 kHz MAS (and 21°C), using optimized acquisition parameters to fit each spectrum into one single day of measurement time (for details on the optimization see Supplementary Figure S8). A series of 2D slices (δ(^13^C) from 59 to 65 ppm) of the first 3D hCANH spectrum in the series (with essentially zero relaxation delay) is shown in steel blue in Figure 2A (for all slices see Supplementary Figure S9). In the 3D hCANH spectra, 73 peaks could be resolved (Figure 2D).

In order to extend the time windows of the relaxation analysis, we also recorded hNH-type relaxation experiments in an 0.5 mm rotor at currently fastest MAS frequencies (160 kHz) at 28°C, shown in Figure 2B. A representative trace (48C in the inset in Figure 1A, B) reports the concomitant linewidth improvement from 80 to 68 Hz achieved thanks to the higher spinning frequency (Schledorn et al., 2020). In these 2D spectra, 35 well-resolved backbone amide proton/nitrogen peaks, labelled in Figure 2B, were used for the relaxation analysis of the data, roughly half as many site-specific R_1ρ_(^15^N) rate constants as from the three-dimensional experiments due to peak overlap. $R_{1\rho }$ spectra were also recorded in the same rotor at 80 kHz MAS. The recording of 3D data has proven too time-consuming under these conditions (SNR_0.5 mm_/SNR_0.7mm_ = 50%, see Supplementary Figure S10).

R_1_ (^15^N) relaxation was investigated using hNH-based experiments at 110 kHz MAS and 28°C (being to a large degree MAS-frequency independent). The relaxation-rate constants resulting from the analysis of the R_1_(^15^N) measurements are shown in Figure 2C. The rate constants contain information for each individual residue about motions of the NH vector on a timescale on the order of the inverse Larmor angular frequency for ^15^N, 1.8 ns. The secondary structure of the protein shown in grey above the graph indicates that the fastest relaxing residues are located in loops; for a more detailed discussion see below where the resulting detector response is interpreted. In general, relaxation-rate constants of roughly 0.1 s^−1^ and smaller are observed, which are very similar to values reported for rigid proteins studied under similar conditions, as for example microcrystalline ubiquitin, where however the dynamic *β*_1_-*β*_2_ turn (residues 10–12) relaxes faster (Ma et al., 2015; Lakomek et al., 2017). Other examples from the literature include (Knight et al., 2012; Lamley et al., 2015; Smith et al., 2016). This observation indicates that the capsid is quite rigid on the nanosecond timescale.

The R_1ρ_ (^15^N) relaxation-rate constants resulting from the analysis of the 3D hCANH spectra are shown in Figure 2D. They are mostly below 10 s^−1^ at 110 kHz, and slightly higher at the lower MAS frequency, 80 kHz. R_1ρ_(^15^N) rotating-frame relaxation-rate constants inform about motions at frequencies of the spin-lock frequency plus or minus once or twice the MAS frequency: $\nu_{1}\pm \nu_{r}$ and $\nu_{1}\pm 2\nu_{r}$ (with $\nu_{1}$ = 13 kHz and $\nu_{r}$ = 80 or 110 kHz). Thus, as typically $\nu_{1}\ll \nu_{r}$ , these experiments probe motions at correlation times of $\tau_{c}={\left(2\pi \nu_{r}\right)}^{-1}$ and $\tau_{c}={\left(4\pi \nu_{r}\right)}^{-1}$ , corresponding to values in the low microsecond range. Again, they are comparable to microcrystalline systems (Lakomek et al., 2017).

The relaxation-rate constants resulting from the analysis of the 2D hNH spectra at 160 and 80 kHz MAS at slightly higher temperature (28°C instead of 21°C) are shown in Figure 2E. Despite the overall rather rigid behavior on the nano as well as microsecond scale, we indeed find significant differences between the backbone-relaxation properties of the different residues for all five relaxation-rate constants determined. The fastest R_1ρ_ (^15^N) relaxation is observed for residues around 77E (the spike tip) and for some residues at the spike base, as illustrated in the color-coded representations of the rate constants on the capsid monomer given in Figures 2F–H.

**FIGURE 3:**
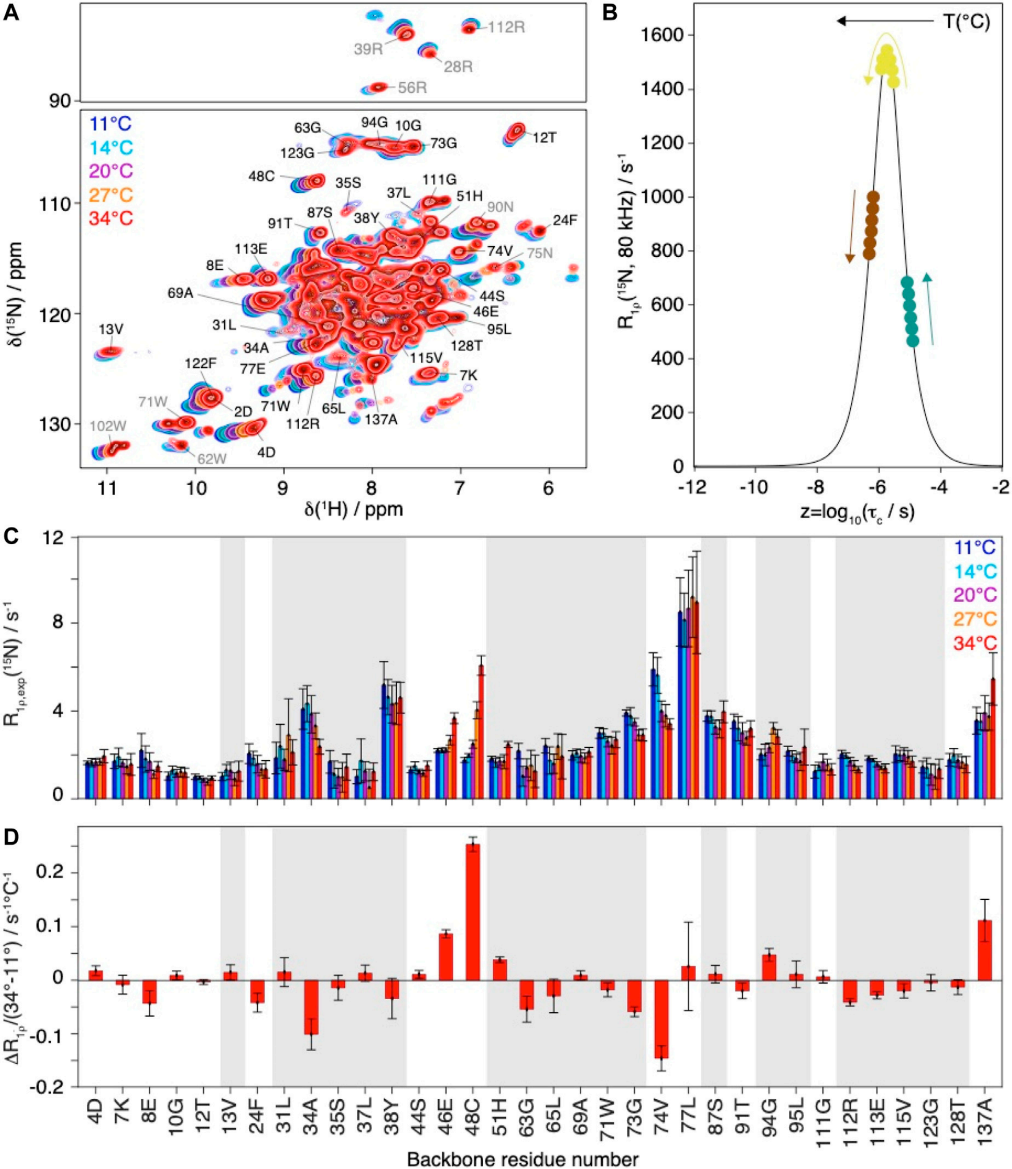
Evolution of Cp149 relaxation-rate constants as function of the temperature. **(A)** Overlay of 2D hNH spectra of Cp149 capsids recorded at 80 kHz MAS, 28 scans and 15.8 ms acquisition in the ^15^N dimension at 11°C (blue), 14°C (cyan), 20°C (purple), 27°C (orange) and 34°C (red). The assignment is transferred from Lecoq et al. (Lecoq et al., 2019). **(B)** Normalized analytical R_1ρ_ (^15^N, *ν*_r_ = 80 kHz, *ν*_1_ = 13 kHz) rate constant as function of the correlation time. The colored dots and arrows indicate the evolution of the rate constant with temperature depending on its original position on the curve. **(C)** Selected experimental site-specific values of R_1ρ_ (^15^N, *ν*_r_ = 80 kHz, *ν*_1_ = 13 kHz) measured between 11°C (blue) and 34°C (red) for the backbone residues and (**D)** Site-specific gradients for the change in relaxation rate constant between 34°C and 11°C, ΔR_1ρ_/ΔT = (R_1ρ_(34°C) - R_1ρ_(11°C))/(34°C-11°C). The error bars have been calculated with Gaussian error propagation. In both graphs the bars for residues 2D and 122F have been marked by an (*), due to the relaxation data being biased by the overlap of the two resonances in the hNH at higher temperatures. Grey shaded areas indicate α - helical secondary structure.

MD simulations have been performed at 37°C, while NMR relaxation measurements could not be conducted at these temperatures, due to limited protein stability over time. In order to assess the temperature-dependent R_1ρ_(^15^N) rate constants in a site-resolved manner, we measured the faster-to-record two-dimensional spectra at different temperatures between 11°C and 34°C at 80 kHz MAS (Figure 3A).

The relaxation-rate constants for resolved backbone amide ^H^N spins are shown in Figure 3C. In a schematic picture, Figure 3B illustrates that, while motion becomes faster with increasing temperature, the corresponding relaxation-rate constants can increase, decrease or go through a maximum. As discussed above NMR rotating-frame rate constants under MAS become particularly large for motions on timescales with corresponding frequency approaching the order of magnitude of the inverse spinning, typically corresponding to values in the low microsecond timescale. For residues with characteristic motions slower than microseconds, the increase of temperature shifts them closer to the timescale regime which leads to a larger rate constant response under the experimental conditions and would result in a monotonic increase in measured rate constant. Experimentally, we observe all three scenarios (Figure 3): some rate constants increase with increasing temperature (*e.g.* residues 46E, 48C, 51H, 137A), some show signs of increase followed by a decrease (*e.g.* residues 37L, 94G and 111G) and some decrease with increasing temperature (*e.g.* residues 8E, 24F, 34A, 74V, 112R, 113E, 115V). The temperature dependence is further illustrated by comparing the difference in the relaxation-rate constants between 11°C and 34°C in Figure 3D. One can see that most gradients are small, below 0.05°C^−1^ s^−1^. A strong increase in relaxation-rate constants with temperature is observed for residues 46E, 48C, and 137A, all located in loop regions. These residues thus show motions with correlation times above 1.9 µs (maximum of the curve in Figure 3B. The α -helical residues 34A, 63G, 73G, and 74N show a significant decrease in relaxation-rate constants, concomitant with correlation times below 1.9 µs, and thus faster motions than the loop residues discussed above. Residues 94G and 111G first significantly increase and then decrease in Figure 3C, suggesting that they might have a correlation time close to the maximum, *i.e.* 1.9 µs.

### Detector Analysis of NMR Relaxation Data and MD Simulations

**FIGURE 4:**
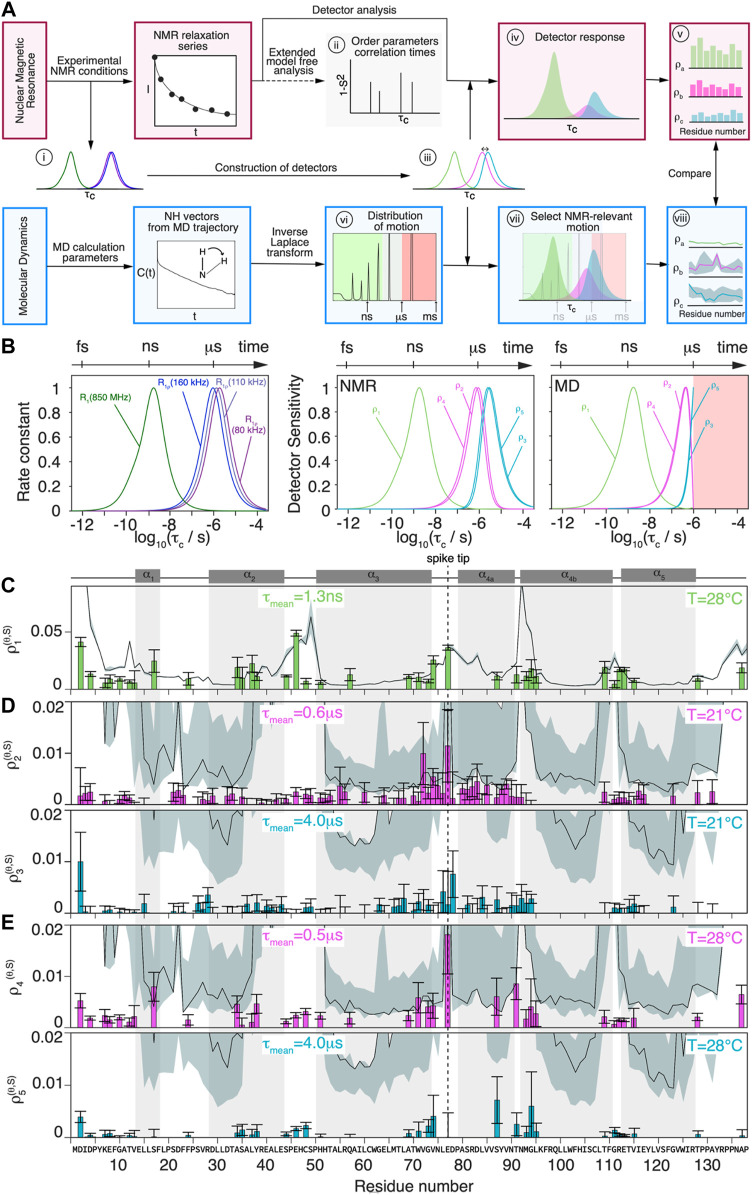
Comparison of  MD simulation and NMR dynamics detector responses. **(A)** Schematic flowchart of the method used to obtain detector responses from the NMR data (orange boxes) and MD  simulation trajectory (blue boxes). **(B)** Normalized analytical R_1_(^15^N, 86 MHz) and R_1ρ_ (^15^N, *ν*_r_ = 160/110/80 kHz, *ν*_1_ = 13 kHz) rate constants as function of the correlation time (left) and detector sensitivities constructed from linear combination of analytical rate constant functions depicted beside, where $\rho_{2}\;$ and $\;\rho_{3}\;$ corresponds to the linear combination of R_1ρ_ (^15^N, *ν*_r_ = 110/80 kHz, *ν*_1_ = 13 kHz) and $\rho_{1},\;\;\rho_{4}\;$ and $\rho_{5}\;$ to a linear combination of R_1ρ_ (^15^N, *ν*_r_ = 160/80 kHz, *ν*_1_ = 13 kHz) and R_1_(^15^N, 86 MHz). Optimization of detector sensitivities for analysis of the MD have been restricted to timescales up to 1 μs . The axis on top represents the corresponding timescale.**(C)** Overlay of corresponding site-specific $\rho_{1}^{\left(\theta ,\;S\right)}$ detector response calculated from experimental data (bar plots, with the corresponding error bars calculated using a bootstrapping method) and detector responses from simulation (black line, regularization weight λ=0 ), trajectory from (Hadden et al., 2018) for chain A of the HBV Cp149 capsid. Overlay of corresponding site-specific **(D)** $\rho_{2}^{\left(\theta ,S\right)}$ (pink) and $\rho_{3}^{\left(\theta ,\;S\right)}$ (cyan) detector responses and **(E)** $\rho_{4}^{\left(\theta ,\;S\right)}$ (pink) an $\rho_{5}^{\left(\theta ,\;S\right)}$ (cyan) detector responses calculated from experimental data (bar plots, with the corresponding errors bars calculated using a bootstrapping method) and detector responses from simulation (black line) for chain A of the HBV Cp149 capsid. The response for subunits B-D are shown in Supplementary Figure S11. The error estimate (obtained as described in Supplementary Information section S1) for MD simulation data is shown as the grey shaded area; the MD simulation was performed at 37°C.

In order to compare the NMR relaxation-rate constants with the MD simulation, a detector analysis was performed on both data sets.(Smith et al., 2019). The general procedure is summarized in Figure 4A, distinguishing NMR experiments and MD simulation with red and blue boxes respectively.

The NMR relaxation-rate constants are sensitive to the motion of an N-H vector with a correlation time *τ*_c_ at a certain amplitude as shown in panel (i) of Figure 4A (note the logarithmic scale in *τ*_c_). If the order parameter S, which characterizes the amplitude of the motion, is known, *τ*_c_ can in principle be determined from the measured ^15^N relaxation-rate constants $R\left(\nu_{r},\nu_{1},\nu_{0}\right)$ ( $\nu_{r}$ denotes the MAS frequency, $\nu_{1}$ the rf-field amplitude (for $R_{1\rho \;}$ only) and $\nu_{0}$ the Larmor frequency (for $R_{1}$ only in good approximation)). However, in a complex (bio-)molecule, several correlation times *τ*_c_ describe the motion, and their extraction from experimental relaxation-rate constants (Figure 4A (ii)) becomes an ill-posed problem. (Smith et al., 2017). In such a case, the different amplitudes of motions of the NH vectors can be quantified by analyzing the relaxation-rate constants as responses of detectors $\rho_{n}^{\left(\theta ,S\right)}$ defined by the experimental NMR conditions and representing the sensitivity of the experiment to motion with $\tau_{c}$ . For a single relaxation experiment performed, the *sensitivity* of such a detector n to a dynamic process characterized by *τ*_c,_ corresponds to the calculated value of the rate constant discussed above, $\rho_{n}(\tau_{c}$ ) $\propto R\left(\nu_{r},\nu_{1},\;\nu_{r}\right)$ , as illustrated by the individual green, blue and purple curves schematized in Figure 4A panel (i). An NMR detector has its maximum sensitivity at a certain *τ*_c_, and covers a range of about one order of magnitude in correlation time. Experimentally, the frequencies $\nu_{r},\nu_{1},\nu_{0}$ can be varied to a certain extent, within the limits of spinning, probe-head and magnet technology. This then allows the construction of a maximum of *m* detectors from a linear combination of *m* experiments, as shown in panel (iii) of Figure 4A. Experimental conditions are chosen such that they cover the largest possible range of correlation times in the time window of interest, which is then further maximized by computing the optimal linear combination, finally resulting in the detectors used for the data analysis.

For a motion with an arbitrary distribution $\text{Θ}\left(\tau_{c}\right)$ of correlation times, the detector responses $\rho_{n}^{\left(\theta ,S\right)}$ are the sum of all motions inside the detector window (panel 4) in Figure 4A), weighted with the sensitivity of the detector. Note that detector responses are unitless. The integral $\rho_{n}^{\left(\theta ,S\right)}=\;\overset{\infty }{\underset{0}{\int }}\theta \left(\tau_{c}\right)\rho_{n}\left(\tau_{c}\right)d\tau_{c}$ can thus be established for every single residue of the protein with a resolved signal (panel (v) in Figure 4A). To compare NMR with MD simulation data, we extract from the simulation trajectory the motions to which NMR is sensitive by a detector analysis (Smith et al., 2019) (panels (vii)-(viii) in Figure 4A). MD simulation and NMR can then be compared by means of their respective detector responses.

Figure 4B (left) shows the calculated relaxation-rate constant as function of $\tau_{c}$ for the conditions of the five experiments of Figure 2. A linear combination of the relaxation-rate constants then gives the detector sensitivities $\rho_{n}$ shown in Figure 4B (right), which together with the NMR and MD simulation data yield the detector responses $\rho_{n}^{\left(\theta ,S\right)}$ . Note that the two maxima of the NMR detector sensitivities ( $\tau_{\text{mean}}$ ) are more separated than the curves before linear combination. This allows to better take advantage of the range of timescales to which the experiments are sensitive, because a higher timescale resolution is obtained and it becomes possible to better distinguish faster from slower motions. $\rho_{1},\rho_{4},\rho_{5}$ denote the detector sensitivities constructed from the ^15^N rate constants R_1_ (110 kHz, 86 MHz), R_1ρ_(160 kHz, 13kHz), R_1ρ_(80 kHz, 13 kHz), and which were used to analyze the two-dimensional data sets recorded at 28°C. $\rho_{2},\rho_{3}$ obtained from R_1ρ_(110 kHz, 13 kHz) and R_1ρ_(80 kHz, 13 kHz) were used in the analysis of the three-dimensional data sets obtained at 21°C.

The MD detector sensitivities have been obtained allowing an optimization only for a correlation timescale range up to 1 μs (until the red area in Figure 4B (right)), since extrapolating dynamic data beyond the length of the trajectory should lead to unphysical results. The MD microsecond detectors are slightly shifted compared to the NMR detector, due to the restriction of the optimization timescales to 1 μs . For comparing NMR to MD, we will compare results from the slower detectors and faster detectors amongst each other.

The detector responses are plotted in Figures 4C–E for all residues that could be evaluated. The detectors used are most sensitive to motions on the nanosecond timescale for R_1_ and on the microsecond timescale for R_1ρ_. While the former can be directly compared with the detector responses from the MD simulation, the latter lies, for MD, right at the edge of the sampled timescale. It is clear that the simulation predictions lose their statistical relevance once the timescale approaches the length of the trajectory. We estimate the reliability of the data by an error analysis presented in the Supplementary Information section S1. This information is represented, together with the detector responses from MD simulation, as grey area (error) and solid black line (response) in Figures 4C–E.

### Comparison of NMR Relaxation Rates and NMR Detector Response

The detector response of $\rho_{1}^{\left(\theta ,\;S\right)}$ displayed in Figure 4C is almost identical to the R_1_(^15^N) data (Figure 2C), except that a renormalization factor has been applied. This is expected since the R_1_ curve does not overlap significantly with the R_1ρ_ curves (Figure 4B), and thus the $\rho_{1}\;$ sensitivity is approximately a renormalization of the R_1_ sensitivity. Large $\rho_{1}^{\left(\text{θ},\;S\right)}$ detector responses (Figure 4C) are thus observed for the residues with high R_1_ relaxation-rate constants caused by fast (wiggling) motions with $\tau_{c}$ in the nanosecond timescale. Large values were found for several loop residues, including the N-terminal (2D), the loop between $\alpha_{2}\;$ and $\alpha_{3}$ (46E), the spike tip (74 V, 77E) and towards the C-terminal (137A), but also 17S in the $\alpha_{1}$ helix and 34A and 38Y located at the kink of the $\alpha_{2}$ helix. 46E is in fact the fastest relaxing residue for the R_1_ relaxation-rate constant regime. Somewhat unexpectedly this residue points into the lumen of the capsid.

For the slower motions, the corresponding detector responses $\rho_{2}^{\left(\theta ,\;S\right)},\rho_{3}^{\left(\theta ,\;S\right)}$ from measurements at 21°C (Figure 4D) follow a similar behavior as the R_1ρ_(^15^N, 80/110 kHz) rate constants. This is expected, since the detector responses result from a combination of the significantly overlapping R_1ρ_(^15^N, 80/110 kHz) relaxation-rate profiles (see Figure 4B). This means that the relative intensity of the two slow detector responses depends on the difference between the measured R_1ρ_ rate constants at the two spinning frequencies. For example, residues 127R and 132Y show mostly detector responses in $\rho_{2}^{\left(\theta ,\;S\right)}$ since their relaxation-rate constants do not depend significantly on the spinning frequency, which is a characteristic feature of faster microsecond motions (Supplementary Figure S12). The highest detector response for both correlation times $\rho_{2}^{\left(\theta ,\;S\right)}\;$ and $\;\;\rho_{3}^{\left(\text{θ},\text{S}\right)}$ is detected for residue 77E on the spike tip.

Many mobile residues are located in the tip of the spike (residues 73G to 79P, see Figure 2F). The residues forming the lumen of the capsid, in contrast, stay rather rigid (e.g. 7K-10G, 111G-115V), with the exception of 46E. Other areas of elevated amplitudes of motion on the microsecond timescales are found at the N-terminal part of the protein, in the loop regions between helices $\alpha_{1},\;\alpha_{2}$ , $\alpha_{3}$ and at the beginning of loop 5. The distribution of these slower motions being concentrated in specific areas moving in a similar and correlated manner is actually expected, in contrast to the local nature of faster wiggling motions described by the $\rho_{1}^{\left(\text{θ},\text{ S}\right)}$ detector response.

The detector responses from the 2D measurements at 80/160 kHz MAS at 28°C are shown in Figure 4E. Due to the larger separation of spinning frequencies (80/160 kHz), the resulting two detectors are further apart: $\rho_{4}^{\left(\text{θ},\text{ S}\right)}$ and $\;\rho_{5}^{\left(\text{θ},\text{S}\right)}$ (an alternative description using the $\rho_{2}^{\left(\text{θ},\text{ S}\right)},\;\rho_{3}^{\left(\text{θ},\text{S}\right)}$ detectors is given in Supplementary Figure S13). These spectra recorded at a higher temperature of 28°C give an indication on the temperature dependence of the dynamics. A comparison between Figures 3E, 4D shows a similar behavior for the residues determined in both series. The data confirms that the tip of the spike (residue 77E) reveals most dynamics on the faster timescale (0.5 or 0.6 µs), which is as well the case for 17S and 137A, showing the largest detector response in the nanosecond regime (Figure 4C) and on the 0.5 μs  timescale (Figure 4E), while almost no motion on the  4.0 μs timescale.

### Comparison of NMR and MD Detector Responses

One can compare the detector responses obtained by NMR and MD simulation in Figures 4C–E, where simulation detector responses are given as a solid black line, with error estimates shown as grey shaded areas. As expected, the error is substantially larger for the microsecond than for the nanosecond MD detector responses.

For the $\rho_{1}\;$ detector, the responses from MD simulation and NMR (Figure 4C) are similar for many residues, indicating good agreement considering that there are no adjustable parameters in the detector analysis. These results, thus, globally validate the MD simulation on an experimental basis. A few quantitative differences were however observed, *e.g.* in the range of residues 2D to 14E and, in particular, 2D and 12T which show, consistently, a smaller NMR detector response than predicted by the simulation. In addition, with the exception of residue 46E, the NMR data do not reveal an enhanced nanosecond motion as predicted by simulation in the loop from residues 43E-51H connecting the $\alpha_{2}$ and $\alpha_{3}$ helices. According to experiment, residue 93M is much less dynamic than predicted in simulation. The short loop or kink in helix 4 (between $\alpha_{4a}$ and $\alpha_{4b})$ is predicted to yield a high detector response at 1.3 ns, but NMR does not detect it. It is noticeable that the root-mean-square fluctuations, considering the entire frequency spectrum, do however not show particularly high flexibility for this loop (see Supplementary Figure S14, data from (Hadden et al. 2018)). The question arises whether some of these differences could originate from the different temperatures of NMR and MD simulation. The temperature-dependent relaxation constant measurements (Figure 3F) indicate however that we do not observe any strong temperature dependence in the experiments. *E.g.* 48C shows one of the largest gradients for the relaxation rate-constant changes with temperature ( $0.25\;s^{-1}{}^{\circ}C^{-1}$ ). Still, we predict only a factor of two when going from 28°C to 37°C, whereas a factor of over ten is observed by NMR.

The comparison for the MD simulation and NMR predictions at the microsecond timescales ( $\rho_{2}^{\left(\text{θ},\text{ S}\right)}\;$ and $\rho_{3}^{\left(\theta ,\;S\right)}$ respectively) in Figure 4D show similar qualitative and quantitative agreements for the spike area. However, we also note that the simulation error estimation is significantly larger than in the nanosecond case, making the simulation predictions for these timescales more error-prone (detectors with a mean correlation time at the edge of the trajectory). Again, the regions between $\alpha_{2}$ and $\alpha_{3}$ and between $\alpha_{4a}$ and $\alpha_{4b}$ are predicted to be much more flexible than observed by NMR. An exception is the spike position, where prediction and experiment agree rather well (region between residues 50C and 85V).

### Assessing the Impact of Sequence Substitutions on NMR Versus MD Detector Responses

Differences in the Cp149 amino-acid sequence could account for part of the disparities in detector responses used to compare NMR and MD simulations for sub-microsecond time: nine residues are different in the MD (strain ADYW) and NMR (strain D) samples (see Figure 1E and Supplementary Figure S15) representing a 6% change in sequence identity. In Figure 1, the difference in RCI between the two X-ray structures which are different in nine residues show only small differences. Still such substitutions may slightly reduce the thermodynamic stability of the simulation model, based on a decrease of up to 5% in the predicted melting temperature index (Ku et al., 2009). The latter suggests the sequence studied with MD simulation may be more responsive to increases in temperature, compared to the one used in NMR, raising the possibility of increased native flexibility at 37°C. Notably, root-mean-square fluctuations calculated from the simulation agree reasonably with crystallographic B-factors extracted for HBV capsid crystal structures of the same sequence. (Hadden et al., 2018). as well as with extracted detector responses (Supplementary Figure S14).

The presence of alanine at S35, C48, C61, and C107 in the simulation model incurs the loss of hydrogen-bonding capability, thus eliminating stabilizing interactions; the shortening of the carboxylate side chains at E40 and E64 likewise alters the potential for salt bridges. A detailed examination of the simulation trajectory suggests that C48, which showed a notable disparity in the detector response, may be in proximity to interact with S44, E46, H47, S49, H52, T53, or R56 in the sequence studied by NMR, and the absence of such contacts may account for enhanced motion of the residue and its parent loop (43E-51H) connecting the $\alpha_{2}\;$ and $\alpha_{3}$ helices. C107 may be in proximity to interact with R112, E113, or T114, imparting stability to the loop connecting helices $\alpha_{4b}$ and $\alpha_{5}$ , which showed higher mobility in the simulation compared with NMR. M93, another source of disparity between the experimental and computational datasets, has a longer hydrophobic sidechain that may better pack into the core of the four-helix bundle than the valine of the simulation model. As residue 93 has been measured as the vertex of the Cp149 hinge domain (Zhao et al., 2020; Pérez-Segura et al., 2021), loss of stabilization at this position likely contributes to the heighted motion predicted by simulation along the interface of helices $\alpha_{4a}$ and $\alpha_{4b}$ . Greater compliance of the hinge could account for increased flexibility observed for the spikes, along with partial unfolding of helix $\alpha_{4b}$ in C and D chains, which is largely maintained from the PDB 2G33 initial structure. The absence of the hydroxyl group at S35 may account for increased mobility of residues 35S-52H in the simulation owing to the loss of a hydrogen bond, likely with R39. Substitutions at S35, E40, C48, and C107 may all relate to differences in the dynamics observed for the N-terminus, including residues 2D-14E.

## Discussion

The measured solid-state NMR observables ( $R_{1}$ , $R_{1\rho }$ ) allowed us to investigate distributions of motions centered at about 1 nanosecond and at hundreds of nanoseconds to microsecond timescales. The 1 μs MD simulation of the Cp149 HBV capsid enables extraction of a continuum of quantitative information on motions spread over all timescales contained within the length of the trajectory and, to some extent, beyond, however with rapidly increasing uncertainties (Supplementary Figure S21, S22). The continuous nature of the simulation data represents a significant advantage compared to NMR data, which allows the investigation of motion only over discrete windows of timescales, dictated by the experimental conditions. Experimental verification of a subset of residues is however crucial.

**FIGURE 5:**
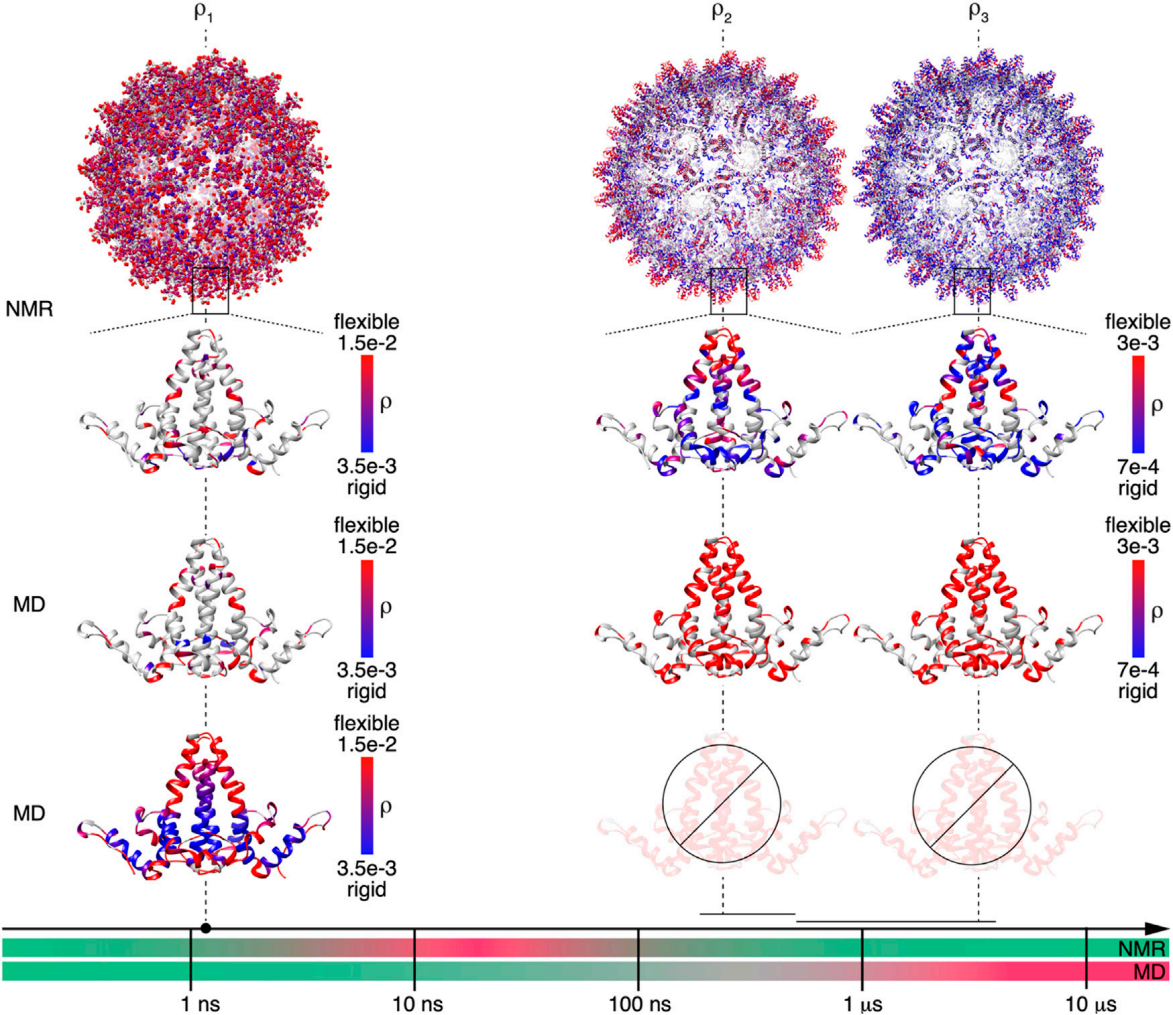
NMR and MD are complementary methods for characterizing Cp149 capsid dynamics up to the microsecond timescale. Comparison of the MD and NMR amplitude of motions $\rho_{1}^{\left(\text{θ},\text{ S}\right)}$ , $\rho_{2}^{\left(\text{θ},\text{ S}\right)}\;$ and $\;\rho_{3}^{\left(\text{θ},\text{ S}\right)}$ shown in a red to blue gradient on the Cp149 dimer structure (PDB 1QGT). The NMR data plotted on the full Cp149 capsid is shown on top (an intermediate scale representation is shown in Supplementary Figure S23). Residues not characterized by NMR are shown in grey. For easier comparison the first panel containing MD results (Hadden et al. 2018) only shows the color-coding for those residues that are also observed by NMR, while the second MD panel shows the predictions that can be made with MD for the dynamics of the full dimer structure. The timescale arrow at the bottom is color-coded according to the timescales accessible (green) and not accessible (red) by state-of the art  MD and NMR methods. The grey zone of MD corresponds to a timescale where the amplitudes of motion start showing increasing error bars as predicted by the MD error analysis presented in the Supplementary Information section S1. The barred dimer representations in the bottom right indicate that in these timescales it is not possible to obtain reliable data extrapolations by MD.

Detector responses on different timescales of motion were compared between NMR and MD simulation for the Cp149 capsid. The structures shown in Figure 5 graphically summarize and compare the dynamics found by NMR experiments and MD simulation for the intact capsid structure and a single Cp149 dimer, evaluated for the timescale windows accessible by the NMR experiment. While the dimer structures in the first row show the NMR results, the second row displays the MD simulation from (Hadden et al., 2018) for the NMR-visible residues only, and the third row shows the full information available from simulation.

On the nanosecond timescale (amplitudes of motion centered at 1.3 ns ), MD simulations (Hadden et al., 2018) and NMR experimental results agree well both on a qualitative and quantitative level, except for an overestimation of the loop motion (with the notable exception of 46E). As the residues which can be observed by NMR are relatively sparse, but their dynamics coincide well with those predicted by simulation, it is therefore justified to extrapolate motional information for all residues from the simulation results.

The faster of the 2 microsecond detectors $\rho_{2}$ is localized in the error-prone grey portion for the MD simulation. That the dynamics are not comparable can be clearly seen in Figure 5, where the NMR and simulation amplitudes of motion are depicted on the dimer structure in the third and fourth row, using the same color-bar scale. In this representation, the structure containing the simulation-derived data appears mainly red, whereas the NMR structure shows mixed colors. Indeed, the calculated high mobility for residues 35S-52H and around residues 92N/110F is not observed experimentally. It can be seen that the $\rho_{2}$ detector responses show clear differences, with large motions centered around the spike tip, and the residues at the inner lining of the capsid remaining rather rigid. One can note that these residues showed higher dynamics on the nanosecond timescale.

Finally, the comparison between  MD simulation and NMR for the slower microsecond amplitudes of motion ( $\rho_{3}$ , right panel) lying at the end of the trajectory reveals similar findings. The amplitudes of motion are mostly significantly larger in the MD simulation than NMR. The experimental data reveal that the spike residues show less dynamics than predicted on this timescale, which may be a consequence of the starting structure 2G33 where not all tips are well folded.

In summary, for short timescales, the two methods ideally provide identical results in the time-windows accessible to NMR. In this range, the NMR measurements validate the MD simulation results, which can then be used to characterize additional residues not resolved in the spectra, as well as additional timescales by interpolation/extrapolation. For slower timescales with correlation times approaching the length of the trajectory, the MD simulation becomes less reliable; the error increases significantly already around a timescale of 100 ns, approximately an order of magnitude smaller than the trajectory length (SI section S1). In the present study, the MD simulation data could not be used for extrapolations in timescale windows above 100 ns. In these windows, the predictions lose statistical viability, and in the absence of extended conformational sampling, access to experimental data becomes crucial.

The timescale axis shown in Figure 5 accounts for these observations by subdividing the timescales for the MD simulation predictions in three portions. The green one indicates motions that are well-described by the trajectory ( $\tau_{c}$ < 100 ns); the grey one where the prediction on the molecular motions starts being affected by larger uncertainties ( $100\;\text{ns}<\tau_{c}<1\;\text{μs}$ ); and the red one exceeding the length of the trajectory, where predictions are unreliable ( $\tau_{c}>1\;\text{μs}$ ) (see also Figure 4A panel (vi)).

## Conclusions

As predicted by a previous MD simulation study, the HBV capsid is not a rigid “tin can” but indeed shows considerable motion. These motions were detected by NMR on different timescales, from nanoseconds to microseconds. MD simulations and NMR both come to similar conclusions on the dynamics of HBV capsids for short timescales (nanoseconds), which experimentally validates the MD trajectory. Some loops were found to be significantly more rigid than predicted by MD simulation, however, this discrepancy may be, at least in part, attributed to differences in experimental conditions (e.g. temperature) and a 6% difference in sequence identity. The motional parameters for those residues not observed in the NMR spectra, e.g. because of signal overlap, can therefore be accurately estimated from the MD results. Predicting microsecond motion from the MD simulation trajectory is difficult because of insufficient statistics, and a rough error estimation indicates large uncertainties. For the microsecond detectors, the length of the MD trajectory is clearly too short for a good prediction, and for the slower two detectors, the experimental detector responses are more reliable. The accuracy and utility of the combined NMR/MD detector analysis is expected to increase as longer timescale MD simulations of large biomolecular assemblies become more accessible and common.

In summary, we found that for obtaining a full dynamic picture of HBV capsids, a combination of NMR and MD simulations approaches is a promising tool. Since on the faster timescales both methods come to similar results, MD simulations can be used to predict motions in the time window from 10 to 100 ns, where NMR is at the current state of technology has a blind spot. On the timescales from 100 ns- 10 μs , where the MD predictions are naturally limited by the length of the trajectory, solid-state NMR describes the overall dynamics much more reliably. Together, these complementary methods provide access to comprehensive characterization of the functional motions of large biomolecular assemblies, like HBV capsids.

## Data Availability

The original contributions presented in the study are included in the article/Supplementary Material, further inquiries can be directed to the corresponding authors.
