# Supplementary material for: Experimental Characterization of the Hepatitis B Virus Capsid Dynamics by Solid-State NMR
Source: Front Mol Biosci. 2022 Jan 3;8:807577. doi: 10.3389/fmolb.2021.807577 (PMC8762115; doi:10.3389/fmolb.2021.807577)
Supplement: Supplementary file 1 [file DataSheet1.pdf]

SUPPLEMENTARY INFORMATION FOR:

Experimental Characterization of the Hepatitis B Virus Capsid Dynamics by Solid-State NMR

Alexander A. Malär[1#], Morgane Callon[1#], Albert A. Smith[2], Shishan Wang[3], Lauriane Lecoq[3], Carolina Pérez-Segura[4], Jodi A. Hadden-Perilla[4], Anja Böckmann[3*] and Beat H. Meier[1*]

[1]Physical Chemistry, ETH Zürich, 8093 Zürich, Switzerland

[2]Departement of Medical Physics and Biophysics, Universität Leipzig, Härtelstraße 16-18, 04107 Leipzig, Germany

[3]Molecular Microbiology and Structural Biochemistry (MMSB) UMR 5086 CNRS/Université de Lyon, Labex Ecofect, 7 passage du Vercors, 69367 Lyon, France

[4]Department of Chemistry and Biochemistry, University of Delaware, Newark, Delaware 19716, United States

# These authors contributed equally

*Corresponding Authors: Anja Böckmann, a.bockmann@ibcp.fr; Beat H. Meier, beme@ethz.ch

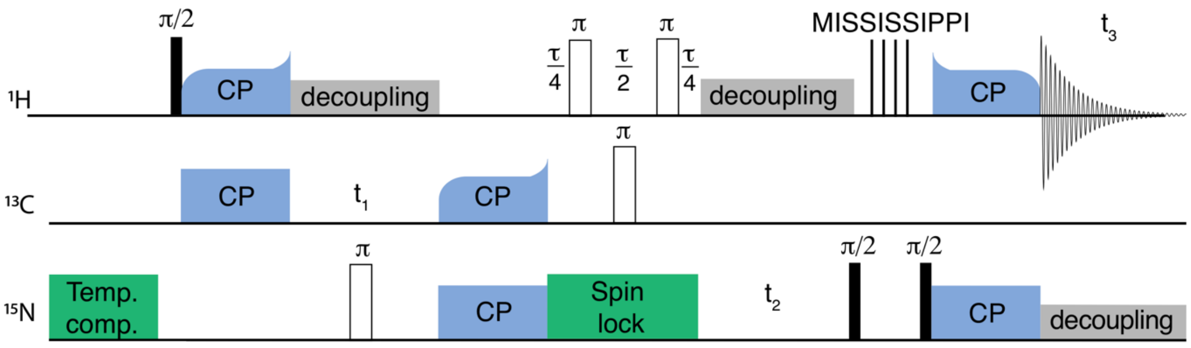

Fig. S1: hCANH-based pulse sequence for $R_{1\rho}(^{15}\mathrm{N})$ measurements in three-dimensional spectra. Due to the long spin-lock times required for ^{15}N relaxation measurements, a temperature compensation block is placed at the beginning of the sequence on the ^{15}N channel, to ensure that for each spin-lock duration, the same total power is deposited in the sample. The π pulses on ^{1}H applied at ¼ and ¾ of the ^{15}N spin-lock duration time are used to refocus a possible effect of ^{15}N CSA / H-N dipolar cross-correlated relaxation.

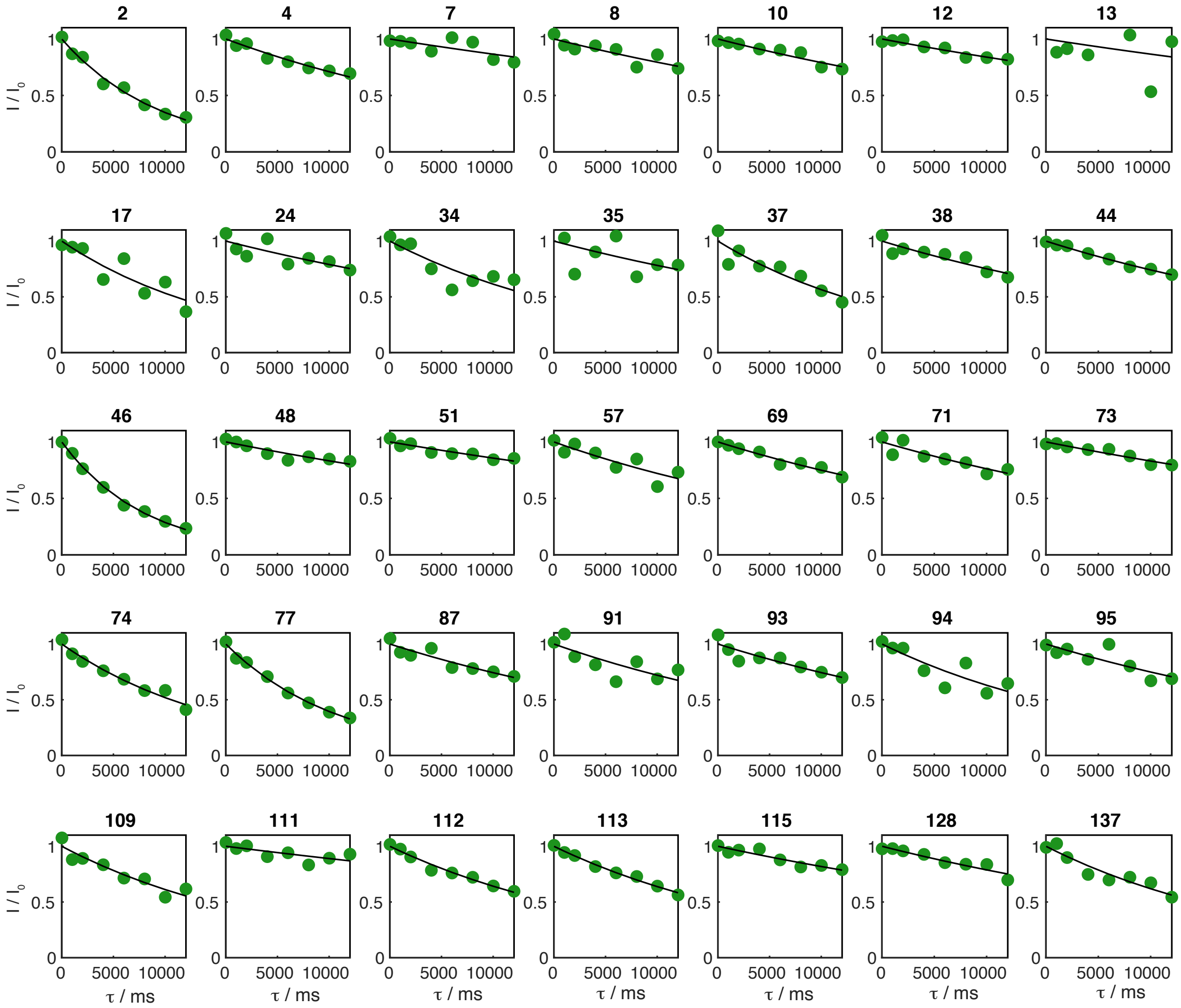

Fig. S2: Site-specific $T_1(^{15}N)$ relaxation traces, extracted from 2D hNH spectra recorded at 20.0 T external magnetic field, 110 kHz MAS (0.7mm rotor) with corresponding mono-exponential fits.

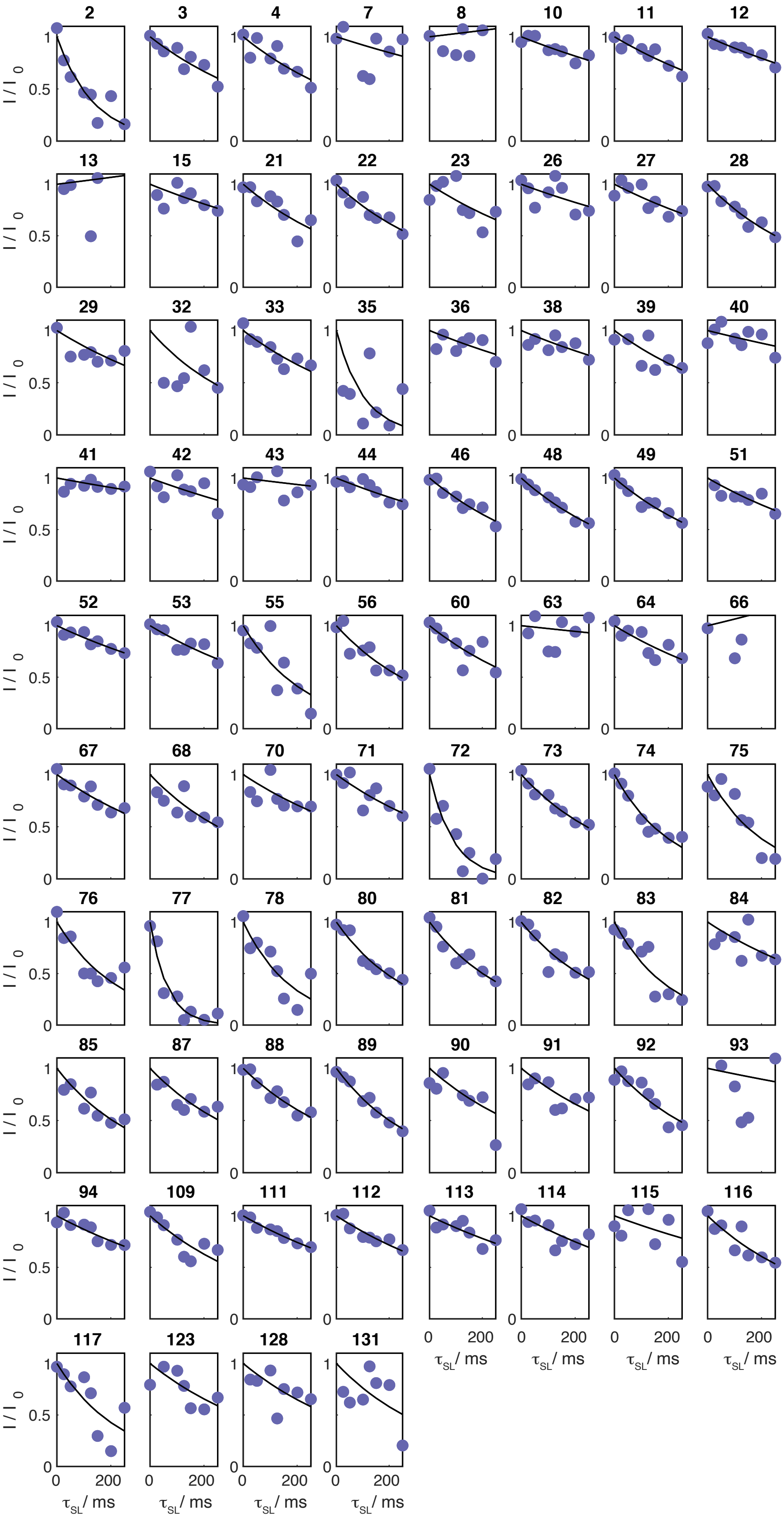

Fig. S3: Site-specific $T_{1\rho}(^{15}\text{N})$ relaxation traces, extracted from 3D hCANH spectra recorded at 20.0 T external magnetic field, 110 kHz MAS (0.7mm rotor) with corresponding mono-exponential fits.

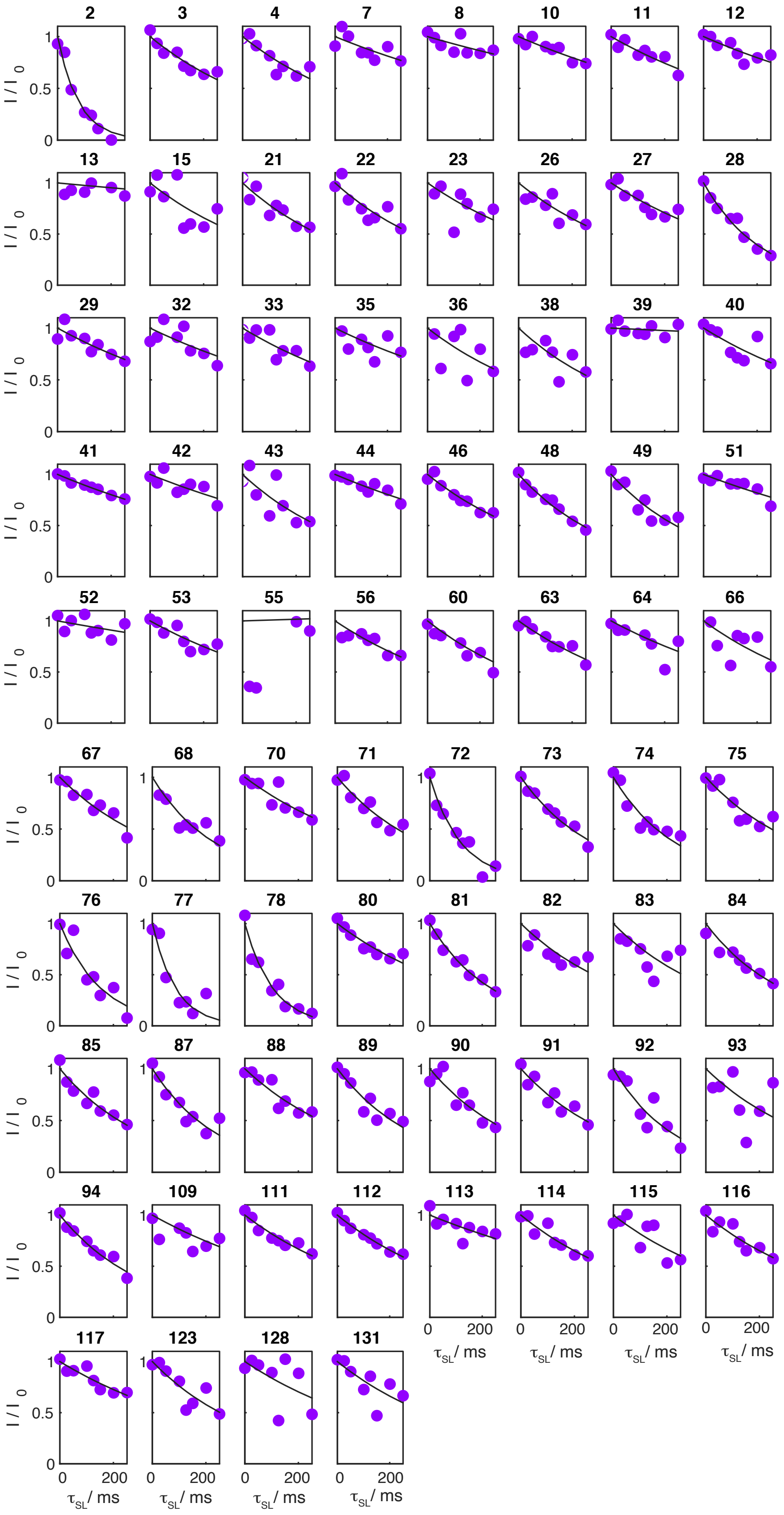

Fig. S4: Site-specific $T_{1\rho}(^{15}\mathrm{N})$ relaxation traces, extracted from 3D hCANH spectra recorded at 20.0 T external magnetic field, 80 kHz MAS (0.7mm rotor) with corresponding mono-exponential fits.

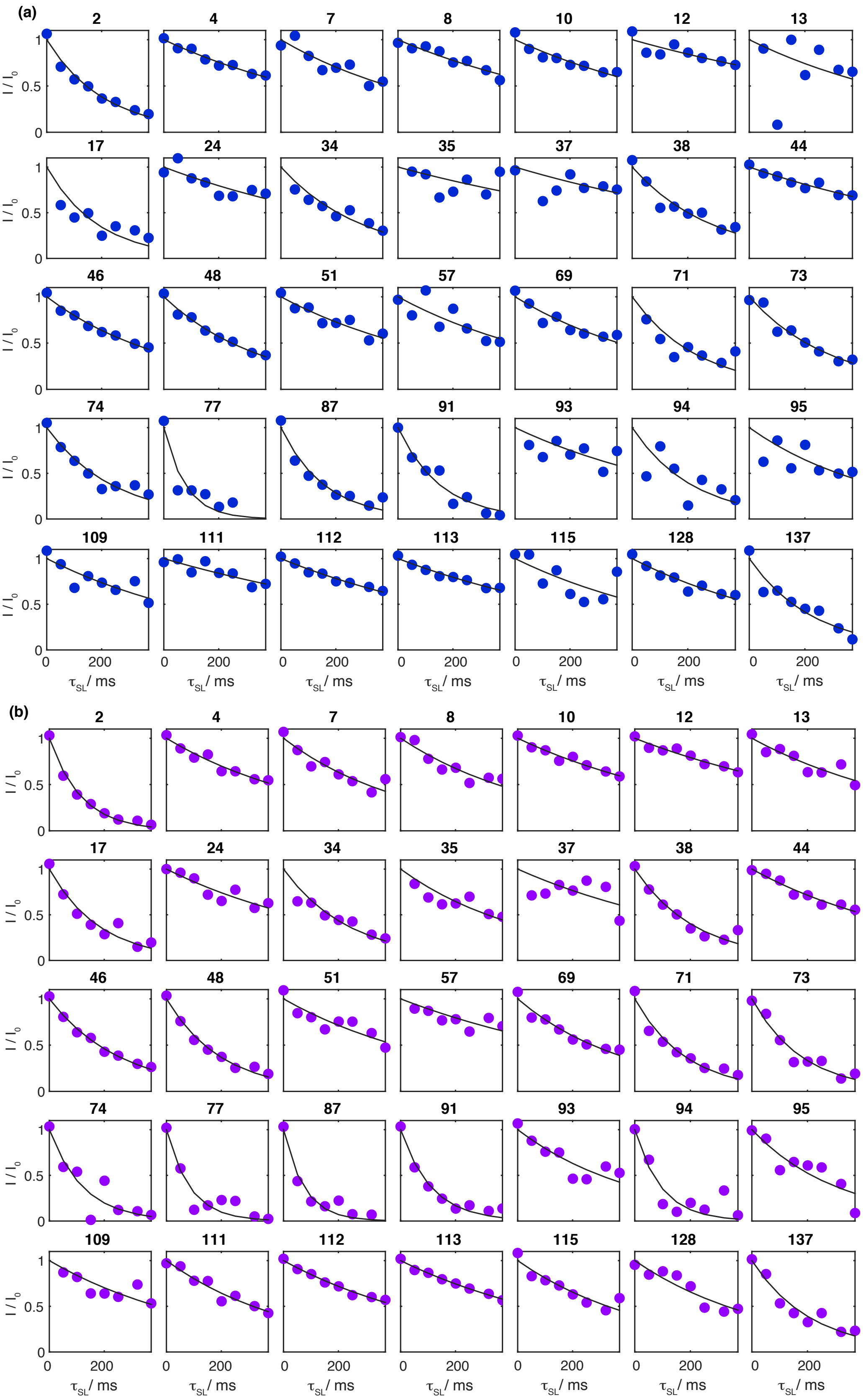

Fig. S5: Site-specific $T_{1\rho}(^{15}\mathrm{N})$ relaxation traces, extracted from 2D hNH spectra recorded at 20.0 T external magnetic field and (a) 160 kHz MAS, (b) 80 kHz MAS (0.5mm rotor) with corresponding mono-exponential fits.

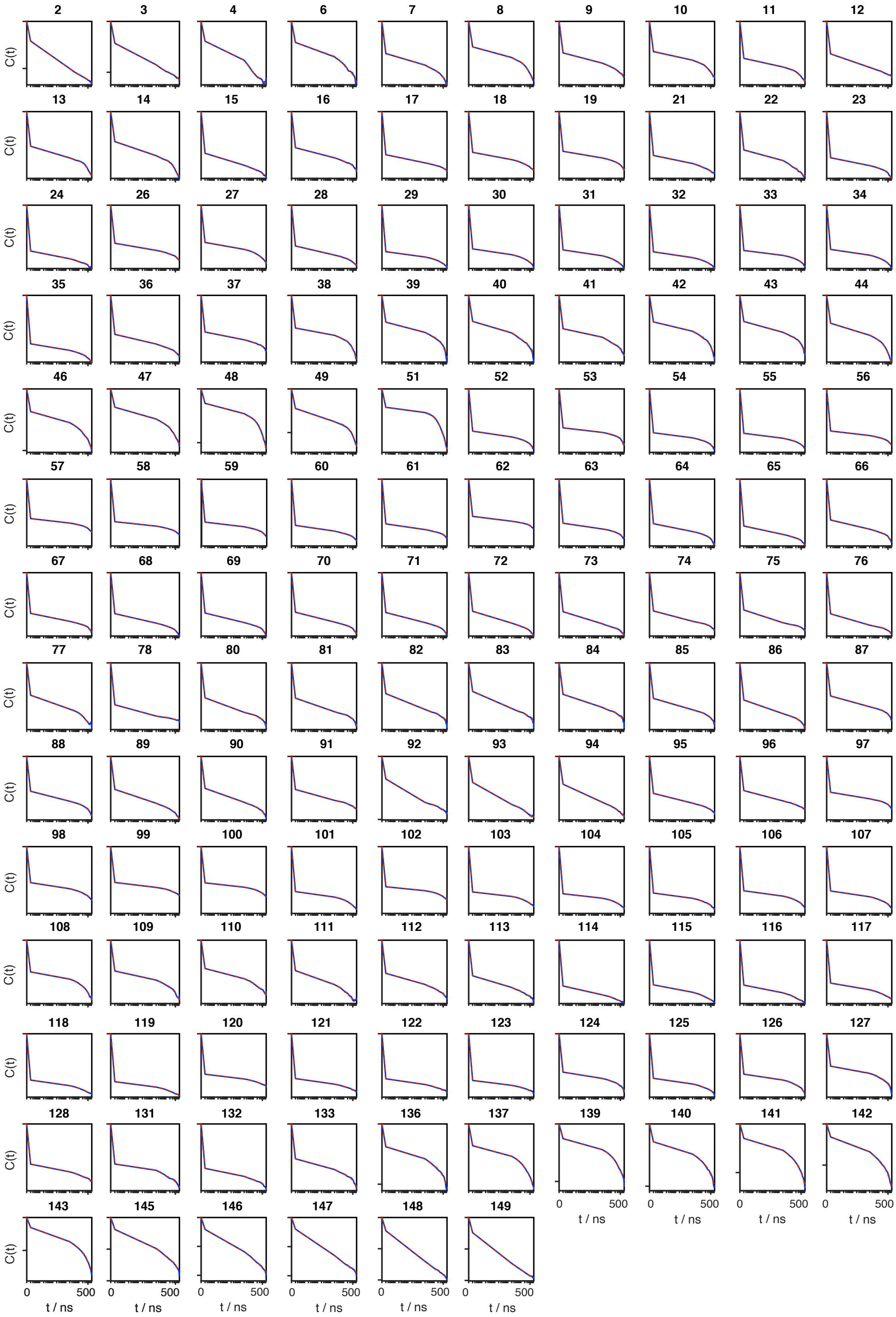

Fig. S6: Residue-specific correlation functions $C(t)$ extracted from the MD trajectory for chain A (blue) and best fit, obtained solving the inverse Laplace transform problem with $\lambda = 0$ (red).

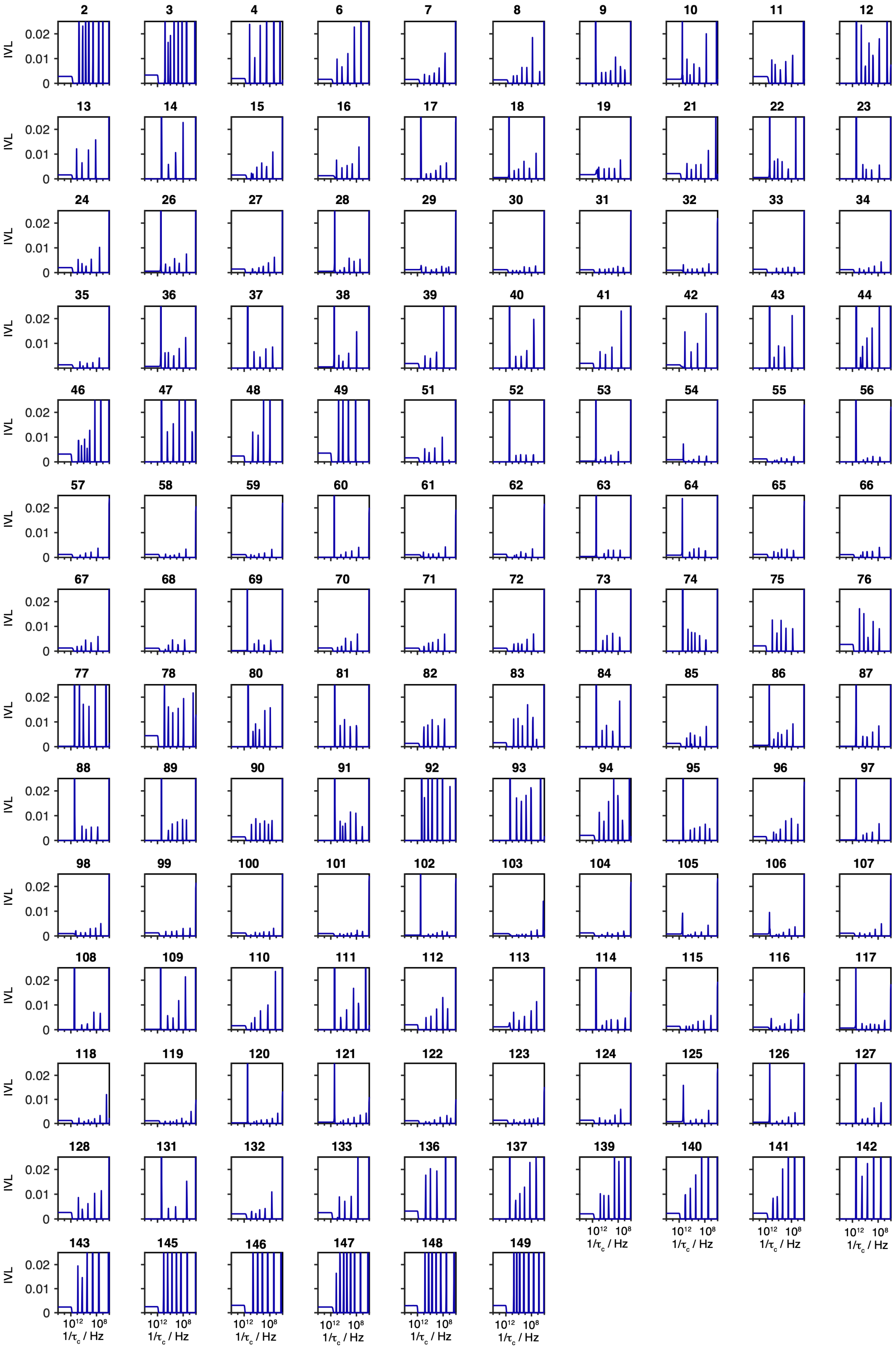

Fig. S7: Residue-specific inverse Laplace transforms (IVL) obtained from the correlation functions of the MD trajectory for chain A and a regularization weight of $\lambda = 0$. The IVL was performed with $\tau_c = [10^{-14}, 10^{-6}\ ]s$.

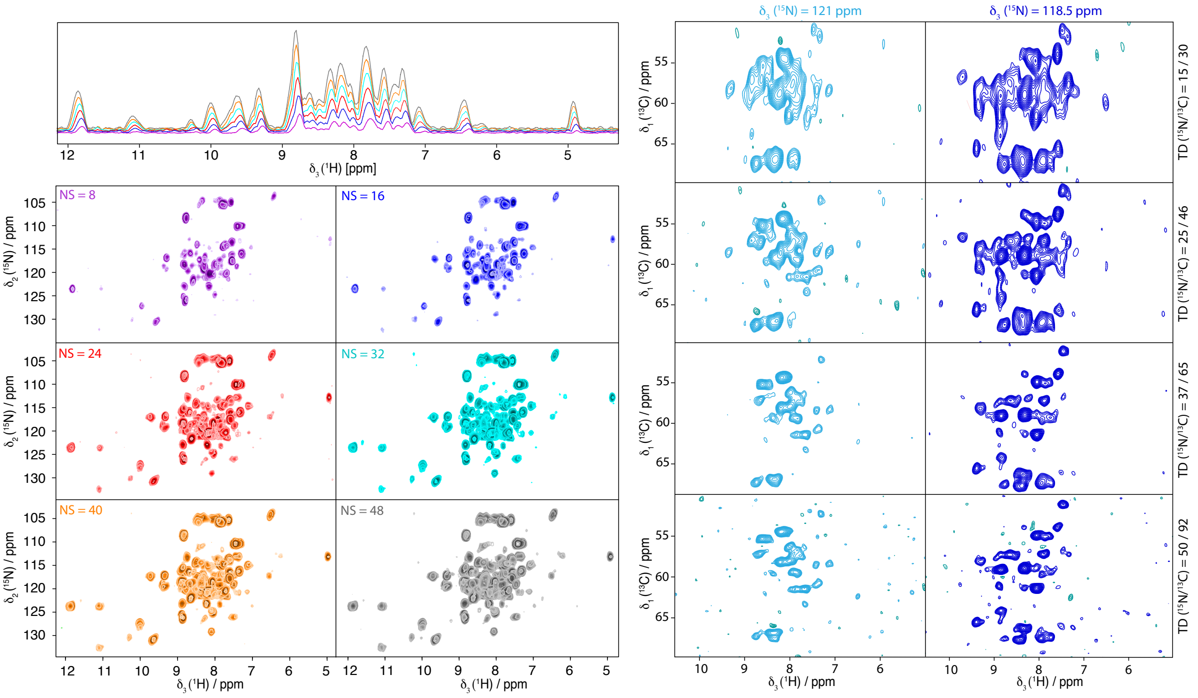

Fig. S8: Illustration for a comparison of number of scans and spectral resolution compromises in 3D hCANH spectra of Cp149 recorded at 110 kHz MAS and 20.0 T external magnetic field, used to reduce the experimental time of one 3D experiment to a maximum of one day of measurement time. In the final experiment used for the $T_{1\rho}(^{15}\mathrm{N})$ measurements, 24 scans and 30/50 points in the indirect dimensions were used.

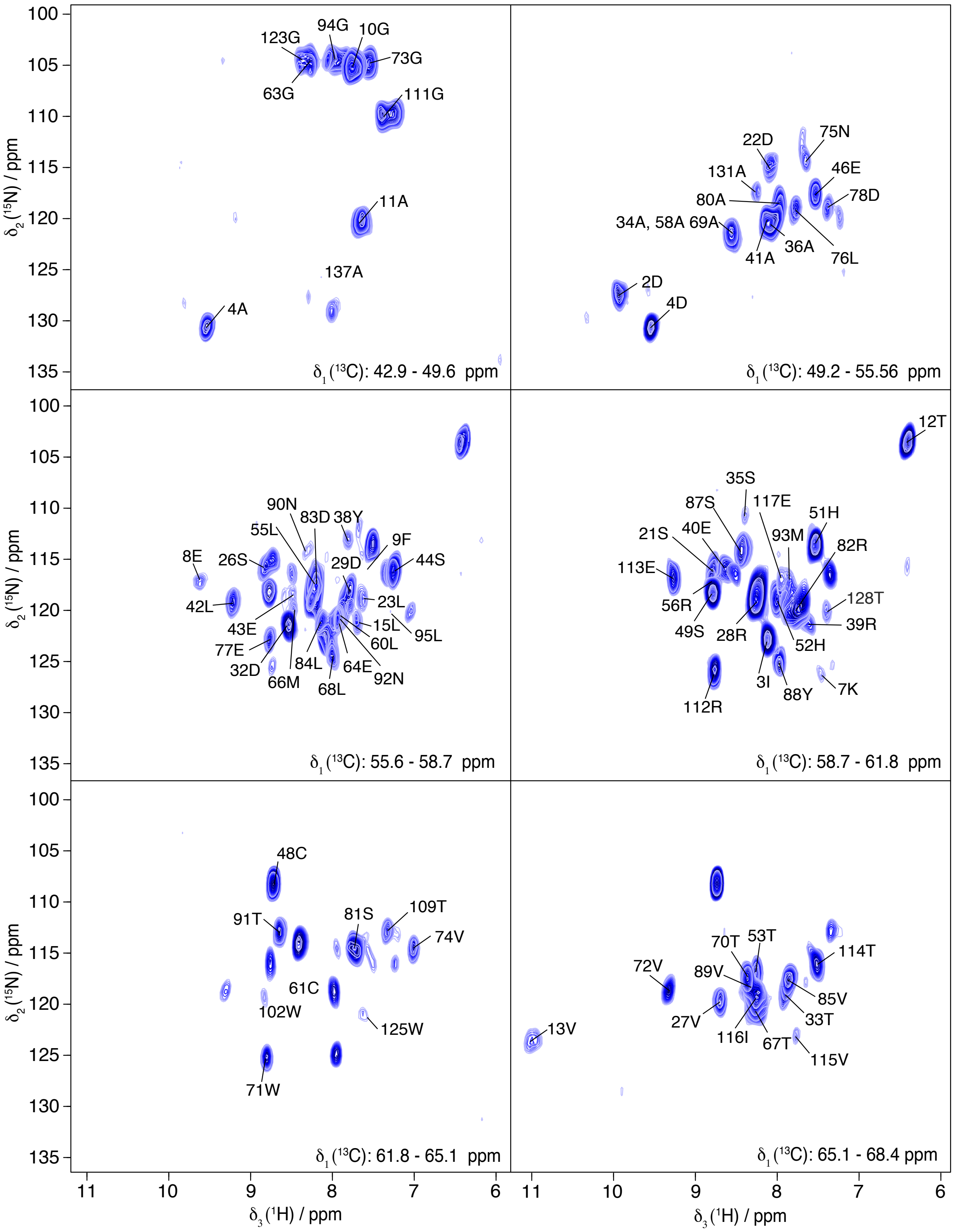

Fig. S9: HN slices for different regions of ^{13}C chemical shifts of the 3D hCANH experiment recorded at 110 kHz MAS and 20.0 T external magnetic fields, with 24 scans and 30/50 points in the indirect dimension.

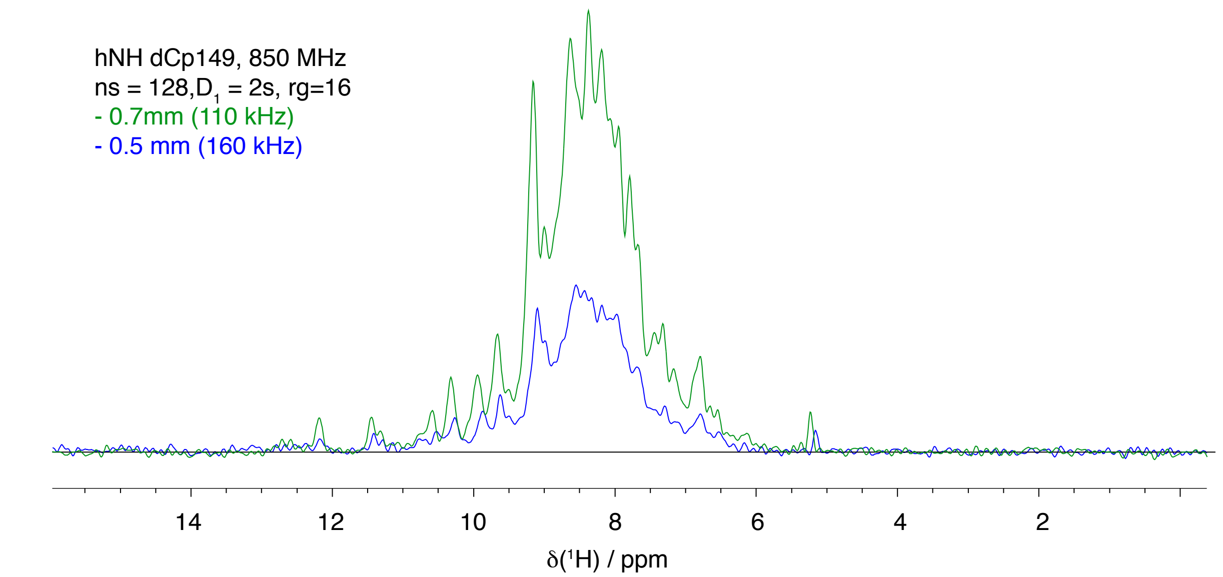

Fig. S10: Overlay of first free-induction decay (FID) of hNH experiments recorded at 110 kHz in an 0.7mm rotor (green) and 160 kHz in an 0.5 mm rotor (blue) at 20.0 T external magnetic field, with 128 scans, a receiver gain (RG) of 16 and 2s of inter-scan delay.

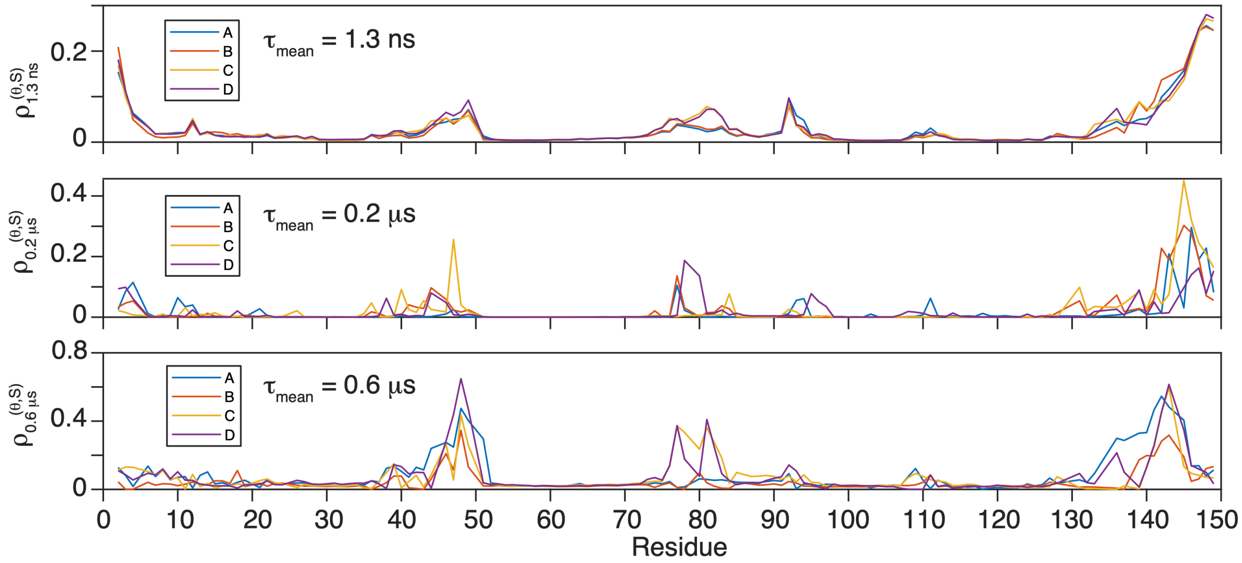

Fig. S11: Molecular dynamic detector responses of the four capsid subunits A,B,C,D for the $\rho_{1.}, \rho_2, \rho_3$ detectors shown in Fig.3b, constructed from the rotating-frame relaxation-rate constants at 13 kHz spin-lock strength and 110 kHz and 80 kHz MAS and the longitudinal relaxation rate constant at 850 MHz. MD detector optimization has been carried out over the correlation time interval $\tau_c = [10^{-14}, 10^{-6}\,]s$.

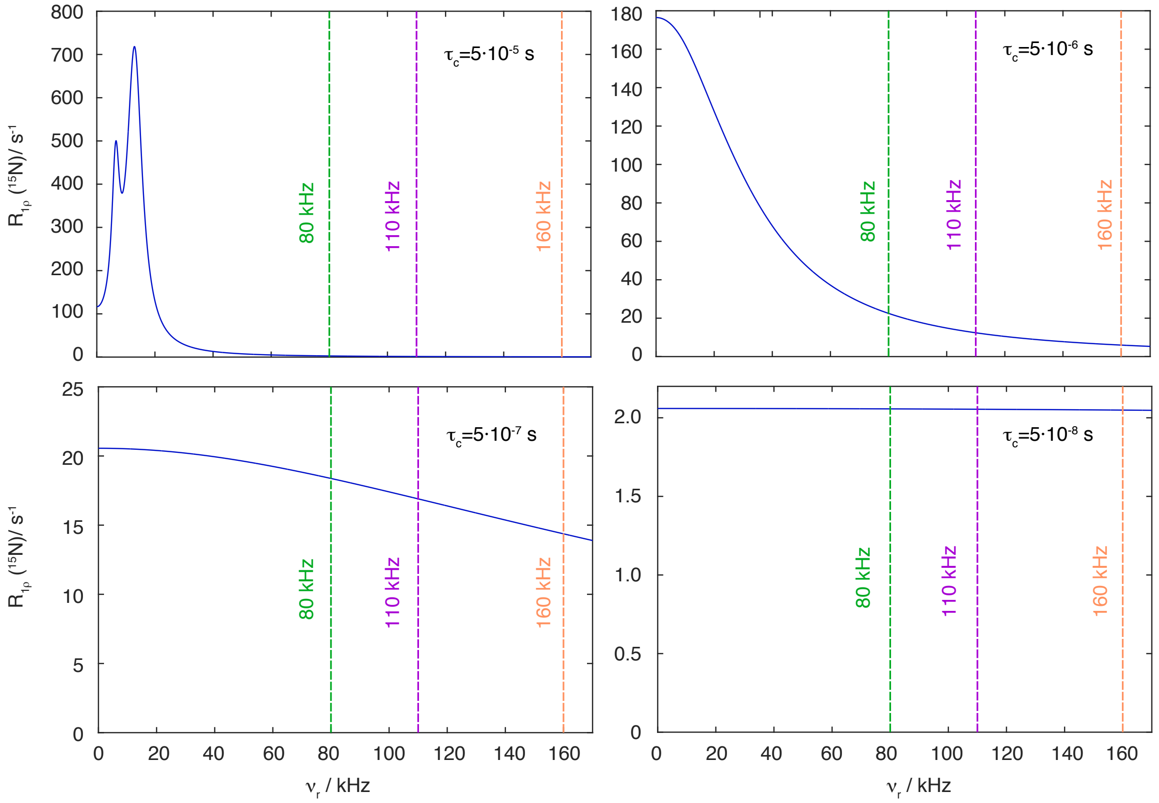

Fig. S12: Analytical form of the $R_{1\rho}(^{15}\mathrm{N})$ relaxation-rate constant for 13 kHz spin-lock strength as function of the MAS frequency and different correlation times ranging from $5 \cdot 10^{-5}$s to $5 \cdot 10^{-8}$s. The vertical dashed lines correspond to the used experimental conditions (80, 110 and 160 kHz MAS).

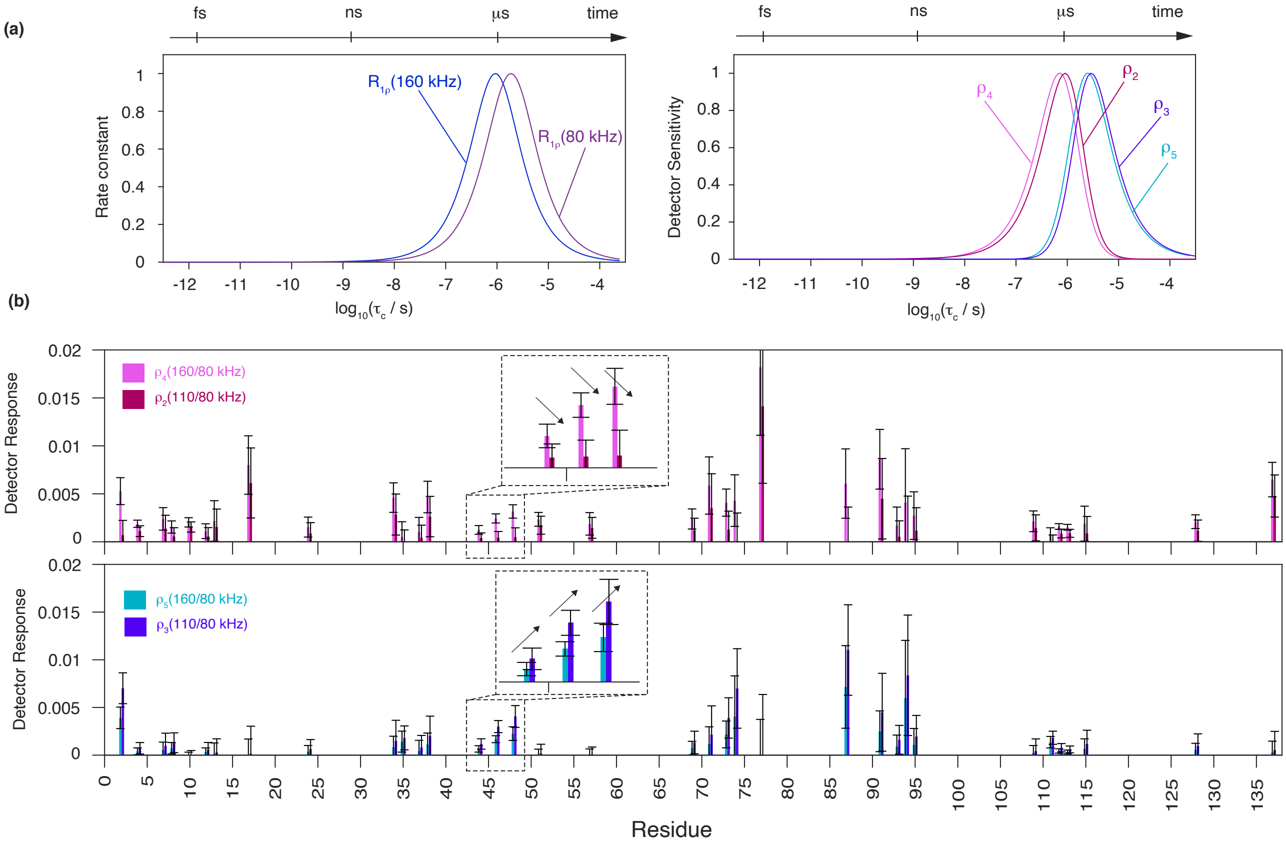

Fig. S13: (b) Analysis of the experimental rotating-frame relaxation rate constants recorded at 160/80 kHz, using $\rho_2^{(\theta,S)}$ $\rho_3^{(\theta,S)}$ and $\rho_4^{(\theta,S)}$ $\rho_5^{(\theta,S)}$ detector sensitivities depicted in (a).

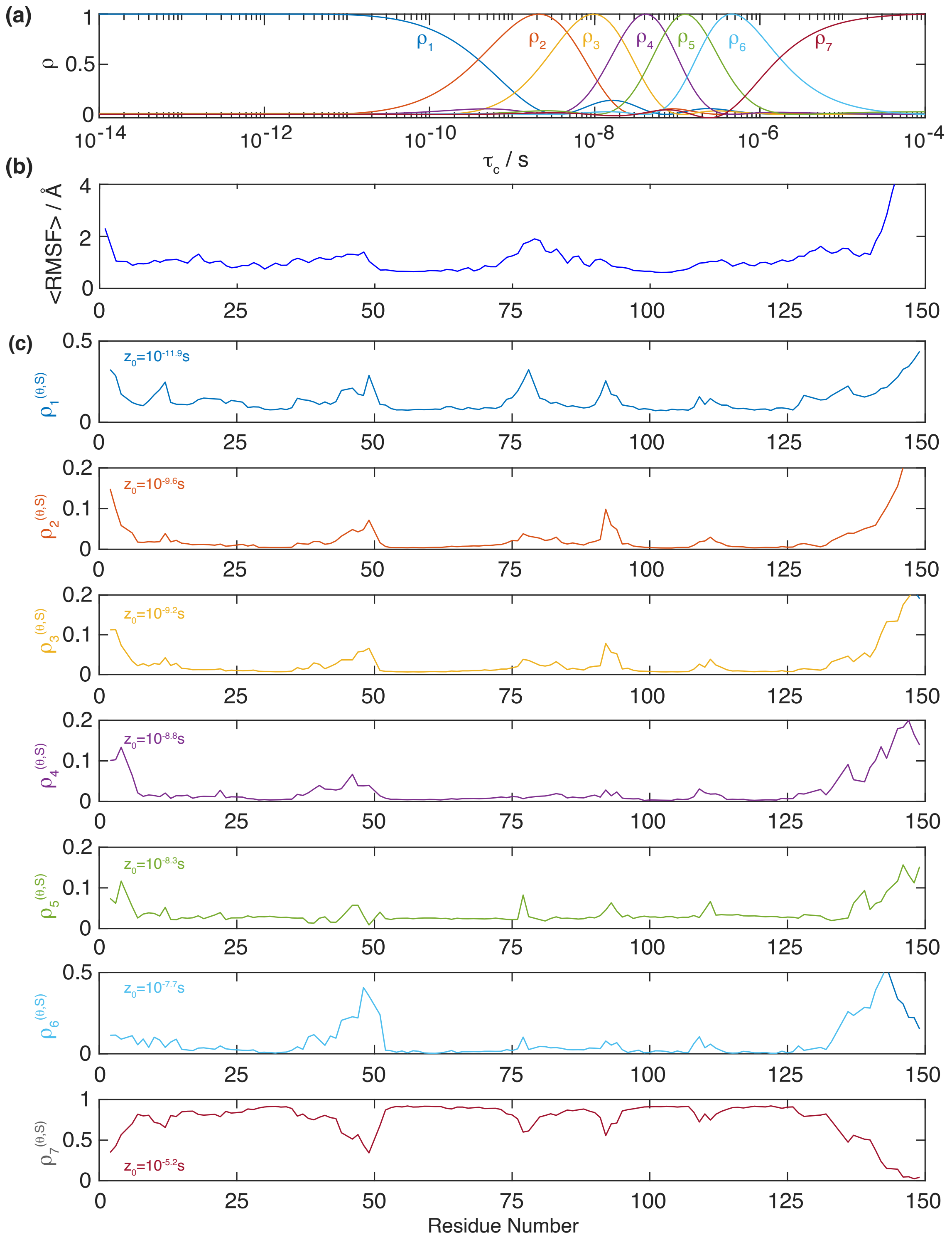

Fig. S14: (a) Detector sensitivities $\rho_1, \ldots, \rho_7$ positioned at each order of magnitude on the correlation time axis. (b) root-mean-square fluctuations (RMFS) calculated for a A-B dimer in reference *(Hadden et al. 2018)* from main text. (c) Corresponding averaged detector responses extracted from the molecular dynamics trajectory for $\rho_1, \ldots, \rho_7$ with mean correlation times ranging from $10^{-11.9}$s to $10^{-5.2}$s. Inverse Laplace transforms and detector profile optimization have been performed over the correlation time interval $\tau_c = [10^{-14}, 10^{-6}\,]s$.

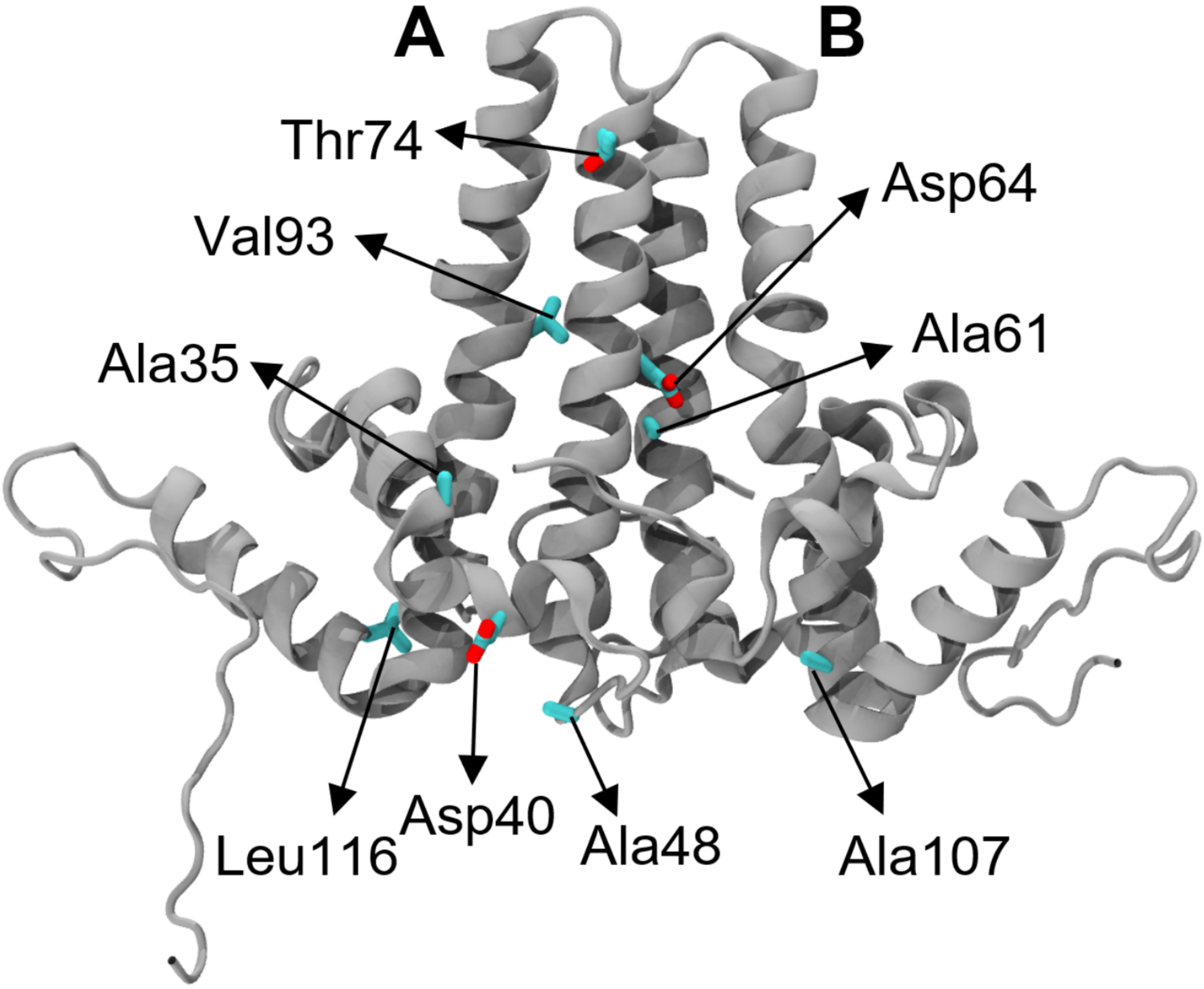

Fig. S15: The simulation model of mutated Cp149 and the wild-type sequence analysed with NMR in the present work differ by nine amino acid residues: A35/S35, D40/E40, A48/C48, A61/C61, D64/E64, T74/V74, V93/M93, A107/C107, and L116/I116. The location of each amino acid substitution is indicated on the Cp149 structure.

Table S1: Experimental tables for solid-state NMR acquisition and pulse program parameters used in the relaxation experiments.

**Experiment**	**hCANH 3D, $T_{1\rho}$(^{15}N), 110 kHz (DUL Cp149)**	**hCANH 3D $T_{1\rho}$(^{15}N), 80 kHz (DUL Cp149)**
MAS frequency/ kHz	110	80
Field/ T	20	20
Transfer I	HC-CP(DQ)	HC-CP(DQ)
^{1}H field/ kHz	88	61
^{13}C field/ kHz	15	15
Shape	Tangent ^{1}H	Tangent ^{1}H
Carrier / ppm	51	52
Time/ ms	4.5	4.5
Transfer II	CN-CP	CN-CP

^{13}C field/ kHz	68	50
^{15}N field/ kHz	40	29
Shape	Tangent ^{13}C	Tangent ^{13}C
Carrier/ ppm	117.5	117.5
Time/ ms	16.0	17.0
Transfer III	NH-CP	NH-CP
^{1}H field/ kHz	83	59
^{15}N field/ kHz	16	17
Shape	Tangent ^{1}H	Tangent ^{1}H
Carrier/ ppm	4.8	4.8
Time/ ms	2.7	2.4
**$T_{1\rho}$(^{15}N) Measurement**	13 kHz Spin-Lock ^{15}N	13 kHz Spin-Lock ^{15}N
Relaxation delays / ms	0.001, 26, 51, 101, 126, 151, 201, 251	0.001, 26, 51, 101, 126, 151, 201, 251
t1 increments	50	50
Sweep width (t1)/ ppm	30	30
Acquisition time (t1)/ ms	3.9	3.9
t2 increments	30	30
Sweep width (t2)/ ppm	40	40
Acquisition time (t2)/ ms	4.4	4.4
t3 increments	2048	2048
Sweep width (t3)/ ppm	47	47
Acquisition time (t3)/ ms	25.8	25.8
^{1}H swfTPPM decoupling/ kHz	10	10
^{15}N WALTZ64 decoupling/ kHz	10	5
^{13}C WALTZ64 decoupling/ kHz	5	5
Water Suppression	MISSISIPPI	MISSISIPPI
^{1}H field / kHz	20	20
Time / ms	120	120
Interscan delay/ s	2.19	2.19
Number of scans	24	24
Measurement time/ h	184	184

**Experiment**	**hNH 2D $T_{1\rho}$(^{15}N) 160 kHz (DUL Cp149)**	**hNH 2D $T_{1\rho}$(^{15}N) 80 kHz (DUL Cp149)**
MAS frequency/ kHz	160	80
Field/ T	20	20
**Transfer I**	HN-CP (DQ)	HN-CP (DQ)
^{1}H field/ kHz	118	63
^{15}N field/ kHz	30	15
Shape	Tangent ^{1}H	Tangent ^{1}H
Time / ms	1.1	1.4
**Transfer II**	NH-CP (DQ)	NH-CP (DQ)
^{1}H field/ kHz	118	63

^{15}N field/ kHz	30	15
Shape	Tangent ^{1}H	Tangent ^{1}H
Time / ms	1.2	1
**$T_{1\rho}$(^{15}N) Measurement**	13 kHz Spin-Lock ^{15}N	13 kHz Spin-Lock ^{15}N
Relaxation delays / ms	0.001, 51, 101, 151, 201, 251, 321, 371	0.001, 51, 101, 151, 201, 251, 321, 371
^{1}H carrier/ ppm	4.7	4.7
^{15}N carrier/ ppm	107	107
t1 increments	324	324
Sweep width (t1)/ ppm	70	70
Acquisition time (t1)/ ms	26.8	26.8
t2 increments	2048	2048
Sweep width (t2)/ ppm	40	40
Acquisition time (t2)/ ms	30.0	30.0
Water Suppression	MISSISIPPI	MISSISIPPI
^{1}H field / kHz	5	5
Time / ms	30	30
^{1}H swfttpm decoupling power/ kHz	5	5
^{15}N WALTZ64 decoupling power/ kHz	10	10
Interscan delay/ s	2	2
Number of scans	32	32
Measurement time/ h	50	50

**Experiment**	**CP-hNH T_1'(^{15}N) (DUL Cp149)**
MAS frequency/ kHz	108
Field/ T	20
**Transfer I**	HN-CP (DQ)
^{1}H field/ kHz	86
^{15}N field/ kHz	17
Shape	Tangent ^{1}H
Time / ms	1.3
**Transfer II**	NH-CP (DQ)
^{1}H field/ kHz	86
^{15}N field/ kHz	17
Shape	Tangent ^{1}H
Time / ms	1.3
**T_1(^{15}N) Measurement**	z storage on ^{15}N
Relaxation delays / s	0.02 1.02 2.02 4.02 6.02 8.02 10.02 12.02
^{1}H carrier/ ppm	4.7
^{15}N carrier/ ppm	107
t1 increments	160
Sweep width (t1)/ ppm	55
Acquisition time (t1)/ ms	16.9
t2 increments	2048
Sweep width (t2)/ ppm	40.1
Acquisition time (t2)/ ms	30.0
Water Suppression	MISSISIPPI
^{1}H field / kHz	10
Time / ms	30
^{1}H swfttpm decoupling power/ kHz	10
^{15}N WALTZ64 decoupling power/ kHz	10
Interscan delay/ s	1.2
Number of scans	16
Measurement time/ h	38

Section S1: Error Analysis on the detector responses obtained from the molecular dynamic trajectory.

In this section we show the considerations that have been used to construct the estimated error on the quantitative MD prediction of amplitudes of motions at different timescales shown in the main text Fig. 3 (grey shaded area). We will discuss in detail two contributions leading to uncertainty, when trying to extract quantitative predictions on motions from the $1\,\mu s$ MD trajectory and will illustrate how both errors grow significantly for timescales approaching the end of the trajectory in particular for the detectors constructed from the experimental conditions over a correlation time rage from $[10^{-14}, 10^{-6}]$ s. Doing the error investigation on a detector analysis that uses a set of seven almost non-overlapping detector sensitivities spaced at each order of magnitude (with mean correlation times ranging from $10^{-11.6}$s to $10^{-5.0}$s) allows for monitoring the increase of the error sources for slower timescales and thereby proposing the position of the boundary between the green and grey zones of the MD prediction, discussed in the main text. The whole analysis has been performed on chain A of the Cp149 capsid.

S1.1 Detector Responses for detectors approaching the end of the trajectory are less well defined (influence of the regularization parameter)

As sketched in the main text Fig.3(a) amplitudes of motion at all timescales are extracted from the correlation function of the $1\,\mu s$ MD trajectory by computing an inverse Laplace transform. This is mathematically an ill-posed problem, solved with the help of a regularization function, consisting in a minimization of the second derivative of the distribution of motion Θ. The relative priority of minimizing the second derivative, compared to obtaining a good fit of the input correlation function is determined by the regularization parameter λ. Increasing the regularization parameter will in general lead to a smoother/broader distribution of motion. If the regularization parameter is increased too much, the obtained distribution of motion will broaden to such an extent that it will not lead to a good fit of the input correlation function anymore. This behaviour of the total fit error on the regularization parameter translates into an L-shaped curve typical of regularization problems. The L-curve for the dependence of the total fit error as function of the regularization weight in chain A of Cp149 is shown in Fig.S16(a). In the zoom given in the insert of Fig.S16(a) it can be seen that up to around $\lambda = 10^5$, the total fit error increases less than 50% of the initial error at $\lambda = 0$, while it starts growing substantially afterwards. Fig. S16(b) shows the expected broadening of the distribution of motion with increasing the regularization parameter ($\lambda = 0, 10^3, 10^5$). It has been shown by (Smith et al. 2019) on HET-s(218-289) that this change in the form of the distribution of motion has in general only a minor impact on the extracted detector responses. Finding the distributions of motion is an ill-posed problem, but the step from the distributions of motion to the actual detector responses is such, that the result of the two-step process is a rather well-defined problem. Indeed, the detector sensitivities are convoluted with the distribution of motion and serve as a regularization function themselves, being much broader

than the individual peaks in the distribution of motion (green, pink and blue areas in Fig.S16(b)).

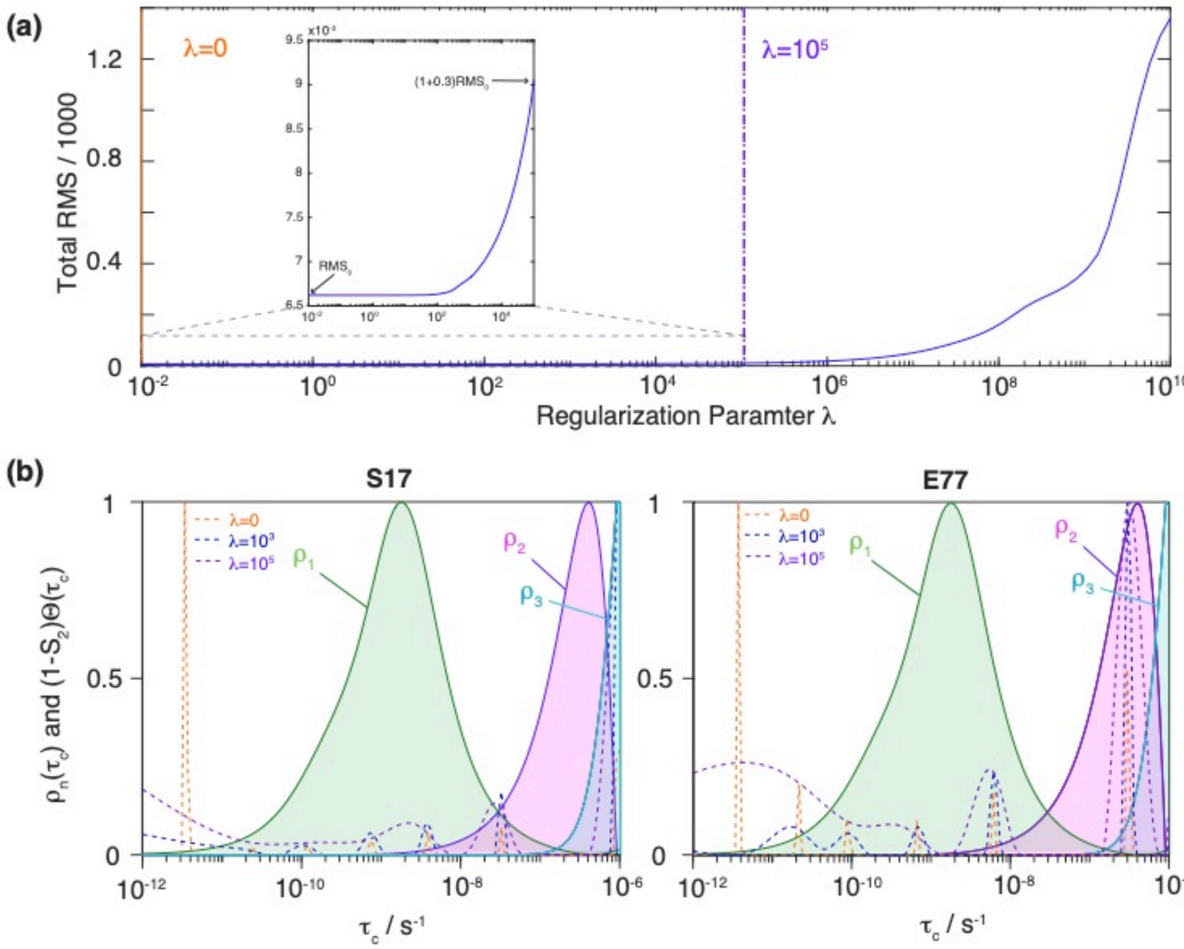

Fig. S16: *Influence of the regularization parameter on the distributions of motion*. **(a)** Total fit error between MD correlation function and correlation function obtained by solving the inverse Laplace problem as function of the regularization parameter λ. **(b)** Illustration of the effect of the regularization parameter on the distributions of motion (1-S^2)Θ of two different residues for $\lambda = 0, 10^3, 10^5$. The coloured green, pink and blue areas correspond to the sensitivities ρ_n of the experimental detector sensitivities shown in the main text Fig.3(a), which are convoluted with the distribution of motion to obtain the detector responses.

However, motions at long correlation times, approaching the end of the trajectory can be indistinguishable from each other based on the time points available in the correlation function. This can lead to ill-defined detector responses introducing a dependence of the detector responses to the regularization function. Thus, a variety of slower motions can be fitted equally well, because the trajectory is not long enough and does not contain sufficient points to define a clear peak position. Fig.S16(b)) illustrates how the systematic broadening of the peaks in the distribution of motion due to an increase in regularization parameter can lead to an additional overlap with a neighbouring detector and thereby alter the detector responses, as it is particularly evident for E77. This is a possible source of error when describing slow motions. Note that this effect does not come from a bad fitting of the input

correlation function. All chosen λ values correspond to regularization weights for which the total RMS of the fit is small (see Fig.S16(a)).
This effect was systematically investigated over a large range of timescales, by using the seven detector sensitivities spaced at each order of magnitude shown in Fig.S17(a) together with distribution of motions obtained with regularization parameters between $\lambda = 0$ and $\lambda = 10^5$ (chosen such that the RMS increases less than 50% of its initial value for $\lambda = 0$). We find that the curves are almost indistinguishable from each other at all timescales. Note however that this finding is also influenced by the choice and number of detectors used for the analysis. The seven detectors given in Fig.S17(a) are specifically optimized over the full correlation time range $[10^{-14}, 10^{-4}]$ s based on the available data (the $1\ \mu s$ trajectory), such that it generates broad detectors where there is uncertainty and narrow detectors where there is more confidence. The situation is different when performing the regularization influence analysis specifically for the three detectors optimized from the experimental conditions and on the restricted correlation time range from $[10^{-14}, 10^{-6}]$ s (Fig.S18). Indeed, we now observe a significant increase in regularization error at the slower timescales, which is likely due to the narrowness of the $\rho_{0.6\mu s}$ detector, which is restricted by the ending of the MD trajectory.

S1.2 Motions approaching the longest correlation times are not equilibrated

The MD trajectory contains 60 copies of the Cp149 molecule. This allows to calculate the correlation function and inverse Laplace transform for all 60 copies and use it to obtain the corresponding detector responses for the set of seven detector sensitivities spaced at each order of magnitude (shown in Fig.S17(a)). The site-specific standard deviations over the different detector responses for all 60 copies could thus be calculated and compared between the different orders of magnitude. For better comparison the squared standard deviation (variance) was normalized by the mean over all 60 copies (σ^2/mean) in Fig.S19, accounting for the amplitude of each motion. The variance was divided by the mean, instead of the standard deviation directly, since if one assumes an underlying Poisson statistic, the variance equals the expectation value for the mean.
We observe, as expect, the total error to be smaller for fast motions and higher for slow motions approaching the end of the trajectory, due to the slower motions not being well equilibrated in the given trajectory. The only exception represents the detector centred at 10^{-5}s, which is however special because it describes motions outside of the actual trajectory length, which are therefore purely extrapolated by the $1\ \mu s$ trajectory.

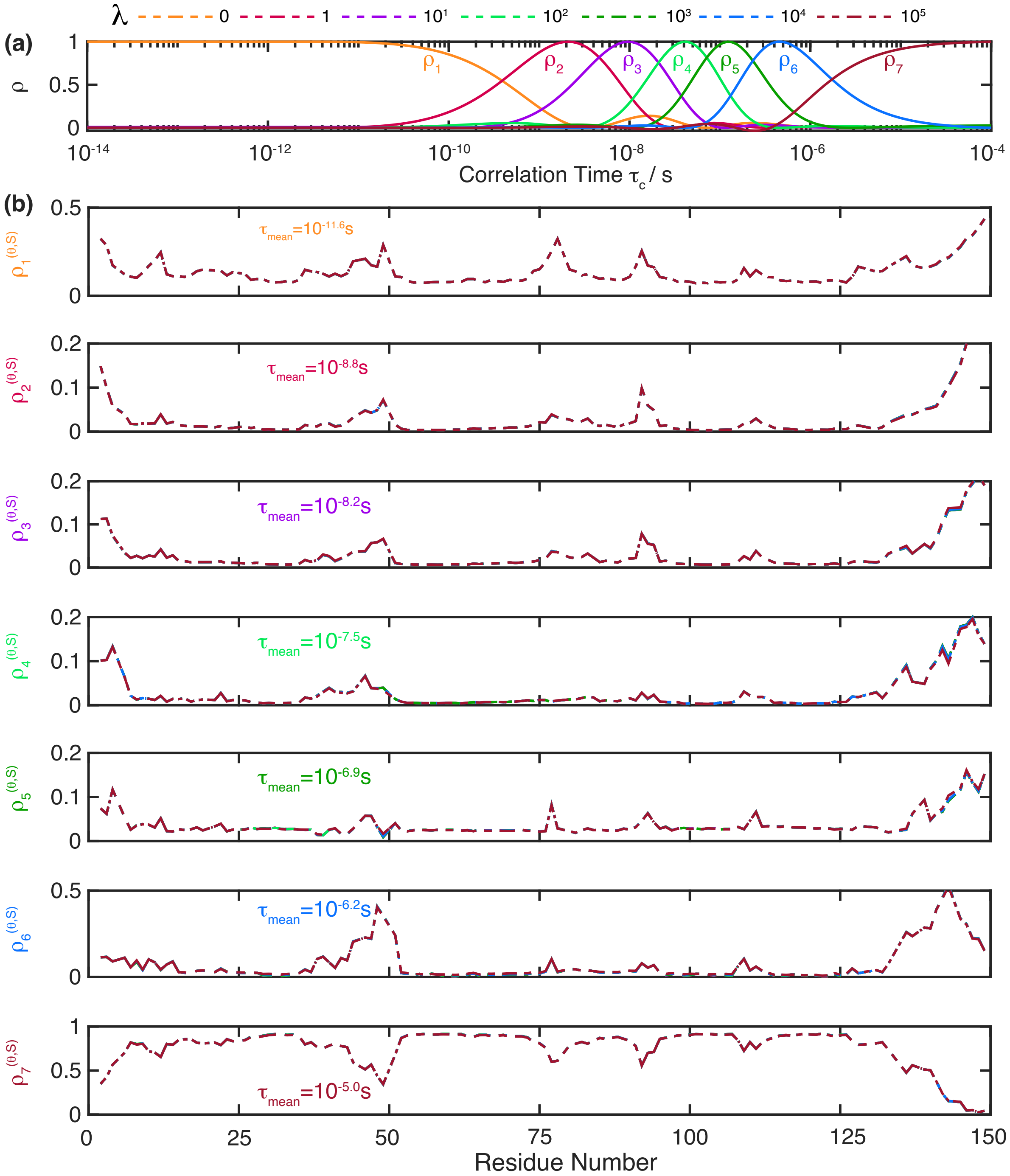

Fig. S17: *Influence of the regularization parameter on detector responses*. **(a)** Detector sensitivities spaced at each order of magnitude. **(b)** Detector responses for the detectors spaced at each order of magnitude from (d), using the inverse Laplace transforms obtained with a regularization parameters from $\lambda = 0$ to 10^5. Inverse Laplace transforms and detector profile optimization have been performed over the correlation time interval $\tau_c = [10^{-14}, 10^{-4}]s$.

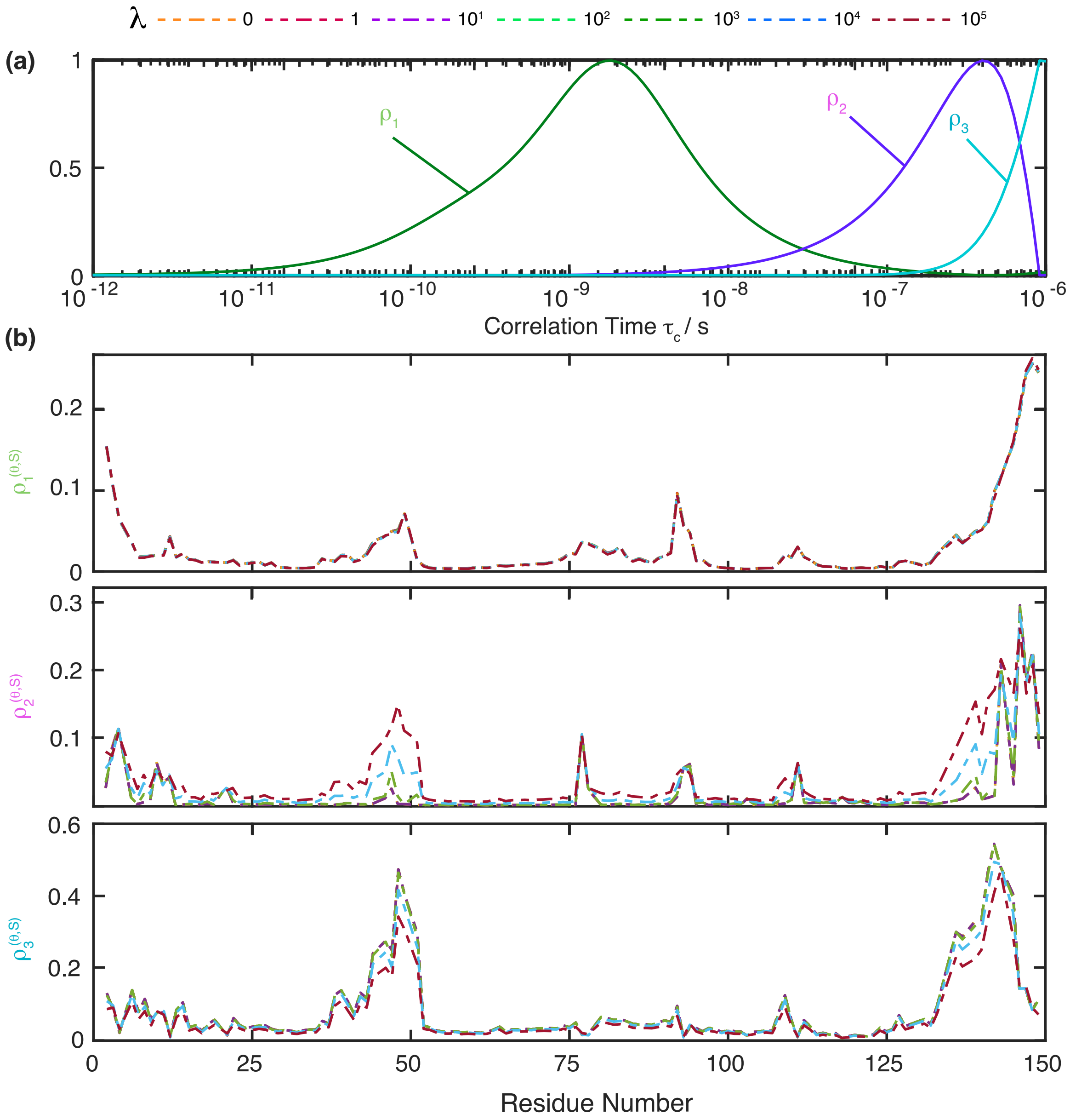

Fig. S18: *Influence of the regularization parameter on detector responses for the detector sensitivities obtained from the experimental conditions*. **(a)** Detector sensitivities ρ_1, ρ_2, ρ_3 constructed from the experimental conditions as used in the main text. **(b)** Detector responses for the experimental detector sensitivities, using the inverse Laplace transforms obtained with regularization parameters from $\lambda = 0$ to $10^5$and computed over the correlation time interval $\tau_c = [10^{-14}, 10^{-6}\,]s$.

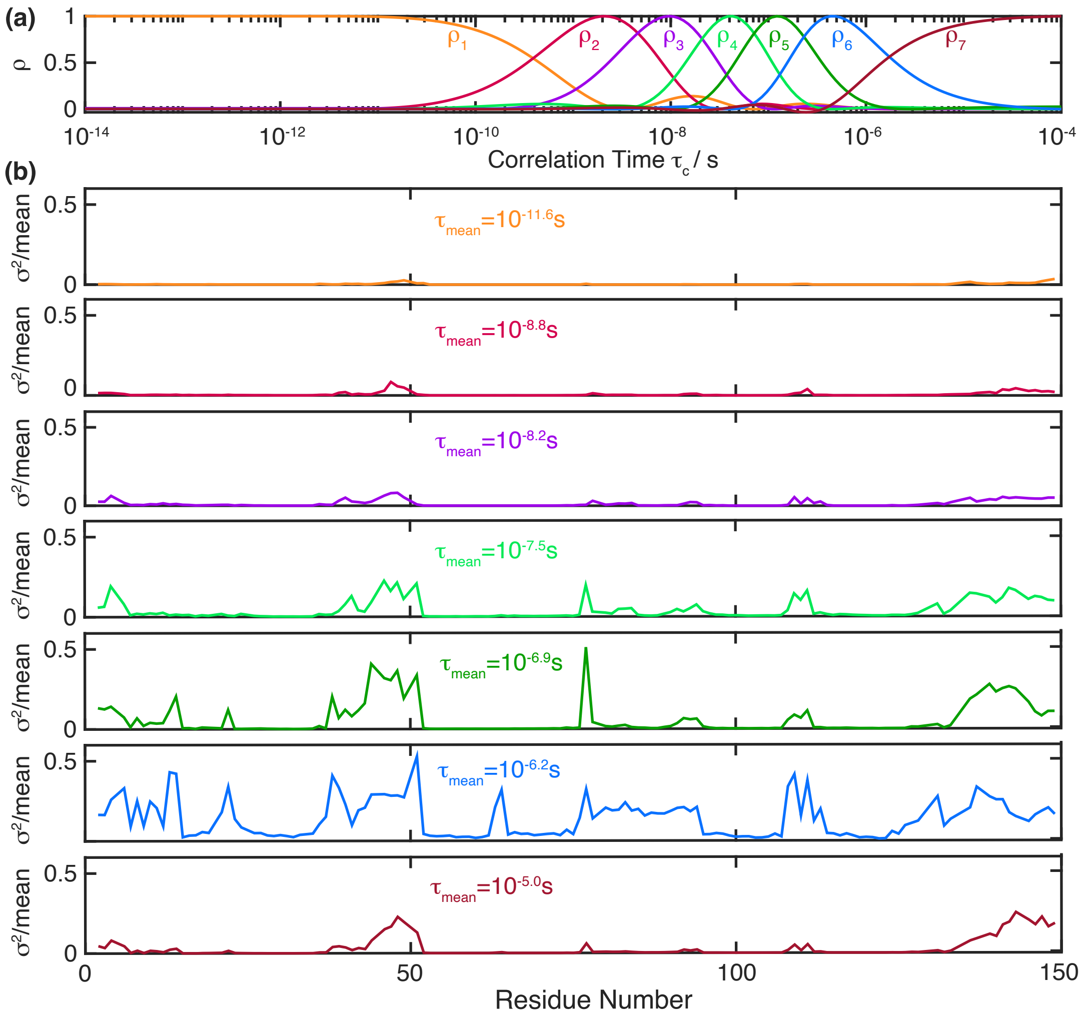

Fig. S19: *Error statistics over 60 copies of Cp149*. Site-specific squared standard deviation over the detector responses from the 60 copies of Cp149 divided by the respective mean for the detectors spaced at each order of magnitude from Fig.S16(a). Inverse Laplace transforms and detector profile optimization have been performed with $\lambda = 0$ over the correlation time interval $\tau_c = [10^{-14}, 10^{-4}]s$.

S1.3 Statistical confidence in detector responses at longer correlation times can be improved by averaging over 60 copies of the molecule.

Using a 1 μs trajectory implies that the statistical significance of nanosecond predictions should be better than the one of microsecond predictions, since the nanosecond motions are much better monitored during the length of the 1 μs trajectory. This improves the extraction of these motions from the correlation function. A possibility to estimate and improve the statistical significance of predictions at all timescales, would be to run a large number of molecular dynamic trajectories with different starting conditions and compare the obtained results. Due to the heavy computational demands for a large viral assembly, this cannot be applied on Cp149. However, since the published MD trajectory contains information on the trajectory of 60 copies of the Cp149 molecule, it is alternatively possible to increase the statistical significance of the predictions for slower motions by averaging over the distribution

of detector responses generated by all 60 copies, which we eventually also used to define the estimated error on the MD prediction (grey shaded area in Fig.3).
Prior to use such an approach, it is important to find out if the motion of the different copies is correlated, as strong correlations between the copies could bias the result. In the following we will discuss how to investigate the presence of such correlations and that they don't play a significant role for Cp149 in particular for slower motions, supporting the validity of an averaging approach over the 60 molecular copies.

We identify correlations based on calculations of the 60 detector responses for the correlation functions calculated from the *first* and the *second* half of the trajectory (both extending over a length of $500\ \mathrm{ns}$). Two methods of error statistics are then performed on these 2×60 detector responses on different timescales. The first method will lead to the correct variance of the statistics, while the second will lead to a different value only if the copies can be considered to be correlated.

We generalize the problem and consider 2 x N samplings of a distribution (mean: μ , standard deviation: σ , in our case N=60). There, the two copies of the sample are independent of each other (1st and 2nd half of the trajectory being independent), but each set of N samplings may not be (there might be a correlation between copies which we want to find out).
Method 1, consists in calculating the mean over the copies and variance over $1_{st}/2_{nd}$ half. We consider that $E[x_{i,1}] = E[x_{i,2}] = E[x_{j,1}] = E[x_{j,2}]$ (all the variables are distributed the same way). Further note that $E[x_{i,1}x_{i,2}] = E[x_{i,1}]E[x_{i,2}] = E[x_{i,1}]^2$ as we assume that the two copies are independent. We thus obtain from this approach the correct variance σ^2 of the distribution (result of equation [1]).

$$
\begin{aligned}
&E\left[\frac{1}{N}\sum_i^N \left(x_{i,1} - \frac{1}{2}(x_{i,1} + x_{i,2}\,)\right)^2 + \left(x_{i,2} - \frac{1}{2}(x_{i,1} + x_{i,2})\,\right)^2\right] \\
&= E\left[\frac{1}{N}\sum_i^N \left(x_{i,1}^2 - x_{i,1}^2 - x_{i,1}x_{i,2} + \frac{1}{4}x_{i,1}^2 + \frac{1}{4}x_{i,2}^2 + \frac{1}{2}x_{i,1}x_{i,2}\right)\right. \\
&\qquad \left. + \left(x_{i,2}^2 - x_{i,2}^2 - x_{i,1}x_{i,2} + \frac{1}{4}x_{i,1}^2 + \frac{1}{4}x_{i,2}^2 + \frac{1}{2}x_{i,1}x_{i,2}\right)\right] \\
&= E\left[\frac{1}{N}\sum_i^N \left(\frac{1}{2}x_{i,1}^2 + \frac{1}{2}x_{i,2}^2 - x_{i,1}x_{i,2}\right)\right] = \frac{1}{N}\sum_i^N E[x_{i,1}^2] - E[x_{i,1}]E[x_{i,2}] \\
&= E[x_{i,1}^2] - \left(E[x_{i,1}\,]\right)^2 = \sigma^2
\end{aligned}
\qquad [1]
$$

In method 2, the variance is taken over the 60 potentially correlated copies, and then the average over the uncorrelated pair of data (1_{st} and 2_{nd} half). The variance over the 60 copies is yield by $E[x_{i,1}^2] - E[x_{i,1}]^2$ as following (the expectation for the average over both halves

should be the same as the expectation for just one half). We can assume that the sum over all covariances relative to some index i is the same regardless of the starting index.

$$E\left[\frac{1}{N-1}\sum_{i}^{N}\left(x_{i,1}-\frac{1}{N}\sum_{i}^{N}x_{j,1}\right)^{2}\right]=\frac{1}{N-1}E\left[\sum_{i}^{N}\left(x_{i,1}^{2}-\frac{2}{N}\sum_{i}^{N}x_{i,1}x_{j,1}+\frac{1}{N^{2}}\sum_{i}^{N}x_{j,1}x_{k,1}\right)\right]$$

$$=\frac{1}{N-1}\sum_{i}\left(E\left[x_{i,1}^{2}\right]-\frac{2}{N}E\left[x_{i,1}^{2}\right]-\frac{2}{N}\sum_{j\neq i}E\left[x_{i,1}x_{j,i}\right]+\frac{1}{N^{2}}\sum_{j}E\left[x_{j,1}^{2}\right]+\frac{1}{N^{2}}\sum_{\substack{j,k\\k\neq j}}E\left[x_{j,1}x_{k,1}\right]\right)$$

$$=\frac{1}{N-1}(N-2+1)E\left[x_{i,1}^{2}\right]+$$

$$+\frac{1}{N-1}(-2)\sum_{j\neq i}\left(E\left[x_{i,1}\right]^{2}+\operatorname{cov}\left(x_{i,1},x_{j,1}\right)\right)+\frac{1}{N}\sum_{\substack{j,k\\k\neq j}}\left(E\left[x_{j,1}\right]^{2}+\operatorname{cov}\left(x_{j,1},x_{k,1}\right)\right)$$

$$=E\left[x_{i,1}^{2}\right]+\frac{1}{N-1}\left(-2(N-1)+(N-1)\right)E\left[x_{i,1}\right]^{2}$$

$$+\frac{1}{N-1}\left(-2\sum_{j\neq i}\operatorname{cov}\left(x_{i,1},x_{j,1}\right)+\frac{1}{N}\sum_{\substack{j,k\\k\neq j}}\left(\operatorname{cov}\left(x_{j,1},x_{k,1}\right)\right)\right)$$

$$=E\left[x_{i,1}^{2}\right]-E\left[x_{i,1}\right]^{2}+\frac{1}{N-1}\left(-2\sum_{j\neq i}\operatorname{cov}\left(x_{i,1},x_{j,1}\right)+\frac{1}{N}\sum_{\substack{j,k\\j\neq k}}\operatorname{cov}\left(x_{j,1},x_{k,1}\right)\right)$$

$$=\sigma\left(x_{i,1}^{2}\right)+\frac{1}{N-1}\left(-2\sum_{j\neq i}\operatorname{cov}\left(x_{i,1},x_{j,1}\right)+\sum_{j\neq k}\operatorname{cov}\left(x_{j,1},x_{k,1}\right)\right)$$

$$=\sigma\left(x_{i,1}^{2}\right)-\frac{1}{N-1}\sum_{j\neq i}\operatorname{cov}\left(x_{i,1},x_{j,1}\right).\qquad[2]$$

According to equation [2] if the covariance (correlation) is zero between copies, the two methods should yield the same result. However, if there is a positive/negative covariance between copies then the variance calculated by method 2 is smaller/higher than the one calculated with method 1.

Fig.S20 displays the results of the error statistics applying method 1 (blue, correlation unbiased) and method 2 (green dashed, correlation biased) to the detector responses of the 60 copies at the seven detector sensitivities shown in Fig.S17(a). The results for both methods

agree to a very good degree over most portions of the molecule. The only regions where the two methods show larger deviations are the loop from 44-50 and the last part of the molecule approaching the C-ter (on the other side, the N-ter does not show any large correlations). We observe slight tendency to an increased difference between the two methods for the slowest detector, hinting to an increased correlation between the motions. Overall these results indicate that the MD error analysis can be improved, by averaging over the copies without having to account for covariance between copies, avoiding the computationally demanding step of performing a multitude of different MD 1 μs trajectories using different starting conditions.

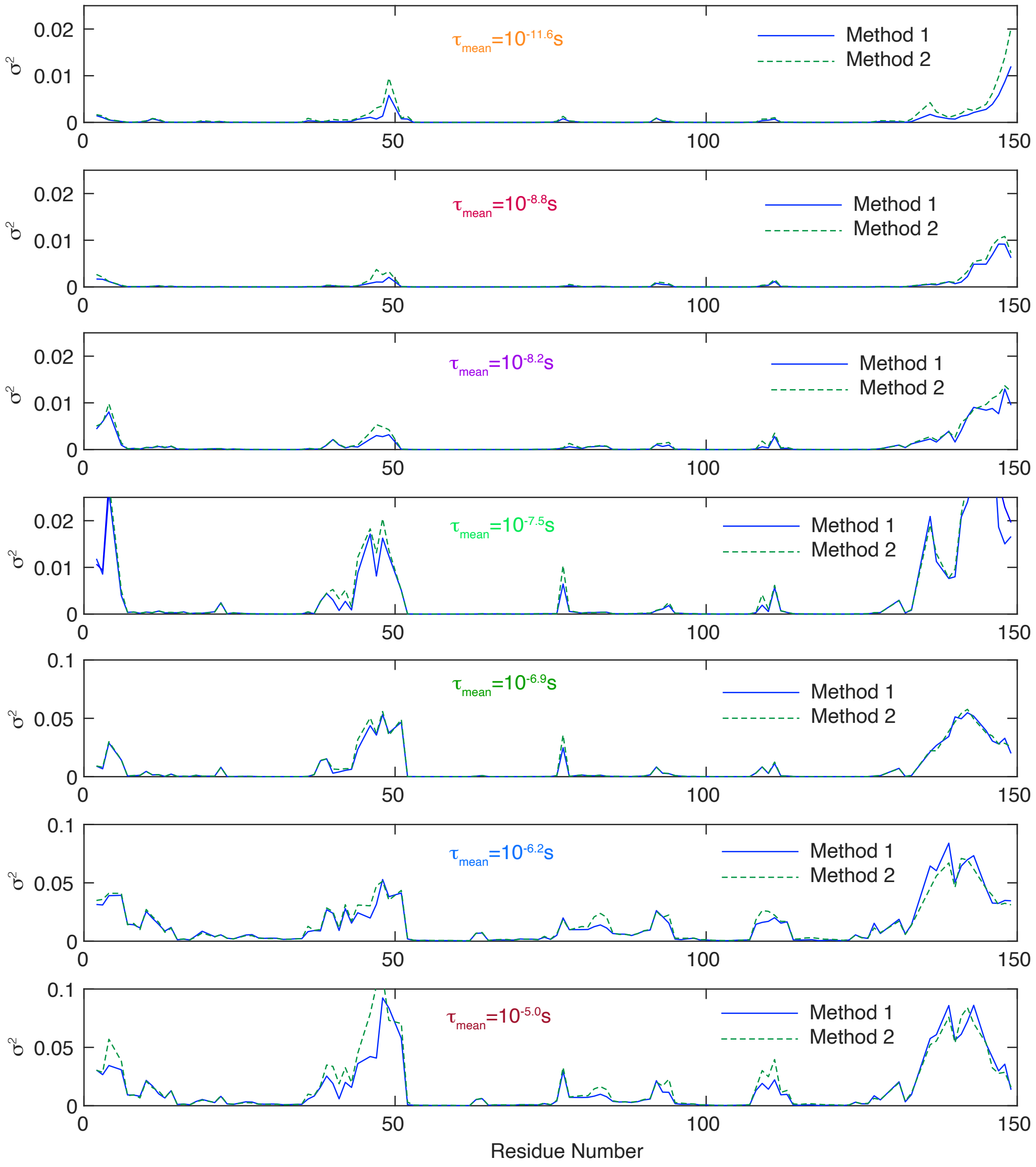

Fig. S20: Cp149 MD error statistics using method 1 (correlation unbiased) and method 2 (correlation biased) discussed in section S1.3 for the detector sensitivities in Fig.S17(a). Inverse Laplace transforms and detector profile optimization have been performed over the correlation time interval $\tau_c = [10^{-14}, 10^{-4}\,]s$.

S1.4: Estimation of the total MD error at different timescales

The total estimated error on the quantitative MD prediction of amplitudes of motions at different timescales (grey shaded area in Fig.3) could be constructed combining the two effects we described above (S1.1 and S1.2).

The general procedure for the error estimation is sketched in Fig.S21(a). We start by extracting for each of the 60 copies a correlation function for the first and second half of the trajectory as well as for the full trajectory. Each correlation function is inverse Laplace transformed with 5 logarithmically-spaced regularization parameters λ ranging from $[0,10^5]$. Note that this range of values corresponds to regularization parameters which lead to a good fit of the input correlation function and therefore representing a valid solution of the inverse-Laplace transform problem (as it has been shown in Fig.S16(a)).
The result comprises 3x($1^{st}/2^{nd}$,full)x60(copies)x5(λ)= 900 distributions of motion. We now extract detector responses for all distributions of motion and perform an average over all 60 copies for each regularization parameter and for the $1^{st}/2^{nd}$ half and the full trajectory. This leaves us with 3x5 averaged responses. We have shown in section S1.3 that it is possible to perform such an averaging over copies, without having to account for strong correlation between copies. For each regularization parameter λ we define a grey shaded area, which ranges from the minimum to the maximum of all detector responses for the $1^{st}/2^{nd}$ half and the full trajectory. This represents the amount of the MD error that can account for the non-equilibration of slower motions within the length of the trajectory (see section S.1.2). The grey shaded area in Fig.S21 shows this portion of the MD error for $\lambda = 0$. As it can be seen the non-equilibration error grows systematically for slower motion compared to the one for faster motions. The blue shaded area in Fig.S21 is created by taking the minimum and maximum value of the averaged detector responses over the grey shaded areas calculated for all regularization parameters λ. For this reason, the grey shaded area for $\lambda = 0$ shown in Fig.S21 is always contained in the blue shaded area and the difference between blue and grey shaded area accounts for the portion of the MD error that can be attributed to the instability of the detector responses with respect to the choice of the regularization parameter. As illustrated in section S.1.1 this second error source remains however much smaller. Fig. S22 estimates the MD error according to the same procedure for the detectors $\rho_{1.3\mathrm{ns}}, \rho_{0.2\mu\mathrm{s}}, \rho_{0.6\mu\mathrm{s}}$ constructed from the experimental conditions and used for the analysis in the main text. As already seen in Fig. S18, due to slower motion being ill-defined, based on the time points available in the trajectory and the narrowness of the $\rho_{0.6\mu\mathrm{s}}$ detector due to the ending of the trajectory, the regularization error (blue-shaded) is substantially larger for the slower motions approaching $1\ \mu s$, thereby becoming an important contribution for the total error.

In summary, the combination of both sources of MD error in a total MD error estimate for detector responses obtained from independent detectors spaced at each order of magnitude (blue shaded area in Fig.S21) shows that around timescales in the order of 10^{-7}s the MD uncertainty starts growing significantly and becomes particularly large when using the detectors approaching the end of the trajectory obtained from the experimental conditions used in the main text. For these reasons, we propose to set the boundary of the

green and grey areas for quantitative MD predictions for Cp149, using a detectors approach to 10^{-7}s, which is around one order of magnitude smaller than the length of the trajectory.

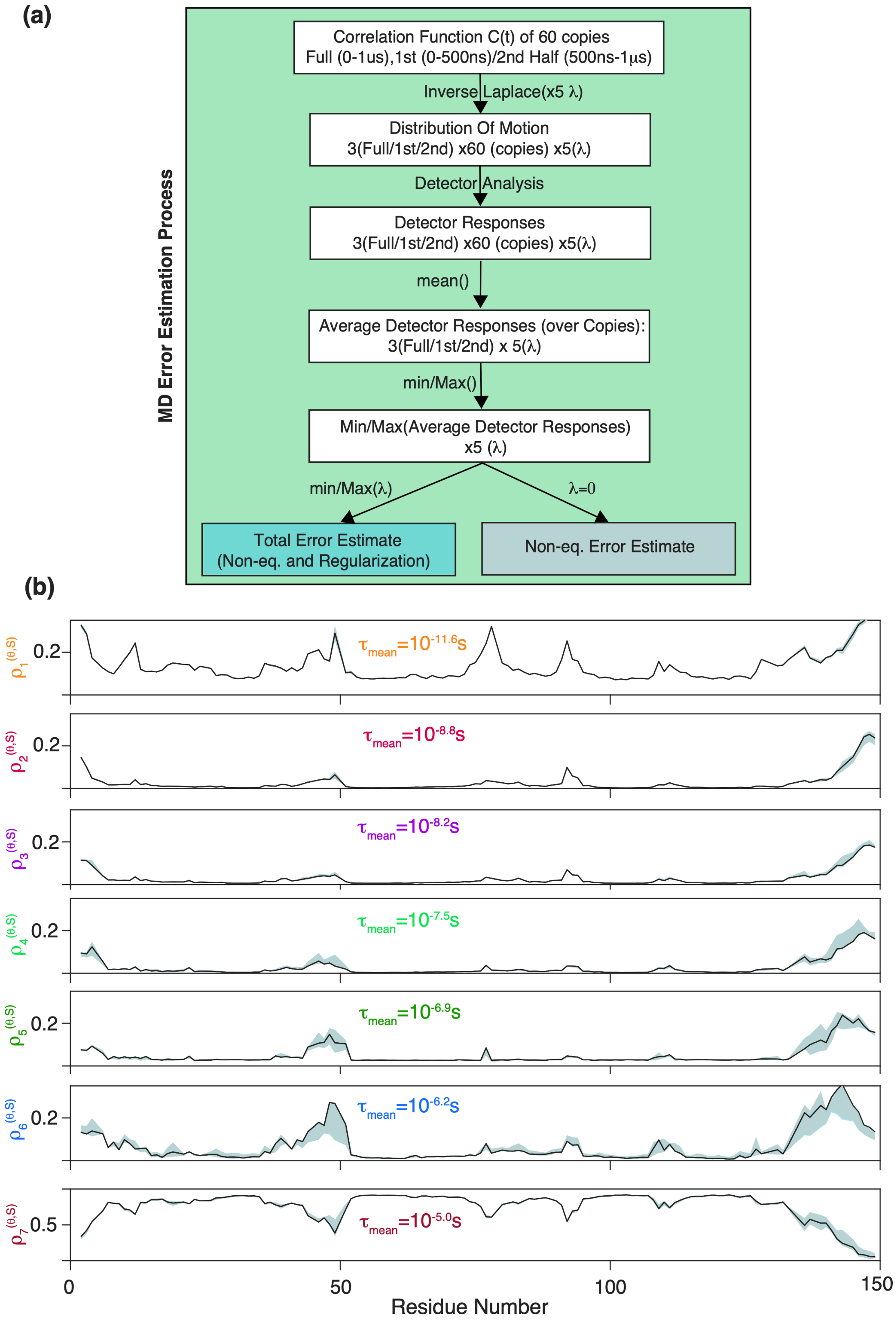

Fig. S21: Schematic summary of the procedure used for the MD error estimation **(a)**. Detector responses and corresponding MD error estimate for the seven detectors spaced at each order of magnitude from Fig.S17(a). The black line corresponds to the average detector response over all 60 copies for the full trajectory for $\lambda = 0$. The grey shaded area is comprised in between the maximum and minimum values of the average detector

response for the 1st/2nd half and the full trajectory for $\lambda = 0$. The blue shaded are corresponds to the maximum and minimum values of all grey shaded areas that have been calculated over the 5 λ values.

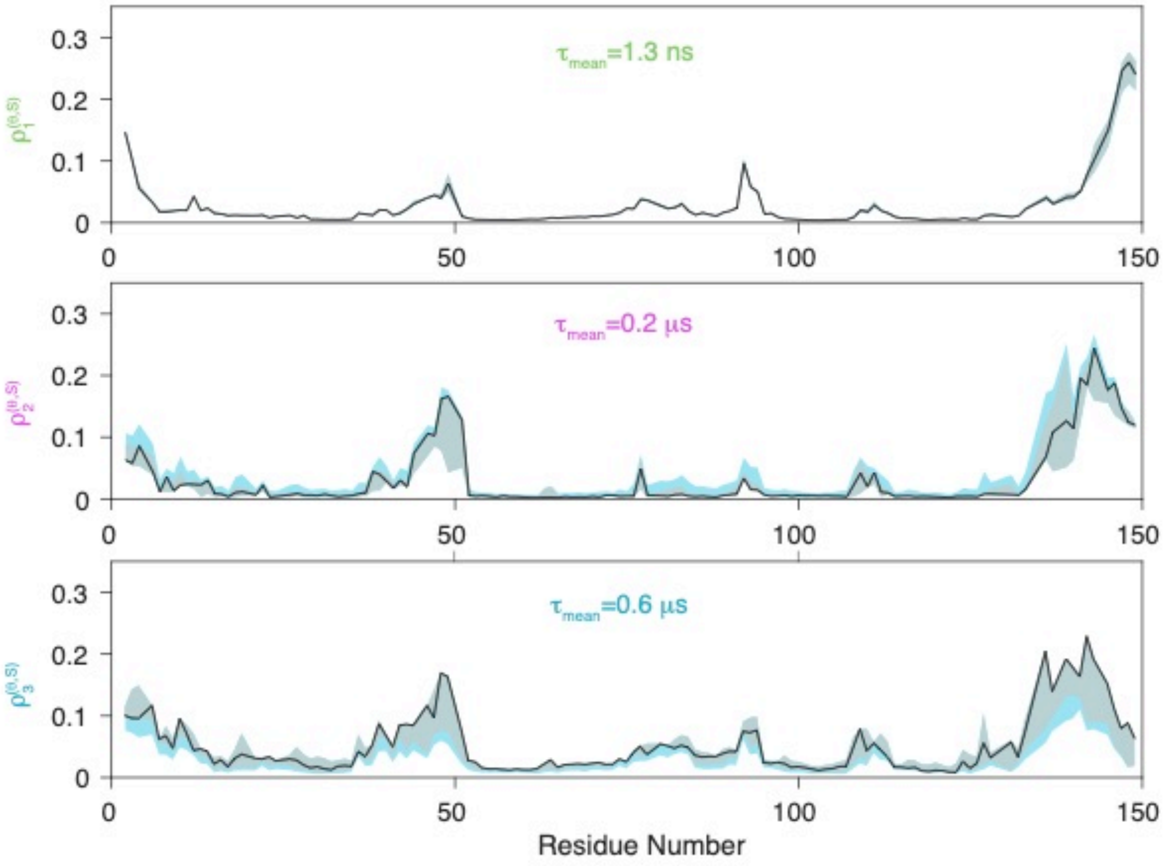

Fig. S22: Detector responses and corresponding MD error estimate for the detectors ρ_1, ρ_2, ρ_3 constructed from the experimental conditions and used in the main text. The black line corresponds to the average detector response over all 60 copies for the full trajectory for $\lambda = 0$. The grey shaded area is comprised in between the maximum and minimum values of the average detector response for the 1st/2nd half and the full trajectory for $\lambda = 0$. The blue shaded are corresponds to the Maximum and Minimum values of all grey shaded areas that have been calculated over the 5 λ values.

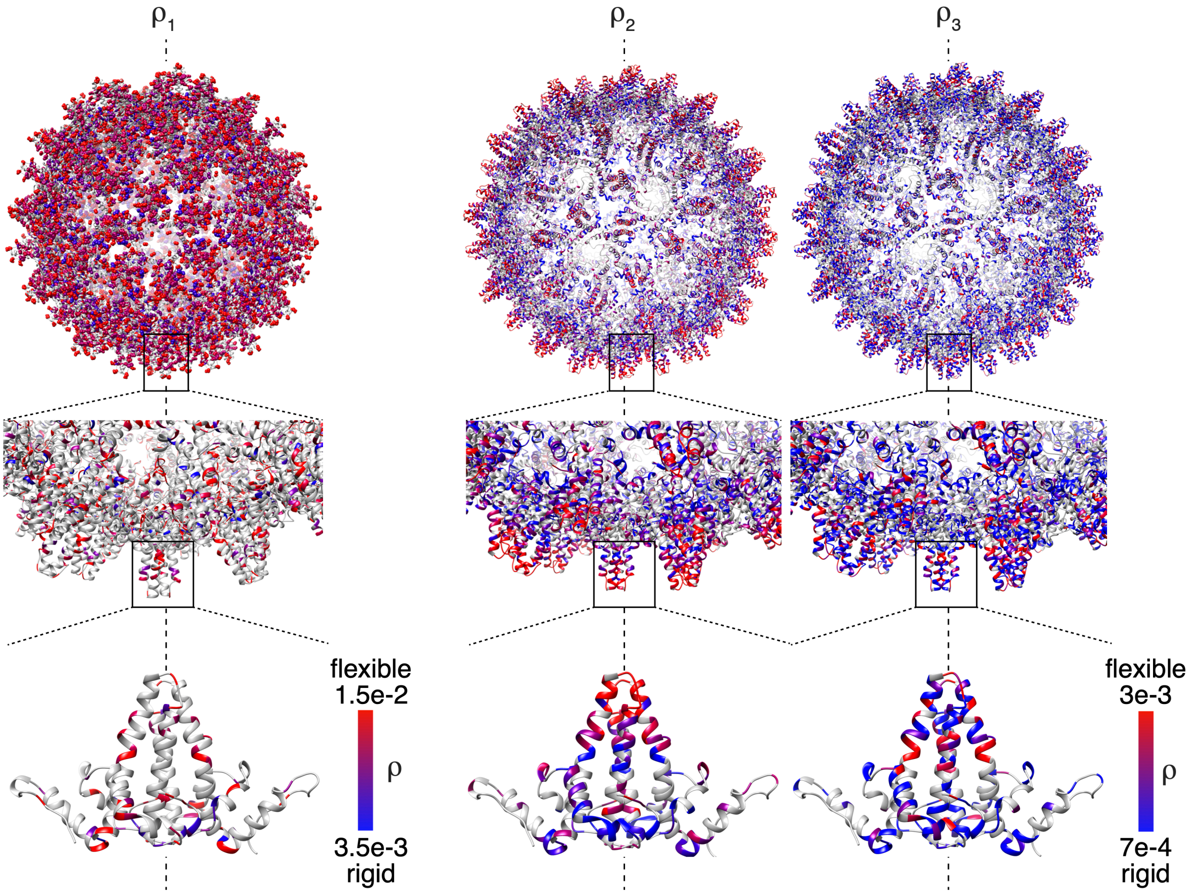

Fig. S23: NMR amplitude of motions $\rho_1^{(\theta,S)}$, $\rho_2^{(\theta,S)}$ and $\rho_3^{(\theta,S)}$ shown in a red to blue gradient on the Cp149 dimer structure (PDB 1QGT) at different scales from the full capsid (top) to the dimer (bottom). Residues not characterized by NMR are shown in grey.

Section S2: Raw Data Collection

Fig. 2c),e)

RESIDUE	R_1 (^{15}N)·	$R_{1\rho}$(160 kHz)	$R_{1\rho}$(80 kHz)	$\sigma(R_1$ (^{15}N))·	$\sigma(R_{1\rho}$(160 kHz))	$\sigma(R_{1\rho}$(80 kHz))
2.0000000e+00	1.0540655e-01	4.8224527e+00	8.6317903e+00	1.1389296e-02	6.5085916e-01	6.5085916e-01
4.0000000e+00	3.4220751e-02	1.3964034e+00	1.7859072e+00	5.9009532e-03	1.7640116e-01	1.7640116e-01
7.0000000e+00	1.4450570e-02	1.7580637e+00	2.2928553e+00	9.9471477e-03	5.9194235e-01	5.9194235e-01
8.0000000e+00	2.3036486e-02	1.2658516e+00	1.9742078e+00	9.1094204e-03	3.0263326e-01	3.0263326e-01
1.0000000e+01	2.3426822e-02	1.3688783e+00	1.4054437e+00	5.4656139e-03	3.3219769e-01	3.3219769e-01
1.2000000e+01	1.7511801e-02	8.4987106e-01	1.1713085e+00	3.7736286e-03	4.0261549e-01	4.0261549e-01
1.3000000e+01	1.4409699e-02	1.4923500e+00	1.6434742e+00	3.3025709e-02	2.8324652e+00	2.8324652e+00
1.7000000e+01	6.3080626e-02	5.3603673e+00	5.4429928e+00	2.4427250e-02	2.1412116e+00	2.1412116e+00
2.4000000e+01	2.3554213e-02	1.1447390e+00	1.4963143e+00	1.3845149e-02	6.0265997e-01	6.0265997e-01
3.4000000e+01	4.8803267e-02	3.3491042e+00	4.1687628e+00	2.0866780e-02	7.8138503e-01	7.8138503e-01
3.5000000e+01	2.4610352e-02	8.1390604e-01	2.1939225e+00	2.6264488e-02	9.0558061e-01	9.0558061e-01
3.7000000e+01	5.7211796e-02	8.9689757e-01	1.3398380e+00	1.8297982e-02	1.1727341e+00	1.1727341e+00
3.8000000e+01	2.8563935e-02	3.4615949e+00	4.5700503e+00	9.7013693e-03	8.4747226e-01	8.4747226e-01
4.4000000e+01	2.9928295e-02	1.0504366e+00	1.6863328e+00	1.9817529e-03	2.2964037e-01	2.2964037e-01
4.6000000e+01	1.2508586e-01	2.2578535e+00	3.8812794e+00	6.6469774e-03	2.4342010e-01	2.4342010e-01
4.8000000e+01	1.8360397e-02	2.8154332e+00	5.0181936e+00	5.4691018e-03	2.9476729e-01	2.9476729e-01
5.1000000e+01	1.5500842e-02	1.5879731e+00	1.6877173e+00	4.0046187e-03	4.2017585e-01	4.2017585e-01
5.7000000e+01	3.2722845e-02	1.6193684e+00	1.1480649e+00	1.3576039e-02	8.6728077e-01	8.6728077e-01
6.9000000e+01	2.8848428e-02	1.8296206e+00	2.5511890e+00	4.0648253e-03	5.0959351e-01	5.0959351e-01
7.1000000e+01	2.7199109e-02	4.2679455e+00	5.4364818e+00	9.3526611e-03	1.6488410e+00	1.6488410e+00
7.3000000e+01	1.8573447e-02	3.3947580e+00	5.5149586e+00	3.9494401e-03	5.8533211e-01	5.8533211e-01
7.4000000e+01	8.4141452e-01	1.0626454e+01	1.1171273e+01	2.8439087e-01	4.7916272e+00	4.7916272e+00
7.7000000e+01	9.3001360e-02	1.2652540e+01	1.1662786e+01	5.8045722e-03	5.0971149e+00	5.0971149e+00
8.7000000e+01	2.9750799e-02	6.2884615e+00	1.3261963e+01	8.8917966e-03	1.3640309e+00	1.3640309e+00
9.1000000e+01	3.2853861e-02	6.4930764e+00	8.9392340e+00	1.8443537e-02	1.5740904e+00	1.5740904e+00
9.3000000e+01	2.9631415e-02	1.4274583e+00	2.3016065e+00	1.0052168e-02	1.0107345e+00	1.0107345e+00
9.4000000e+01	4.6186182e-02	4.6258801e+00	1.0447628e+01	1.9850706e-02	2.7423054e+00	2.7423054e+00
9.5000000e+01	2.8954767e-02	2.1700969e+00	3.2294028e+00	1.3931750e-02	1.3138835e+00	1.3138835e+00
1.0900000e+02	4.9128142e-02	1.5309094e+00	1.7596221e+00	1.2877746e-02	6.5887356e-01	6.5887356e-01
1.1100000e+02	1.1673068e-02	8.7878568e-01	2.2029550e+00	7.5669908e-03	3.6286356e-01	3.6286356e-01
1.1200000e+02	4.4352888e-02	1.2326237e+00	1.6546068e+00	5.3522980e-03	1.4327052e-01	1.4327052e-01
1.1300000e+02	4.4900480e-02	1.1469227e+00	1.4733509e+00	3.4029154e-03	1.6003663e-01	1.6003663e-01
1.1500000e+02	2.0043538e-02	1.4748564e+00	2.1189720e+00	4.9917856e-03	1.1547373e+00	1.1547373e+00
1.2800000e+02	2.3816620e-02	1.5762640e+00	2.1022682e+00	5.8228828e-03	3.3441456e-01	3.3441456e-01
1.3700000e+02	4.7976846e-02	4.4121479e+00	4.6997878e+00	1.1433341e-02	1.1537778e+00	1.1537778e+00

Fig. 2d)

RESIDUE	$R_{1\rho}$(110 kHz)	$R_{1\rho}$(80 kHz)	$\sigma(R_{1\rho}$(110 kHz))	$\sigma(R_{1\rho}$(80 kHz))
2.0000000e+00	7.3542364e+00	1.2783631e+01	2.5116495e+00	2.6359786e+00
3.0000000e+00	2.0209421e+00	2.1478952e+00	7.4700909e-01	5.6727848e-01
4.0000000e+00	2.1186005e+00	2.0825228e+00	9.8853924e-01	8.2820079e-01
7.0000000e+00	8.2253787e-01	1.0392561e+00	1.9253819e+00	7.3841464e-01
8.0000000e+00	-2.9460289e-01	7.3262959e-01	1.4891543e+00	5.7218227e-01
1.0000000e+01	1.0248454e+00	1.1331154e+00	4.2920288e-01	4.0744134e-01
1.1000000e+01	1.5396067e+00	1.4871375e+00	5.3440028e-01	4.9955551e-01
1.2000000e+01	1.1585717e+00	1.1401121e+00	2.9192779e-01	5.3091528e-01
1.3000000e+01	-3.2443472e-01	2.4165060e-01	1.9394218e+00	7.9603325e-01
1.5000000e+01	1.0618972e+00	2.0853491e+00	1.0134327e+00	1.8123533e+00
2.1000000e+01	2.2653047e+00	2.4294394e+00	1.0040950e+00	8.1556606e-01
2.2000000e+01	2.3811287e+00	2.3326494e+00	6.4739638e-01	1.0510630e+00
2.3000000e+01	1.6803123e+00	1.7921486e+00	1.3769892e+00	1.5360040e+00
2.6000000e+01	9.7125532e-01	2.1462451e+00	1.1037405e+00	9.6905922e-01
2.7000000e+01	1.3374485e+00	1.7329416e+00	7.8975740e-01	6.3054445e-01
2.8000000e+01	2.7599049e+00	4.6721100e+00	5.3334507e-01	7.2737699e-01
2.9000000e+01	1.6224118e+00	1.4377054e+00	1.2950821e+00	6.6079393e-01
3.2000000e+01	2.9742039e+00	1.2576677e+00	3.0541608e+00	1.0701465e+00

3.3000000e+01	1.9784267e+00	1.5802738e+00	7.1198047e-01	7.7331867e-01
3.5000000e+01	9.7621848e+00	1.2521482e+00	1.4309972e+01	1.0561276e+00
3.6000000e+01	1.0342273e+00	1.9862102e+00	9.4521887e-01	2.0392113e+00
3.8000000e+01	1.0797620e+00	2.4247674e+00	7.5789293e-01	1.6448726e+00
3.9000000e+01	1.8941610e+00	1.2050021e-01	1.3476161e+00	4.6622032e-01
4.0000000e+01	6.4489825e-01	1.6189542e+00	7.9261606e-01	1.0633685e+00
4.1000000e+01	4.8100866e-01	1.1072423e+00	6.6546556e-01	1.2483646e-01
4.2000000e+01	9.6931300e-01	1.0626449e+00	9.5692598e-01	6.4596039e-01
4.3000000e+01	3.2442819e-01	2.4642739e+00	1.1967527e+00	1.6441642e+00
4.4000000e+01	1.0310796e+00	1.0925526e+00	4.5925129e-01	3.5817180e-01
4.6000000e+01	2.1671245e+00	2.0870020e+00	4.8883506e-01	4.2866121e-01
4.8000000e+01	2.3548545e+00	2.9064553e+00	2.5557409e-01	3.6859101e-01
4.9000000e+01	2.2374354e+00	2.8791333e+00	4.3180815e-01	8.7829650e-01
5.1000000e+01	1.5068885e+00	1.0021604e+00	7.2579141e-01	4.5534451e-01
5.2000000e+01	1.2261741e+00	4.7302439e-01	3.4510598e-01	7.0393013e-01
5.3000000e+01	1.5696453e+00	1.4517121e+00	6.0395829e-01	6.5399395e-01
5.5000000e+01	4.4336113e+00	-8.1184358e-02	2.6438045e+00	4.0654699e+00
5.6000000e+01	2.8368454e+00	1.7274009e+00	1.0216368e+00	7.8529504e-01
6.0000000e+01	2.0540228e+00	2.0413544e+00	1.1147465e+00	1.4854137e+00
6.3000000e+01	2.8487829e-01	1.8832853e+00	1.3526173e+00	4.7871320e-01
6.4000000e+01	1.5950157e+00	1.4169949e+00	7.8799454e-01	1.3131315e+00
6.6000000e+01	-6.1976169e-01	1.9197152e+00	1.6183664e+00	1.5210387e+00
6.7000000e+01	1.8566393e+00	2.6177777e+00	5.9614283e-01	7.3217592e-01
6.8000000e+01	2.7554194e+00	4.2851427e+00	1.4520906e+00	1.4533425e+00
7.0000000e+01	1.7239270e+00	1.9379055e+00	1.3480287e+00	8.3516903e-01
7.1000000e+01	1.8401002e+00	3.0337195e+00	9.5940022e-01	9.0766304e-01
7.2000000e+01	8.5000000e+00	8.4345411e+00	4.0000000e+00	1.9172276e+00
7.3000000e+01	2.8545278e+00	3.7007446e+00	4.2831545e-01	5.0291563e-01
7.4000000e+01	4.7796380e+00	4.3302085e+00	8.7021302e-01	1.1412258e+00
7.5000000e+01	4.7825768e+00	2.8314201e+00	2.1301217e+00	9.2712461e-01
7.6000000e+01	4.3118731e+00	6.5696876e+00	1.8319763e+00	2.3008043e+00
7.7000000e+01	1.0660000e+01	1.1535847e+01	3.5800000e+00	4.3936374e+00
7.8000000e+01	5.4923355e+00	9.6005719e+00	2.7450756e+00	2.1135918e+00
8.0000000e+01	3.6754980e+00	1.9578215e+00	6.7373190e-01	5.6074766e-01
8.1000000e+01	3.4475684e+00	4.2879293e+00	8.4806765e-01	5.9514860e-01
8.2000000e+01	3.2410237e+00	2.5524502e+00	1.0932626e+00	1.3908241e+00
8.3000000e+01	4.9602847e+00	2.6773612e+00	1.9784637e+00	1.9927474e+00
8.4000000e+01	1.7396366e+00	3.4908775e+00	1.4301501e+00	1.3477445e+00
8.5000000e+01	3.3587368e+00	3.1433146e+00	1.1811338e+00	8.1296838e-01
8.7000000e+01	2.7013260e+00	4.1058983e+00	1.2896709e+00	1.2166328e+00
8.8000000e+01	2.5318191e+00	2.4508599e+00	5.2178977e-01	7.3180012e-01
8.9000000e+01	3.4550018e+00	3.3436057e+00	4.6251617e-01	9.4831056e-01
9.0000000e+01	2.2592086e+00	3.0536846e+00	2.5252530e+00	1.2055103e+00
9.1000000e+01	2.0957338e+00	2.8691281e+00	1.2730378e+00	8.1913127e-01
9.2000000e+01	2.9011023e+00	4.4406091e+00	9.8713958e-01	1.7755952e+00
9.3000000e+01	5.4900944e-01	2.5130856e+00	2.6132699e+00	2.7627795e+00
9.4000000e+01	1.4007486e+00	3.2416079e+00	5.1063784e-01	4.9965703e-01
1.0900000e+02	2.3287129e+00	1.4757627e+00	1.0694286e+00	1.7357291e+00
1.1100000e+02	1.4875158e+00	2.0234279e+00	2.2240882e-01	4.5040058e-01
1.1200000e+02	1.6667154e+00	2.0963110e+00	4.3885126e-01	1.8891431e-01
1.1300000e+02	1.2409655e+00	1.0442385e+00	6.3824675e-01	7.0761928e-01
1.1400000e+02	1.4546196e+00	2.1551624e+00	8.3562917e-01	6.9251253e-01
1.1500000e+02	9.7464033e-01	2.0299835e+00	1.6743197e+00	1.2416096e+00
1.1600000e+02	2.5066103e+00	2.1406910e+00	9.6663555e-01	7.5490685e-01
1.1700000e+02	4.2529614e+00	1.6008887e+00	2.7734441e+00	5.4128658e-01
1.2300000e+02	2.1102708e+00	2.7286377e+00	1.4502383e+00	1.2245458e+00
1.2800000e+02	2.1659930e+00	1.7582609e+00	1.6334288e+00	2.1087784e+00
1.3100000e+02	2.7213581e+00	2.0630858e+00	2.7686085e+00	1.2110821e+00

Fig. 3c)

RESIDUE	$R_{1\rho}$(11°C)	$R_{1\rho}$(14°C)	$R_{1\rho}$(20°C)	$R_{1\rho}$(27°C)	$R_{1\rho}$(34°C)	$\sigma(R_{1\rho}$(11°C))	$\sigma(R_{1\rho}$(14°C))	$\sigma(R_{1\rho}$(20°C))	$\sigma(R_{1\rho}$(27°C))	$\sigma(R_{1\rho}$(34°C))
2.0000000e+00	1.9173504e+00	1.8457075e+00	2.6221694e+00	3.8811940e+00	7.9424519e+00	2.8679608e-01	1.3466764e-01	3.5295828e-01	4.3795920e-01	7.4776581e-01
4.0000000e+00	1.6393163e+00	1.6531248e+00	1.6665640e+00	1.6943718e+00	1.9396658e+00	8.0576653e-02	1.8851606e-01	1.3544464e-01	1.6112768e-01	3.0076582e-01
7.0000000e+00	1.7104849e+00	1.9043484e+00	1.6246877e+00	1.4549701e+00	1.5666352e+00	3.2192386e-01	4.0948942e-01	1.7320735e-01	3.2567815e-01	4.9572949e-01
8.0000000e+00	2.2016946e+00	1.8112446e+00	1.6943493e+00	1.1199079e+00	1.4679673e+00	7.7261239e-01	6.0125681e-01	4.0456085e-01	1.9269153e-01	2.3501369e-01
1.0000000e+01	1.0390267e+00	1.3040103e+00	1.1638436e+00	1.2268130e+00	1.1883742e+00	1.8249479e-01	1.8852977e-01	1.7660170e-01	2.1612920e-01	2.1171775e-01
1.2000000e+01	1.0019561e+00	9.5654741e-01	8.5242151e-01	7.6137237e-01	9.4306175e-01	1.2198122e-01	7.7895121e-02	9.8815247e-02	1.4381500e-01	1.0070970e-01
1.3000000e+01	1.0147072e+00	1.3083375e+00	1.2742313e+00	8.9500089e-01	1.2608969e+00	1.8479528e-01	2.5564089e-01	3.9441078e-01	2.9707206e-01	4.5361635e-01
2.4000000e+01	2.0584053e+00	1.8435216e+00	1.5808942e+00	1.3301478e+00	1.3456531e+00	4.5114313e-01	3.2146118e-01	3.9819655e-01	2.6902319e-01	3.9759997e-01
3.1000000e+01	1.8571722e+00	2.3947379e+00	1.7783873e+00	2.9011241e+00	2.1131750e+00	7.2047355e-01	9.9447483e-01	4.8044918e-01	1.6543804e+00	5.7127784e-01
3.4000000e+01	4.0890497e+00	4.3311542e+00	3.8492921e+00	3.3193544e+00	2.3711063e+00	9.2194426e-01	8.2543118e-01	8.5652534e-01	5.9014826e-01	3.3934192e-01
3.5000000e+01	1.6855887e+00	1.1054706e+00	9.9827482e-01	9.7996475e-01	1.4422975e+00	5.1610818e-01	6.1430665e-01	3.9217447e-01	6.7785297e-01	5.9375243e-01
3.7000000e+01	1.0099233e+00	1.7294586e+00	1.2559527e+00	5.0755596e-01	1.2311522e+00	3.2235283e-01	9.9968228e-01	4.3951951e-01	8.4391988e-01	4.0257599e-01
3.8000000e+01	5.1851233e+00	4.6292636e+00	4.3087783e+00	4.3512477e+00	4.6036652e+00	1.0682046e+00	8.0411529e-01	8.5715694e-01	9.7320827e-01	7.0255859e-01
4.4000000e+01	1.3259643e+00	1.4874497e+00	1.2412216e+00	1.1511802e+00	1.5114116e+00	1.3973154e-01	2.0455221e-01	1.1456875e-01	1.3014620e-01	2.0695606e-01
4.6000000e+01	2.2003983e+00	2.2035141e+00	2.2262947e+00	2.6757756e+00	3.6716414e+00	8.6057138e-02	1.1278429e-01	9.1483943e-02	2.2323429e-01	2.4684653e-01
4.8000000e+01	1.7658503e+00	1.9772447e+00	2.4878569e+00	4.0337685e+00	6.0730438e+00	1.1228988e-01	8.3246694e-02	1.8085309e-01	3.8677194e-01	4.4570532e-01
5.1000000e+01	1.8254291e+00	1.6400093e+00	1.7113075e+00	1.6926003e+00	2.4735189e+00	1.1360309e-01	2.1186754e-01	1.9328734e-01	3.1957592e-01	1.4190286e-01
6.3000000e+01	2.1764145e+00	1.0270190e+00	1.4095113e+00	1.5179011e+00	1.2550973e+00	3.5200636e-01	4.5145338e-01	4.1423291e-01	6.9343485e-01	7.4215817e-01
6.5000000e+01	2.4197444e+00	1.7452155e+00	1.5965759e+00	2.3792536e+00	1.9198511e+00	3.3056366e-01	6.2085078e-01	4.3780408e-01	5.8622677e-01	1.0140892e+00
6.9000000e+01	1.9791571e+00	2.0740749e+00	1.9467929e+00	1.8530288e+00	2.1297154e+00	2.1058677e-01	1.9443860e-01	2.8697723e-01	2.3425544e-01	2.1234310e-01
7.1000000e+01	3.0006243e+00	2.9752123e+00	2.6061567e+00	2.4243253e+00	2.6940943e+00	2.4190609e-01	2.1409917e-01	2.3405935e-01	3.8080873e-01	3.6788904e-01
7.3000000e+01	3.9104608e+00	3.7620111e+00	3.4918551e+00	2.8994532e+00	2.9066593e+00	1.3909143e-01	3.9701528e-01	1.7720805e-01	2.3381499e-01	2.7238740e-01
7.4000000e+01	5.8942434e+00	5.6214748e+00	3.9859181e+00	3.7842039e+00	3.4134086e+00	7.5699421e-01	8.1583214e-01	4.7134043e-01	5.1234617e-01	2.3935458e-01
7.7000000e+01	8.5030540e+00	8.1386763e+00	8.6669236e+00	9.1800335e+00	8.9436129e+00	1.5565960e+00	1.2256411e+00	1.7413664e+00	1.8377263e+00	2.3303287e+00
8.7000000e+01	3.7725028e+00	3.7278801e+00	3.2634120e+00	3.1811515e+00	3.9638187e+00	2.4082357e-01	2.8056112e-01	3.1179801e-01	4.0630413e-01	4.9797441e-01
9.1000000e+01	3.5314683e+00	3.1949099e+00	2.9962045e+00	2.7576206e+00	3.1909875e+00	3.2901593e-01	5.5600591e-01	4.7605130e-01	3.1347217e-01	3.6827294e-01
9.4000000e+01	2.0210349e+00	1.9944640e+00	2.3177685e+00	3.2170640e+00	2.8226548e+00	2.4668496e-01	4.9244409e-01	2.1867062e-01	2.6028438e-01	3.2999859e-01
9.5000000e+01	2.1719002e+00	1.9522917e+00	1.8369387e+00	1.6976420e+00	2.3571445e+00	2.2254478e-01	2.3724168e-01	1.9176308e-01	4.1633979e-01	8.1973631e-01
1.1100000e+02	1.2383271e+00	1.4732991e+00	1.6818496e+00	1.5368784e+00	1.3457325e+00	2.9785695e-01	1.9323839e-01	3.6643321e-01	2.6972867e-01	2.6096149e-01
1.1200000e+02	2.0303395e+00	1.9663945e+00	1.6917817e+00	1.5147594e+00	1.3288592e+00	1.7818913e-01	1.2178813e-01	1.8910400e-01	2.3061650e-01	1.4500916e-01
1.1300000e+02	1.8636383e+00	1.7661285e+00	1.5778792e+00	1.4169960e+00	1.3846344e+00	9.8695480e-02	7.0845974e-02	1.1697374e-01	9.0730575e-02	2.0409179e-01
1.1500000e+02	2.0364131e+00	1.9759320e+00	2.0119473e+00	1.8916052e+00	1.6966305e+00	3.8023759e-01	2.3742405e-01	3.1362023e-01	3.1314024e-01	2.5095019e-01
1.2200000e+02	8.1105398e+00	8.4034389e+00	7.5669427e+00	6.0982644e+00	8.9224319e+00	1.0655961e+00	1.2478253e+00	9.1750277e-01	4.8468079e-01	5.5350379e-01
1.2300000e+02	1.4466548e+00	1.4050232e+00	1.1078055e+00	9.9430869e-01	1.3660049e+00	2.7089614e-01	4.5460977e-01	4.9032151e-01	5.6572883e-01	4.4109366e-01
1.2800000e+02	1.7774234e+00	1.9717008e+00	1.7413889e+00	1.6561711e+00	1.5570355e+00	2.8894988e-01	3.1807913e-01	2.8179369e-01	2.6465368e-01	3.7187014e-01
1.3700000e+02	3.5612983e+00	3.5175874e+00	3.9074380e+00	3.7428064e+00	5.4556910e+00	6.1066754e-01	3.8116796e-01	7.9872455e-01	6.1815493e-01	1.1900507e+00

Fig. 3d)

RESIDUE	$\Delta R_{1\rho}$	$\sigma(\Delta R_{1\rho})$
2.0000000e+00	3.5441774e-01	2.3555238e-02
4.0000000e+00	1.7667618e-02	9.1580073e-03
7.0000000e+00	-8.4617450e-03	1.7384883e-02
8.0000000e+00	-4.3160424e-02	2.3751914e-02
1.0000000e+01	8.7851454e-03	8.2210356e-03
1.2000000e+01	-3.4643735e-03	4.6524412e-03
1.3000000e+01	1.4481750e-02	1.4406274e-02
2.4000000e+01	-4.1926600e-02	1.7686619e-02
3.1000000e+01	1.5058992e-02	2.7043482e-02
3.4000000e+01	-1.0105550e-01	2.8894483e-02
3.5000000e+01	-1.4311245e-02	2.3138473e-02
3.7000000e+01	1.3013464e-02	1.5168568e-02
3.8000000e+01	-3.4203420e-02	3.7603945e-02
4.4000000e+01	1.0908660e-02	7.3444489e-03
4.6000000e+01	8.6543712e-02	7.6887461e-03
4.8000000e+01	2.5336432e-01	1.3518610e-02
5.1000000e+01	3.8122931e-02	5.3463180e-03
6.3000000e+01	-5.4195129e-02	2.4158989e-02
6.5000000e+01	-2.9405484e-02	3.1370778e-02
6.9000000e+01	8.8563703e-03	8.7958577e-03
7.1000000e+01	-1.8031173e-02	1.2949894e-02
7.3000000e+01	-5.9047151e-02	8.9954488e-03
7.4000000e+01	-1.4593146e-01	2.3350993e-02
7.7000000e+01	2.5915228e-02	8.2423410e-02
8.7000000e+01	1.1253876e-02	1.6269107e-02
9.1000000e+01	-2.0028284e-02	1.4524661e-02
9.4000000e+01	4.7154113e-02	1.2117952e-02
9.5000000e+01	1.0896719e-02	2.4982586e-02
1.1100000e+02	6.3179636e-03	1.1647195e-02
1.1200000e+02	-4.1263543e-02	6.7569662e-03
1.1300000e+02	-2.8176700e-02	6.6677357e-03
1.1500000e+02	-1.9987211e-02	1.3399524e-02
1.2200000e+02	4.7758357e-02	3.5316923e-02
1.2300000e+02	-4.7441122e-03	1.5224625e-02
1.2800000e+02	-1.2963997e-02	1.3851019e-02
1.3700000e+02	1.1143487e-01	3.9340756e-02

Fig. 4c),e)

RESIDUE	$\rho_1^{(\theta,S)}$	$\rho_4^{(\theta,S)}$	$\rho_5^{(\theta,S)}$	$\sigma(\rho_1^{(\theta,S)})$	$\sigma(\rho_4^{(\theta,S)})$	$\sigma(\rho_5^{(\theta,S)})$
2.0000000e+00	4.1239501e-02	5.2809190e-03	3.9129341e-03	4.0487865e-03	1.2103417e-03	1.0191828e-03
4.0000000e+00	1.3387785e-02	1.8749924e-03	4.0098063e-04	2.0920964e-03	4.0632899e-04	3.5692439e-04
7.0000000e+00	5.6473053e-03	2.3834867e-03	5.4764324e-04	4.3415218e-03	1.1101532e-03	7.6569980e-04
8.0000000e+00	9.0113474e-03	1.5371119e-03	7.2738669e-04	3.5151094e-03	6.9903408e-04	6.3925199e-04
1.0000000e+01	9.1621037e-03	2.0239918e-03	3.7701155e-05	2.0931119e-03	4.8381732e-04	2.5542069e-04
1.2000000e+01	6.8504092e-03	1.1047499e-03	3.3033396e-04	1.5797431e-03	7.8601339e-04	4.5850908e-04
1.3000000e+01	5.6320380e-03	2.1691287e-03	1.5422839e-04	1.0473722e-02	2.1286204e-03	1.0858818e-03
1.7000000e+01	2.4660696e-02	8.0003597e-03	8.2695867e-05	8.5479995e-03	3.1804065e-03	1.5697075e-03
2.4000000e+01	9.2139474e-03	1.5282075e-03	3.6140191e-04	5.5665621e-03	1.0927535e-03	6.4325352e-04
3.4000000e+01	1.9085380e-02	4.6053913e-03	8.4093277e-04	8.4575975e-03	1.7235871e-03	1.3333303e-03
3.5000000e+01	9.6314763e-03	5.1739354e-04	1.4176637e-03	9.6451501e-03	1.3325094e-03	9.2113902e-04
3.7000000e+01	2.2390241e-02	1.0542170e-03	4.5882389e-04	6.8184531e-03	1.5787428e-03	1.0039700e-03
3.8000000e+01	1.1163019e-02	4.6655379e-03	1.1352382e-03	3.5823285e-03	1.6803147e-03	1.1754256e-03
4.4000000e+01	1.1710123e-02	1.2349370e-03	6.5400730e-04	8.2785296e-04	4.1678821e-04	3.3421947e-04
4.6000000e+01	4.8952901e-02	2.4329884e-03	1.6750274e-03	2.6286807e-03	4.4958821e-04	3.5722525e-04
4.8000000e+01	7.1751292e-03	3.1610459e-03	2.2584998e-03	2.3454395e-03	6.4713484e-04	7.0319058e-04
5.1000000e+01	6.0584791e-03	2.3379449e-03	1.0144800e-04	1.5796266e-03	7.2464261e-04	5.7445036e-04
5.7000000e+01	1.2803973e-02	1.8704739e-03	4.1398292e-15	4.9817101e-03	1.1760914e-03	5.9583966e-04
6.9000000e+01	1.1283068e-02	2.3775958e-03	7.4064623e-04	1.7121138e-03	1.0285464e-03	7.1482838e-04
7.1000000e+01	1.0624209e-02	5.8625055e-03	1.1955394e-03	3.8639725e-03	2.8792076e-03	1.7194990e-03
7.3000000e+01	7.2549702e-03	4.0810990e-03	2.1728875e-03	1.5611213e-03	1.3065352e-03	1.2853884e-03
7.4000000e+01	2.5649880e-02	4.2920146e-03	4.0384900e-03	3.6031854e-03	2.5379417e-03	4.2409285e-03
7.7000000e+01	3.6333262e-02	1.8208215e-02	1.0302319e-09	2.0958336e-03	7.3247352e-03	4.2581892e-03
8.7000000e+01	1.1623623e-02	6.0660976e-03	7.1512406e-03	3.4693277e-03	3.7423905e-03	4.5516322e-03
9.1000000e+01	1.2827411e-02	8.6029331e-03	2.5040979e-03	7.2520660e-03	3.2201810e-03	2.2177120e-03
9.3000000e+01	1.1592225e-02	1.6905016e-03	8.9795585e-04	4.0331457e-03	1.8364198e-03	1.1889560e-03
9.4000000e+01	1.8064195e-02	4.0833378e-03	5.9731564e-03	8.1940599e-03	5.6960039e-03	6.4291632e-03
9.5000000e+01	1.1323428e-02	2.7280116e-03	1.0868076e-03	5.3482573e-03	2.5002066e-03	1.5921930e-03
1.0900000e+02	1.9221887e-02	2.1351668e-03	2.3717255e-04	4.6246996e-03	1.2643659e-03	8.0578555e-04
1.1100000e+02	4.5669127e-03	6.6365993e-04	1.3589866e-03	2.9793302e-03	7.5715439e-04	5.7083546e-04
1.1200000e+02	1.7354851e-02	1.5944474e-03	4.3555176e-04	2.2173358e-03	2.7833578e-04	2.1822628e-04
1.1300000e+02	1.7569492e-02	1.5105419e-03	3.3765320e-04	1.1484621e-03	2.9631526e-04	1.8956666e-04
1.1500000e+02	7.8384562e-03	1.8908207e-03	6.6083583e-04	1.9850988e-03	1.8216652e-03	1.1187341e-03
1.2800000e+02	9.3145177e-03	2.0971115e-03	5.3980930e-04	2.0426952e-03	7.0673894e-04	7.2552572e-04
1.3700000e+02	1.8754560e-02	6.4830790e-03	2.9312683e-04	4.2337141e-03	1.8303508e-03	1.2010051e-03

Fig. 4d)

RESIDUE	$\rho_2^{(\theta,S)}$	$\rho_3^{(\theta,S)}$	$\sigma(\rho_2^{(\theta,S)})$	$\sigma(\rho_3^{(\theta,S)})$
2.0000000e+00	1.7349626e-03	9.9868359e-03	5.2184466e-03	5.4149940e-03
3.0000000e+00	2.2187293e-03	2.3351780e-04	1.3080321e-03	1.0737931e-03
4.0000000e+00	2.4708096e-03	1.2518896e-10	1.4175138e-03	1.1461208e-03
7.0000000e+00	6.9241793e-04	3.9863190e-04	1.2809375e-03	9.6112823e-04
8.0000000e+00	7.0660787e-13	6.0283636e-04	7.3387381e-04	6.9347199e-04
1.0000000e+01	1.0691362e-03	1.9915198e-04	7.9962588e-04	7.1868910e-04
1.1000000e+01	1.7807221e-03	6.2838713e-13	9.2465794e-04	7.8927226e-04
1.2000000e+01	1.3597862e-03	1.7278011e-09	6.0448303e-04	5.9424053e-04
1.3000000e+01	2.3461823e-14	1.8006724e-04	5.4154787e-04	4.4392833e-04
1.5000000e+01	4.5142340e-11	1.8235625e-03	1.4720988e-03	1.8487643e-03
2.1000000e+01	2.4591484e-03	3.0190964e-04	1.8124159e-03	1.5424352e-03
2.2000000e+01	2.7893405e-03	9.7716104e-10	1.3352066e-03	1.2448101e-03
2.3000000e+01	1.8367469e-03	2.0571192e-04	1.8382549e-03	1.6437323e-03
2.6000000e+01	5.9680949e-09	1.8526089e-03	1.3849944e-03	1.4632078e-03
2.7000000e+01	1.0708612e-03	7.2747061e-04	1.4698686e-03	1.2945494e-03
2.8000000e+01	8.1105182e-04	3.5173128e-03	1.3868018e-03	1.4633169e-03
2.9000000e+01	1.7386107e-03	7.8440731e-11	1.4316080e-03	1.1402820e-03
3.2000000e+01	1.7026910e-03	2.1592882e-11	1.7116337e-03	1.2224241e-03
3.3000000e+01	2.1154707e-03	7.9663382e-13	1.1231619e-03	1.0337536e-03
3.5000000e+01	1.5293830e-03	1.3628153e-13	1.6994049e-03	1.3292305e-03
3.6000000e+01	3.5072039e-06	1.7510788e-03	1.2702913e-03	1.7643828e-03
3.8000000e+01	7.8697381e-13	1.9686905e-03	1.2308960e-03	1.5817658e-03

3.9000000e+01	3.6530080e-04	5.6207230e-15	5.0869824e-04	3.8839360e-04
4.0000000e+01	4.2891990e-13	1.3199780e-03	1.1448316e-03	1.2198468e-03
4.1000000e+01	1.2189377e-14	9.7611393e-04	8.2814651e-04	6.3807916e-04
4.2000000e+01	1.0227800e-03	1.7167489e-04	1.1401031e-03	9.3738503e-04
4.3000000e+01	2.4441938e-13	1.6267495e-03	1.6106243e-03	1.7059384e-03
4.4000000e+01	1.1361991e-03	1.1307350e-04	7.3856968e-04	6.3932772e-04
4.6000000e+01	2.4995875e-03	7.5537768e-13	7.7951336e-04	6.3349706e-04
4.8000000e+01	2.0701869e-03	1.0146152e-03	7.3882766e-04	7.7712286e-04
4.9000000e+01	1.8168883e-03	1.1803400e-03	1.3333711e-03	1.5648923e-03
5.1000000e+01	1.3485102e-03	4.4324623e-12	9.8507958e-04	7.9764569e-04
5.2000000e+01	1.2725484e-03	3.1695349e-11	7.7958186e-04	8.5169238e-04
5.3000000e+01	1.7851470e-03	8.4522890e-14	1.0508597e-03	9.4511577e-04
5.5000000e+01	3.6422084e-03	1.6182374e-11	3.7503156e-03	3.5233197e-03
5.6000000e+01	2.5203453e-03	2.8914485e-12	1.5511490e-03	1.2822741e-03
6.0000000e+01	2.4143297e-03	8.2089171e-10	1.6394876e-03	1.4933774e-03
6.3000000e+01	3.5838414e-14	1.6242058e-03	1.5864060e-03	1.2345353e-03
6.4000000e+01	1.8234570e-03	6.4838243e-11	1.3827913e-03	1.2918655e-03
6.6000000e+01	3.5955754e-10	1.1653230e-03	1.3162812e-03	1.3577076e-03
6.7000000e+01	1.2158771e-03	1.4000390e-03	1.4354012e-03	1.4077187e-03
6.8000000e+01	1.2938614e-03	2.8137752e-03	3.0460391e-03	2.8528539e-03
7.0000000e+01	1.7577807e-03	3.9359234e-04	1.8951431e-03	1.5581147e-03
7.1000000e+01	6.4449480e-04	2.1955450e-03	1.8093136e-03	1.7461004e-03
7.2000000e+01	9.9505714e-03	4.1647300e-09	5.4833932e-03	4.2704613e-03
7.3000000e+01	2.2828525e-03	1.5565324e-03	1.3178926e-03	1.2977996e-03
7.4000000e+01	5.4359143e-03	5.9702466e-12	1.5744310e-03	1.5465393e-03
7.5000000e+01	3.7015864e-03	1.3070589e-10	2.6962557e-03	2.0667466e-03
7.6000000e+01	2.1982821e-03	4.1530272e-03	4.4578553e-03	4.3735685e-03
7.7000000e+01	1.1440147e-02	1.6110345e-03	7.4174966e-03	6.9852687e-03
7.8000000e+01	1.2275409e-03	7.5566959e-03	5.0134447e-03	4.6313846e-03
8.0000000e+01	3.1344609e-03	3.4479299e-14	1.0296127e-03	8.7136541e-04
8.1000000e+01	2.9889469e-03	1.5457610e-03	2.1889311e-03	1.8695063e-03
8.2000000e+01	3.5082449e-03	8.9591882e-11	1.8924255e-03	1.6115161e-03
8.3000000e+01	4.5083737e-03	1.0099479e-12	3.2657583e-03	3.0372589e-03
8.4000000e+01	1.8706415e-11	3.0573962e-03	1.9962022e-03	2.0179648e-03
8.5000000e+01	3.7845055e-03	1.6500853e-09	1.8826982e-03	1.5531638e-03
8.7000000e+01	1.3898455e-03	2.5835724e-03	2.6991395e-03	2.4749927e-03
8.8000000e+01	2.9504194e-03	3.1446833e-09	1.1043527e-03	1.0352251e-03
8.9000000e+01	4.0448896e-03	3.7023488e-11	1.1046070e-03	1.1018885e-03
9.0000000e+01	1.6475744e-03	1.4613602e-03	2.9929182e-03	2.4727771e-03
9.1000000e+01	1.4818983e-03	1.4225825e-03	2.3992531e-03	2.0808585e-03
9.2000000e+01	1.4529957e-03	2.8317710e-03	2.3608761e-03	2.7766400e-03
9.3000000e+01	1.4601717e-09	1.9203025e-03	2.2224486e-03	2.2352979e-03
9.4000000e+01	8.1116079e-13	2.7626894e-03	8.2918846e-04	8.2771739e-04
1.0900000e+02	2.4668212e-03	2.3821655e-13	1.7568428e-03	1.6908683e-03
1.1100000e+02	1.0684536e-03	9.8575750e-04	7.4097740e-04	8.6818267e-04
1.1200000e+02	1.4152296e-03	7.9019878e-04	1.1333021e-03	8.9861094e-04
1.1300000e+02	1.3579370e-03	7.6404582e-11	1.0333791e-03	9.4163698e-04
1.1400000e+02	8.1961185e-04	1.2885795e-03	1.5006802e-03	1.4029846e-03
1.1500000e+02	2.9036092e-09	1.7745688e-03	1.7570835e-03	1.5926299e-03
1.1600000e+02	2.6851111e-03	9.1453076e-10	1.5791399e-03	1.2365671e-03
1.1700000e+02	2.0005369e-03	5.0766513e-13	1.9092503e-03	1.4464752e-03
1.2300000e+02	1.6968575e-03	1.1374248e-03	2.3579721e-03	2.1323505e-03
1.2800000e+02	2.3714944e-03	1.4051429e-10	2.2040627e-03	2.0411584e-03
1.3100000e+02	2.5549352e-03	4.7134631e-11	2.3864959e-03	1.8139776e-03
